# Tellurolate: an effective Te-atom transfer reagent to prepare the triad of group 5 metal bis(tellurides)†

**DOI:** 10.1039/d3sc03470d

**Published:** 2023-10-05

**Authors:** Shuruthi Senthil, Seongyeon Kwon, Richard Y. Kong, Samantha N. MacMillan, Pavel Zatsepin, Michael R. Gau, Patrick J. Carroll, Mu-Hyun Baik, Kyle M. Lancaster, Daniel J. Mindiola

**Affiliations:** a Department of Chemistry, University of Pennsylvania Philadelphia PA 19104 USA mindiola@sas.upenn.edu; b Department of Chemistry, Korea Advanced Institute of Science and Technology (KAIST) Daejeon 34141 Republic of Korea mbaik2805@kaist.ac.kr; c Center for Catalytic Hydrocarbon Functionalizations, Institute for Basic Science (IBS) Daejeon 34141 Republic of Korea; d Department of Chemistry and Chemical Biology, Cornell University Ithaca New York 14853 USA kml236@cornell.edu

## Abstract

We show in this work how lithium tellurolate Li(X)_*n*_TeCH_2_SiMe_3_ (X = THF, *n* = 1, 1; X = 12-*crown*-4, *n* = 2, 2), can serve as an effective Te-atom transfer reagent to all group 5 transition metal halide precursors irrespective of the oxidation state. Mononuclear and bis(telluride) complexes, namely (PNP)M(Te)_2_ (M = V; Nb, 3; Ta, 4; PNP^−^ = N[2-P^i^Pr_2_-4-methylphenyl]_2_), are reported herein including structural and spectroscopic data. Whereas the known complex (PNP)V(Te)_2_ can be readily prepared from the trivalent precursor (PNP)VCl_2_, two equiv. of tellurolate, and elemental Te partially solubilized with PMe_3_, complex 3 can also be similarly obtained following the same procedure but with or without a reductant, Na/NaCl. Complex 4 on the other hand is formed from the addition of four equiv. of tellurolate to (PNP)TaF_4_. Having access to a triad of (PNP)M(Te)_2_ systems for group 5 metals has allowed us to compare them using a combination of theory and spectroscopy including Te-L_1_ edge XANES data.

## Abstract 2

We show in this work how lithium tellurolate Li(X)_*n*_TeCH_2_SiMe_3_ (X = THF, *n* = 1, 1; X = 12-*crown*-4, *n* = 2, 2), can serve as an effective Te-atom transfer reagent to all group 5 transition metal halide precursors irrespective of their oxidation state.

## Graphical abstract

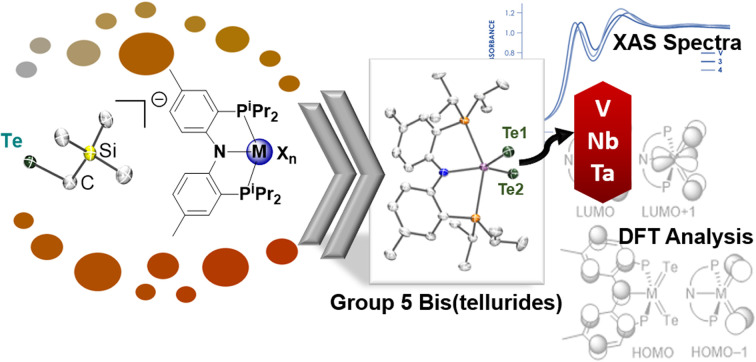

## Introduction

**Fig. 1:**
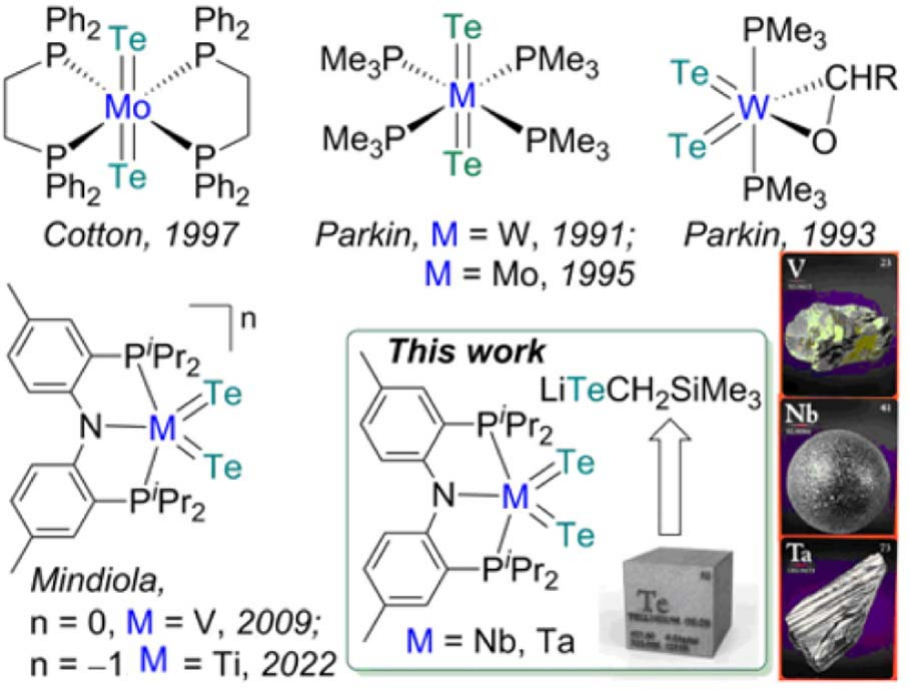
(Top) Examples of bis(tellurides) of Mo and W. (Bottom) Examples of 3d metals along with group 5 bis(tellurides). A β-diketiminate analogue 
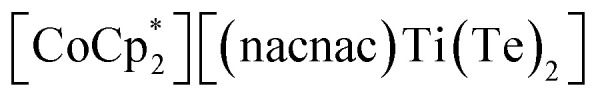
 (nacnac^−^ = [ArN(CH_3_)]_2_CH, Ar = 2,6-^i^Pr_2_C_6_H_3_) has also been reported.

In comparison to the development of transition metal complexes with multiple bonds to the lighter congeners of group 16 elements such as oxides and sulfides, analogous examples of the heavier elements, selenides and tellurides, remain far more underdeveloped.^1^ The tendency of these heavy elements to bridge or catenate,^1*b*^ their large difference in σ- and π-bond energies (which results in their weak π overlap to transition metals),^1*a*^ and the lack of soluble and convenient synthetic routes to deliver these atoms render such moieties L_*n*_M=X (X = Se, Te) difficult to access in well-defined complexes.^1*a*,*b*^ Furthermore, metal complexes with two terminal telluride ligands (bis(tellurides)) are even rarer with the few structurally characterized examples mostly being confined to group 6 metals: Mo(L)_2_(Te)_2_ (L = dppee, dppe),^2^ M(PMe_3_)_4_(Te)_2_ (M = W,^3^ Mo^4^) and W(PMe_3_)_2_(Te)_2_(η^2^-OCHR) (R = H, Ph)^5^ (top, Fig. 1). Previously, we reported the synthesis and reactivity of the first 3d transition metal bis(telluride), (PNP)V(Te)_2_ (PNP^−^ = N[2-P^i^Pr_2_-4-methylphenyl]_2_, bottom, Fig. 1), from (PNP)V(CH_2_^*t*^Bu)_2_^6^ and Te, and showed how the bis(telluride) complex was a masked form of “(PNP)V” fragment in the presence of various oxidants.^7^ More recently, we synthesized a bis(telluride) titanate, 
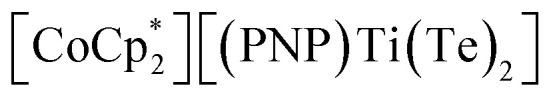
 (bottom left, Fig. 1) starting from the trivalent bis(alkyl) complex,^8^ and probed its ability to act as a metallo bis(telluride) ligand.^9^

Since transition metal bis(telluride) motifs remain quite scant and given our breakthrough with 3d metals, vanadium and titanium,^7,9^ we inquired if such functionality can be extended down the group to niobium and tantalum by using a similar protocol. Unfortunately, the respective low-valent niobium or tantalum hypothetical bis or trisalkyl complexes are inherently unstable, and in the case of Nb, these ligands readily undergo disproportionation and/or β-hydrogen abstraction/hydrogen elimination reactions^10^ resulting in the bis(alkylidene) complex, (PNP)Nb(=CH^*t*^Bu)_2_.^10*b*^ Therefore, we decided to explore a systematic approach to delivering the Te atom that would work irrespective of the oxidation state of the metal since the stability of group 5 transition metal halide precursors tend to differ depending on their periodic trends. For instance, V^III^ chloride (*e.g.*, VCl_3_(THF)_3_) is a common starting material, whereas NbCl_4_(THF)_2_/NbCl_5_ and TaCl_5_ are routinely used for heavier congeners. Inspired by the decomposition of M(TeR)_*n*_ to [MTe]_*n*_ from solid state thermolysis,^11^ we hypothesized that terminal metal bis(telluride) ligands could likewise be assembled *via* the elimination of telluroethers,^11*a*,*b*,12^ akin to how Arnold and co-workers prepared a Ta=Te mono(telluride) complex using silyltelluroether as the leaving group.^12*e*^

Herein we demonstrate how an easily prepared tellurolate salt [Li(THF)TeCH_2_SiMe_3_]_*x*_, can be an effective Te-atom transfer reagent not only to V^III^, but also to Nb^IV^ and Ta^V^ halide precursors. Our approach demonstrates that low or high-valent precursors, which are generally more common for the lighter or heavier group 5 metals, respectively, can all be adequate reagents for the assembly of the bis(telluride) motif when using [Li(THF)TeCH_2_SiMe_3_]_*x*_ and if needed, an appropriate oxidant or reductant.

## Results and discussion

**Scheme 1:**
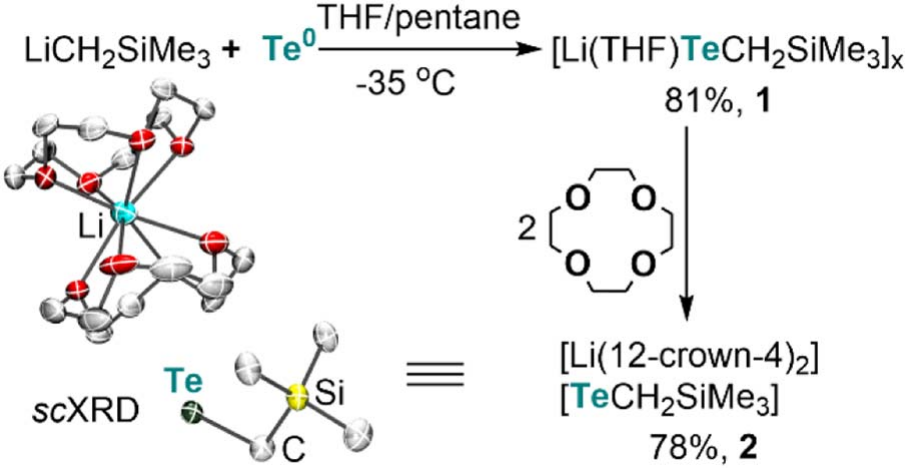
Synthesis of tellurolate salts and the solid-state structure of the discrete tellurolate [Li(12-*crown*-4)_2_][TeCH_2_SiMe_3_] (2) with thermal ellipsoids at the 50% probability level and with H-atoms omitted for clarity.

Lithium alkyltellurolate [Li(THF)TeCH_2_SiMe_3_]_*x*_ (1) could be readily prepared by slow and dropwise addition of elemental tellurium as a suspension in THF to a cold pentane solution of recrystallized and commercially available LiCH_2_SiMe_3_ (−35 °C). Despite no single-crystal X-ray diffraction (sc-XRD) data for 1, NMR spectroscopic analysis of the isolated product corroborates its formation in 81% yield^13^ as a pale yellow residue (Scheme 1).

To obtain solid-state structural information by sc-XRD, we prepared the discrete salt, [Li(12-*crown*-4)_2_][TeCH_2_SiMe_3_] (2), in 78% yield *via* the addition of two equiv. of 12-*crown*-4 to the tellurolate solution at −35 °C (Scheme 1). Although sterically hindered aryl and silyl tellurolates are known,^14^ structurally characterized alkyl tellurolate is limited to just one example: dimeric [Li(TMEDA)Te^*n*^Bu]_2_,^15^ thus making [TeCH_2_SiMe_3_]^−^ a rare example of a one coordinate alkyl tellurolate anion. The Te–C bond distance in 2 (2.196(4) Å) is similar to the dimeric [Li(TMEDA)Te*^n^*Bu]_2_ (2.191(4) Å).^15^ A sharp ^125^Te{^1^H} resonance was located for 1 at −1790 ppm (Fig. S6†)^14*c*^ whereas a broad resonance at −569 ppm (Δ*ν*_1/2_ = 282 Hz) was found for 2 (Fig. S14†).^16^ However, no ^7^Li–^125^Te coupling was resolved in 1 (Fig. S7†), which can be attributed to its fluxional behavior in solution.^16^

Having the tellurolate 1 in hand, we turned our attention to independently making the bis(telluride) complex (PNP)V(Te)_2_ using the V^III^ dichloride precursor, (PNP)VCl_2_.^17^ Accessing (PNP)V(Te)_2_ directly from the dichloride precursor offers two advantages: (1) this circumvents the need to prepare the thermally unstable bisalkyl complex [(PNP)V(CH_2_^*t*^Bu)_2_], which is known to not only activate N_2_,^10*a*^ but also convert to the transient alkylidene [(PNP)V=CH^*t*^Bu] that has been shown to intermolecularly activate C–H bonds in C_6_H_6_,^18^ and (2) avoids the cumbersome synthetic route involving the reaction of [(PNP)V(CH_2_^*t*^Bu)_2_] with elemental Te under a static vacuum at 90 °C for four days.^7^ However, in order to convert V^III^ to V^V^, Te (solubilized with a catalytic amount of PMe_3_) is required as a two-electron oxidant. Treatment of (PNP)VCl_2_ with two equiv. of 1 in the presence of Te/PMe_3_ for 16 hours resulted in the gradual formation of (PNP)V(Te)_2_ which could be isolated from the mixture in 73% yield as a dark green, and highly insoluble material in most common organic solvents.

**Scheme 2:**
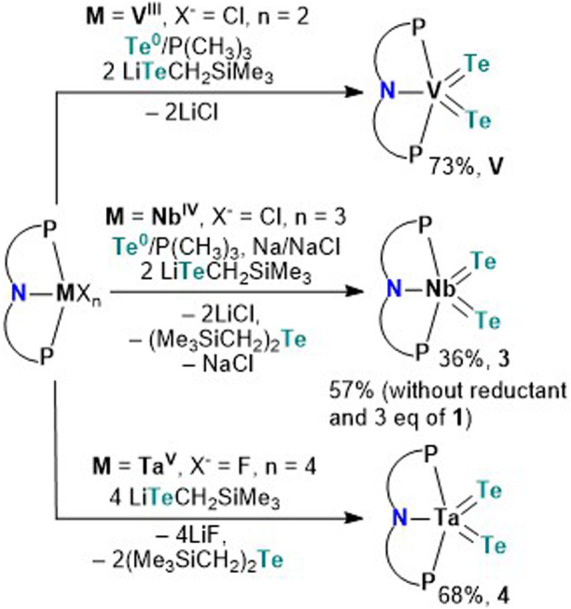
Synthesis of the bis(tellurides) with the respective metal halide precursor to form complexes (PNP)M(Te)_2_ (M = V; Nb, 3; Ta, 4). The PNP ligand is shown with a simplified cartoon omitting ^i^Pr and aryl backbone groups.

Unlike V, Nb poses a challenge since the putative trivalent precursor “(PNP)NbCl_2_” is unstable.^10*b*^ As a result we used instead the Nb^IV^ precursor (PNP)NbCl_3_^10*c*^ under similar conditions in combination with tellurolate 1, but in the presence of a reductant. Accordingly, treatment of (PNP)NbCl_3_ with two equiv. of 1,^19^ in the presence of Te/PMe_3_ and one equiv. of Na/NaCl in THF resulted in the formation of (PNP)Nb(Te)_2_ (3) as a dark green colored crystalline material isolated in 36% yield after work-up of the reaction mixture (Scheme 2). We found later that complex 3 could also be prepared using instead 3 equiv. of 1, and in the presence of Te/PMe_3_ in 57% yield (Scheme 2). Additionally, formation of Te(CH_2_SiMe_3_)_2_^20^ is confirmed in both reactions by ^1^H and ^125^Te NMR spectroscopy (Fig. S24 and S25†).^13^

Analogous to Arnold's construction of a terminal mono(telluride) ligand onto Ta^V^ dihalide,^12*e*^ one should intuitively conceive the assembly of a bis(telluride) motif using the appropriate Ta^V^ tetrahalide precursor. Conveniently, Ozerov and co-workers have reported a Ta^V^ starting material, namely (PNP)TaF_4_.^21^ Treating (PNP)TaF_4_ with 4 equiv. of 1 in THF over 16 hours at room temperature resulted in the formation of (PNP)Ta(Te)_2_ (4) in 68% yield as a red, crystalline material (Scheme 2). Examination of the side-product formed from this reaction *via*^1^H and ^125^Te NMR spectroscopy again verified the formation of Te(CH_2_SiMe_3_)_2_ along with other intractable products (Fig. S33 and S34†).^13^ Interestingly, the loss of Te(CH_2_SiMe_3_)_2_ was not observed in the formation of V. It is noteworthy that in all three cases, the reduction of metal halides (PNP)MX_*n*_ (M = V, Nb, Ta; X = Cl, F; *n* = 2, 3, 4) in the presence of Te(0) did not provide species (PNP)M(Te)_2_, highlighting the utility of 1 as a Te-atom delivery reagent.

**Fig. 2:**
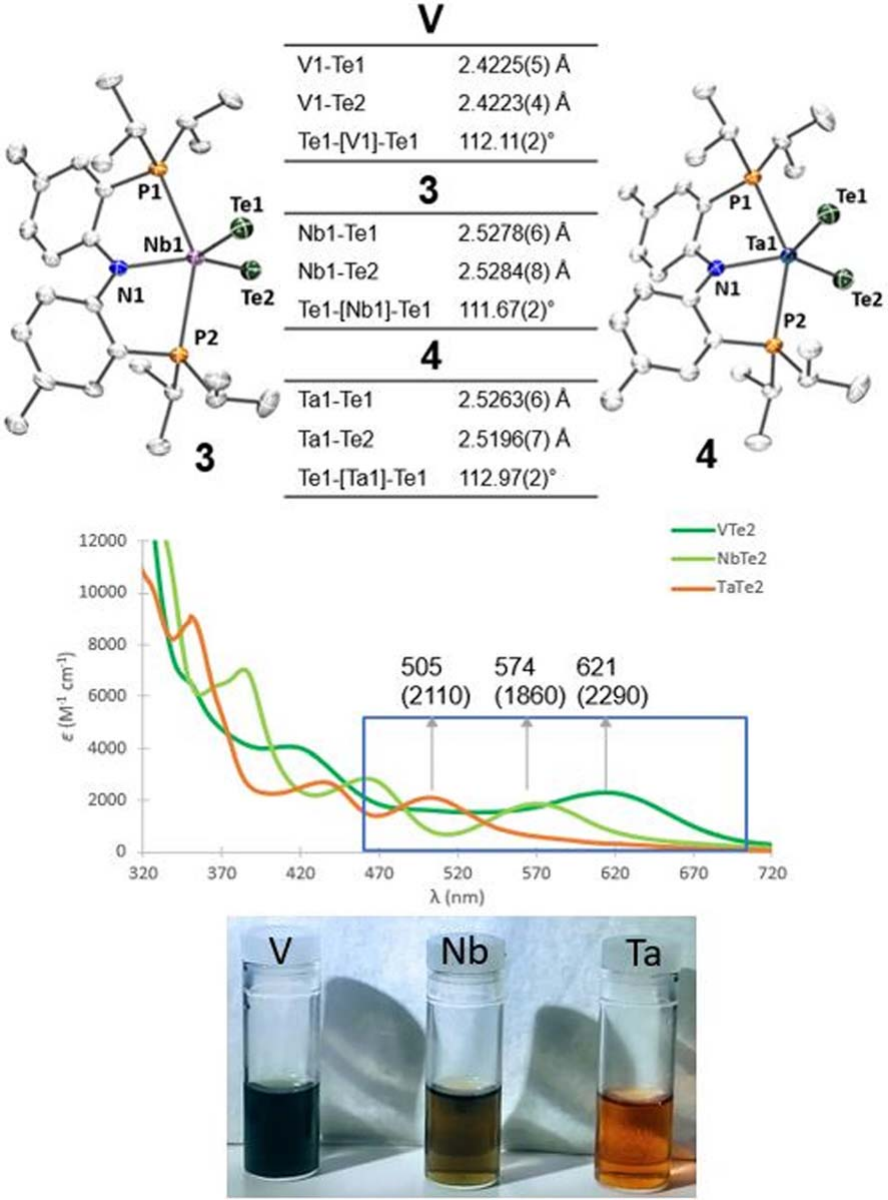
(Top) Solid-state structures of complexes 3 (left) and 4 (right) with thermal ellipsoids at 50% probability level. Cocrystallized THF is omitted for clarity. (Middle) UV-vis absorption spectra of complexes 3 and 4 (0.056 mM) in THF; molar absorptivities (M^−1^ cm^−1^) are in parentheses. (Bottom) Vials containing THF solutions of V, 3, and 4.

Single crystals of compounds 3 and 4 were obtained from a THF/pentane mixture at −35 °C as green and red needles respectively, and sc-XRD confirmed the formation of isomorphous five-coordinate bis(telluride) structures (top, Fig. 2). The M=Te bond lengths in 3 and 4 are similar to the anionic Ti bis(telluride) complex, [(PNP)Ti(Te)_2_]^−^ (2.5185(6) Å),^9^ but longer than that of (PNP)V(Te)_2_^7^ owing to the increase in ionic radii of the metal as one descends the group. To date, the only documented example of a terminally bound telluride ligand to Nb and Ta include the mono(telluride) complexes, [(C_5_Me_5_)Nb(NAr)(Te)(PMe_3_)] (Ar = 2,6-C_6_H_3_^i^Pr_2_; not structurally characterized),^22^ [((Me_3_SiNCH_2_CH_2_)_3_N)Ta(Te)] (Ta=Te, 2.568(1) Å),^12*e*^ and 
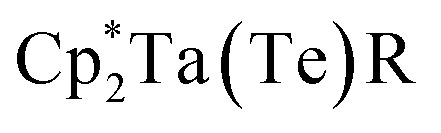
 (R = H, Ta=Te, 2.588(2) Å),^23^ reported by Gibson, Arnold, and Parkin respectively. Notably, the Ta=Te bond distance in 4 is shorter than Arnold's and Parkin's high-valent mono(telluride) complexes. The ^1^H and ^31^P NMR spectroscopic features for 3 and 4 are indicative of a *C*_2_-symmetric complex in solution with one ^31^P singlet resonance for the symmetrically equivalent phosphorus atoms (V: 142 ppm, 3: 91 ppm and 4: 111 ppm). Markedly, the ^125^Te{^1^H} NMR spectrum shows a broad resonance at 3896 ppm for 3 (Δ*ν*_1/2_ = 404 Hz) and 3041 ppm for 4 (Δ*ν*_1/2_ = 370 Hz) whereas the titanium bis(telluride) anion, [(PNP)Ti(Te)_2_]^−^ (3260 ppm, Δ*ν*_1/2_ = 560 Hz) is in between 3 and 4. All bis(telluride) complexes in this work are air-sensitive, highly colored (V: dark green, Nb: green in solution, Ta: red; bottom, Fig. 2), stable as a solid up to 180 °C,^13^ and show a modest increase in solubility down the group. The latter feature is counterintuitive as one would expect the Ta=Te bond in 4 to be more ionic and thus less soluble relative to Nb=Te in 3 and V=Te in (PNP)V(Te)_2_ based on periodic trends. To probe the electronic features in these complexes, we collected UV-vis absorption spectra in THF at the same concentrations (0.056 mM). In addition to the high energy ligand-to-ligand transitions, the spectra show the expected hypsochromic shift in absorptions down the group: (PNP)V(Te)_2_ (*λ* = 621 nm, *ε* = 2290 M^−1^ cm^−1^); 3 (574 nm, 1860 M^−1^ cm^−1^); 4 (505 nm, 2110 M^−1^ cm^−1^), that can be ascribed to ligand-to-metal charge transfer (LMCT) transitions (middle, Fig. 2, and S36–S39†).

**Fig. 3:**
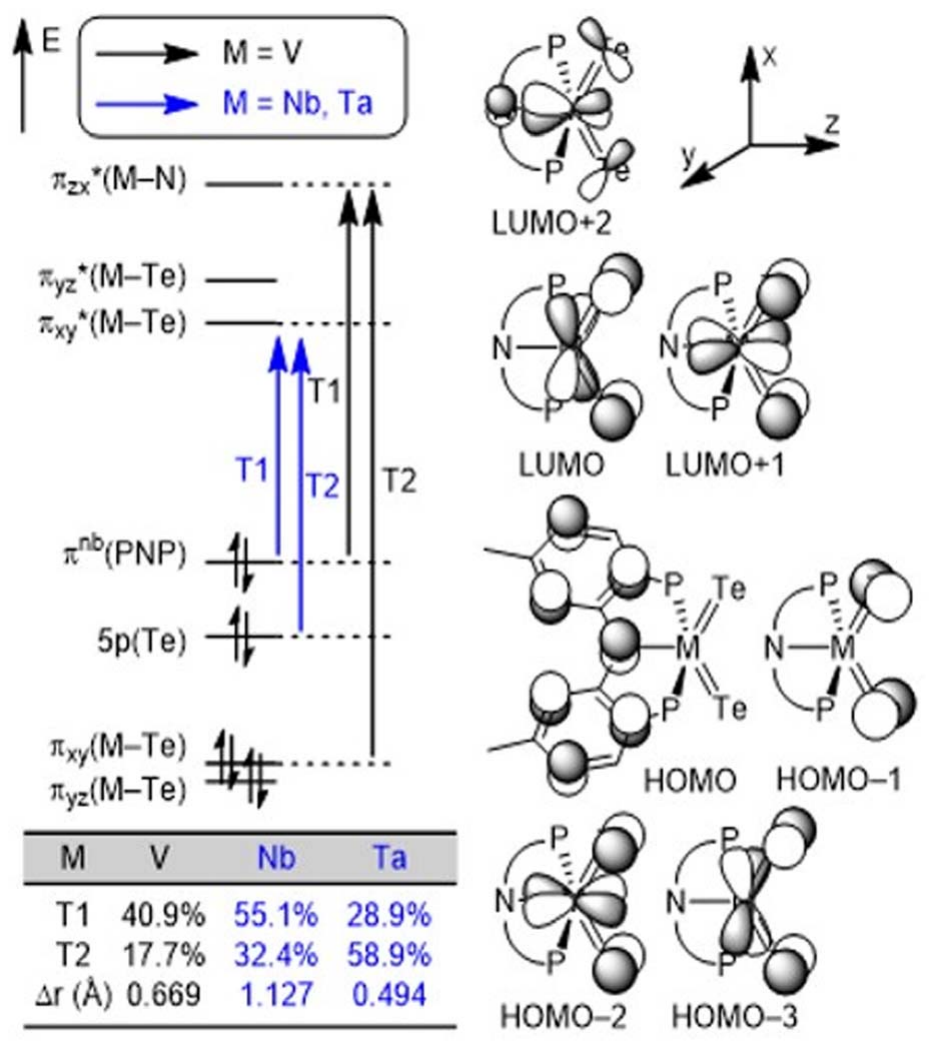
Conceptual MO diagrams of (PNP)M(Te)_2_ (M = V, Nb, and Ta) complexes and the most contributing orbital transitions denoted as T1 and T2, respectively.

We conducted density functional theory (DFT) calculations to better understand the nature of M=Te bonds in the series. After an extensive DFT functional benchmark study (Fig. S40–S42†), we used the MN15/TZ2P//DZP level of theory^24^ implemented in the Amsterdam Density Functional (ADF) software^25^ package to compute and analyze charge-transfer characters. Fig. 3 illustrates the frontier orbitals of the three complexes and the orbital transitions involved in the excitation. The HOMO–LUMO gap increases down the group in accord with the periodic trends: 3.652 eV (V), 3.942 eV (Nb) and 4.201 eV (Ta). Two frontier orbital transitions corresponding to the experimentally observed absorptions^13^ were identified where the T1 transitions involve the pincer-to-metal charge transfer from HOMO to LUMO (M = Nb and Ta) or LUMO+2 (M = V). In contrast, the T2 transitions are mostly localized on the metal center (M = V) or the telluride moiety (M = Nb and Ta) depending on the charge transfer character (Fig. S43†). The table inset in Fig. 3 clearly illustrates that the contribution of T1 follows the expected trend based on the average hole–electron distance, Δ*r*,^26^ obtained from natural transition orbital (NTO) analysis. We performed a Hirshfeld charge analysis to probe the bond polarity whereby the Nb center (+0.35*e*) in 3 (Te: −0.19*e*) is more electron-deficient relative to Ta (+0.23*e*) in 4 (Te: −0.17*e*) which results in an increased polarization in the binding of the ligands to the transition metal. This difference supports the increased solubility observed in the Ta complex relative to Nb. Furthermore, we hypothesize that the slight decrease in the positive charge of Ta in 4 can be attributed to relativistic contractions of the p orbitals, making the 5p orbitals of Ta more electronegative compared to the 4p orbitals of Nb.

**Fig. 4:**
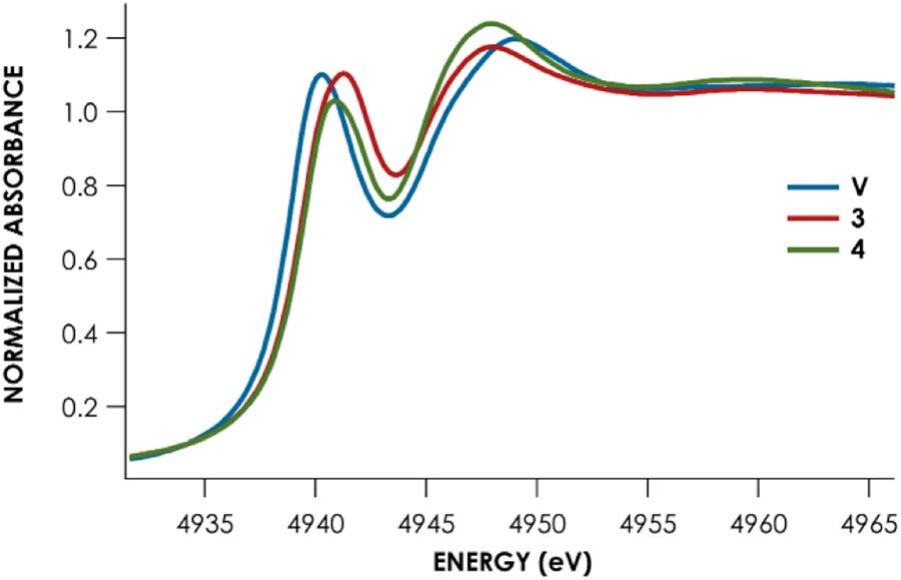
Te-L_1_ edge XANES data for complexes V (blue), 3 (red), and 4 (green).

Lastly, Te L_1_-edge X-ray absorption spectra (XAS) were obtained for compounds V, 3, and 4 (Fig. 4 and Table 1). TDDFT calculations^13^ of the Te L_1_-edge XAS spectrum were performed using geometries derived from the respective sc-XRD-derived structures. Good agreement is observed between the experimental Te L_1_ spectra and the calculated spectra (Fig. S50–S52†).^13^ In all three cases, the L_1_ peak corresponds to a Te 2s → Te 5p transition with varying M d and ligand-based orbital contributions. In both the calculated and measured spectra, the L_1_ edge peak for V is red-shifted relative to 3 and 4 which are measured to be essentially coincident with each other. This likely reflects the more contracted V 3d orbitals, compared to Nb and Ta 4d and 5d orbitals respectively. This results in poor M d/Te p overlap and ultimately in greater charge density localized on the Te atoms. This is supported by inspection of the Löwdin calculated charges: Te is calculated to have an average charge of −0.21*e* in V, whereas Te is calculated to have an average charge of +0.09*e* and +0.07*e* in 3 and 4, respectively. However, we found that irrespective of the charge analysis method used (Hirshfeld *vs.* Löwdin), the metal center is more electropositive than the Te atom. Inspection of the empty frontier orbitals of V, 3, and 4 (Table S9†) shows that there is more TM d orbital character than Te indicative of overall nucleophilic character on Te and positive character on the metal center.

**Table 1:** Calculated and measured Te L_1_ peak data

Complex	Measured L_1_ peak (eV)	Measured L_1_ peak area	Calculated L_1_ peak^a^ (eV)
V	4940.3 ± 0.01	5.9 ± 0.5	4940.6
3 (Nb)	4941.1 ± 0.02	6.6 ± 0.5	4940.9
4 (Ta)	4941.1 ± 0.03	6.0 ± 0.6	4941.0

a Calculated as the intensity weighted average position: *E*_peak_ = (∑(*E*_abs_ × *I*_abs_)/∑(*I*_abs_)), where *E*_abs_ is the calculated energy of the state contributing to the peak, and *I*_abs_ is the calculated intensity.

Remarkably, little variance in the Te 5p character of the acceptor orbitals is observed across the measured series (24–27%). This is reflected in the fact that no discernible trend can be observed in the measured Te L_1_ peak area – all areas are within error of each other (Table 1). The transition metal M d character is observed to decrease down the group (V > Nb > Ta). Concomitantly, the acceptor orbitals appear to have increasing ligand character (Ta > Nb > V). Taken together, we can observe that the bonding of Te to group 5 transition metal centers is substantively similar down the group and that varying the metal identity primarily changes the binding of the transition metal to the PNP scaffold, rather than inducing noteworthy changes to the M=Te bonds.

## Conclusions

In summary, we have established a convenient one-step synthetic route to bis(telluride) complexes of group 5 transition metals. In contrast to the protocol of using elemental Te or TePR_3_ to access bis(telluride) motifs, this work presents the groundwork that a lithium alkyl tellurolate can be used to access the rare bis(telluride) species regardless of the starting metal halide complexes and their oxidation state. By combining spectroscopic and theoretical investigation, we identified the electronic structures of the series of complexes and their excitation profiles in the UV-vis region. Outside of the expected periodic trends, Hirshfeld charge analysis indicates increased polarity between Nb=Te bonds relative to Ta=Te, and the Te-L_1_ XAS peak areas are indifferent between these complexes indicating similarity in the bonding between Te and the respective metal center. We are now investigating this strategy for the synthesis of other terminal bis(chalogenides) (*e.g.* Se and/or S) and of transition metals outside of group 5.

## Data availability

All the experimental and computational data are available in the ESI: experimental details, characterization and analytical data. CCDC data available for 2262094 (complex 2), 2262095 (complex 3), and 2262098 (complex 4).†

## Author contributions

SS: conceptualization, formal analysis, data curation, writing – original draft preparation, writing – review & editing; SK: methodology, formal analysis, data curation, writing – original draft preparation, writing – review & editing; RYK: methodology, formal analysis, funding acquisition, data curation, writing – original draft preparation, writing – review & editing; SNM: methodology, writing – review & editing; PZ: conceptualization, writing – review & editing; MRG: data curation; PJC: data curation; MHB: funding acquisition, writing – review & editing, supervision; KML: funding acquisition, writing – review & editing, supervision; DJM: conceptualization, funding acquisition, writing – original draft preparation, writing – review & editing, supervision.

## Conflicts of interest

There are no conflicts to declare.

## Supplementary Material

SC-014-D3SC03470D-s001

SC-014-D3SC03470D-s002
