# Supplementary material for: Tellurolate: an effective Te-atom transfer reagent to prepare the triad of group 5 metal bis(tellurides)
Source: Chem Sci. 2023 Oct 5;14(43):12277–82. doi: 10.1039/d3sc03470d (PMC10631448; doi:10.1039/d3sc03470d)
Supplement: SC-014-D3SC03470D-s001 [file SC-014-D3SC03470D-s001.pdf]

Electronic Supplementary Material (ESI) for Chemical Science.
This journal is © The Royal Society of Chemistry 2023

Supporting Information

Tellurolate: An Effective Te-Atom Transfer Reagent to Prepare the Triad of Group 5 Metals Bis(telluridos)

Shuruthi Senthil, Seongyeon Kwon, Richard Y. Kong, Samantha N. MacMillan, Pavel Zatsepin, Michael R. Gau, Patrick J. Carroll, Mu-Hyun Baik,* Kyle M. Lancaster,* and Daniel J. Mindiola*

Table of Contents

**General Procedure**	S3
Synthesis of Li(THF)$TeCH_2SiMe_3$ (**1**) and Li(12-crown-4)$_2TeCH_2SiMe_3$ (**2**)	S3
Synthesis of (PNP)Nb$(Te)_2$ (**3**)	S4
Synthesis of (PNP)Ta$(Te)_2$ (**4**)	S5
Synthesis of (PNP)V$(Te)_2$ (**V**)	S6
**NMR Spectroscopy**	
Complex **1**	S7-S13
Complex **2**	S14-S20
Complex **3**	S21-S27
Complex **4**	S28-S34
NMR Yield Determination	S35-S36
**UV-vis Spectroscopy**	S36-S38
**X-Ray Crystallography**	S39-S42
**Computational Details**	S43-S55
**X-Ray Absorption Spectroscopy**	S56-S63
**References**	S64-S65

General Procedure

All operations were performed in a M. Braun glove box or using standard Schlenk techniques under a nitrogen atmosphere. Anhydrous solvents (toluene, Et_2O, THF, pentane) were purchased from Fisher Scientific or Aldrich. All anhydrous solvents were purified and dried by passage through two columns of activated alumina and Q-5 drying agent in a Grubbs-type solvent system. All bulk solvents were kept over sodium and 4 Å molecular sieves (Acros Organics) prior to use. Benzene-d_6 and THF-d_8 (Cambridge Isotope Laboratories) were dried over a potassium mirror, sublimed, and degassed using freeze-pump-thaw cycles prior to use. Celite and 4 Å molecular sieves were dried under vacuum overnight at 200 °C. $VCl_3(THF)_3$,[1] $NbCl_4(THF)_2$,[2] Li(PNP),[3] and Na/NaCl[4] were prepared according to literature procedures. All other chemicals were purchased from commercial sources and used as received. ^{1}H and ^{31}P NMR spectra were recorded on a Bruker AV-II 500 MHz or AVIII 400 MHz spectrometers. ^{13}C, HSQC, COSY, and HMQC NMR spectra were recorded on a Bruker AV-II 500 MHz spectrometer. ^{1}H, ^{13}C NMR chemical shifts are reported and referenced to the internal residual proton or carbon resonances of C_6D_6 (δ 7.2 or 128.1). ^{31}P NMR chemical shifts are reported with respect to external H_3PO_4 (δ 0.0). $^{125}Te\{^{1}H\}$ NMR spectra were recorded on a AVIII 400 MHz spectrometer and chemical shifts are reported with respect to external $(PhTe)_2$ (δ 420.0). UV-vis spectroscopic measurements were carried out using a J-Young valve 1 cm quartz cell on a Cary 5000 Spectrometer. Elemental analyses were performed by Midwest Microlab, Inc (Indiana, USA). Experimental details on X-ray Crystallography and X-ray Absorption Spectroscopy (XAS) can be found under their respective sections within this document.

Statement on Elemental Analysis

Due to the extreme air-sensitivity of the complexes herein and the EA analysis being performed in a glovebox with sub-optimal O_2 levels, the EA data cannot be presented with significant reliability. Over the years we have sent samples for combustion analysis and have received mixed results with some of these companies stating their conditions for data collection are not under anaerobic conditions. However, thorough characterization by NMR spectroscopy and complete single crystal X-ray diffraction (sc-XRD) data provides confirmation of all the compounds reported herein. We refer the reader to an interesting discussion on the controversy behind EA.[5]

Synthesis of $Li(THF)TeCH_2SiMe_3$ (1) and $Li(12\text{-crown-}4)_2TeCH_2SiMe_3$ (2)

A pentane (6 mL) solution of $LiCH_2SiMe_3$ (50 mg, 531 μmol) and a THF (4 mL) suspension of Te (67.6 mg, 531 μmol) was cooled to -35 °C for 45 minutes. The Te suspension was then added dropwise to the pentane solution of $LiCH_2SiMe_3$. The Te metal slowly reacted within 30 minutes to give a pale yellow

suspension (NOTE: if the solvent is not cooled to -35 °C completely or if the Te is added too quickly, the reaction gives way to a red solution with decomposed and oxidized compounds instead of a pale yellow suspension). The reaction was stirred for an hour and the solvent was removed under vacuum leaving behind a pale yellow oil (NOTE: NMR spectra of this oil in C_6D_6 show pure product formation, $Li(THF)TeCH_2SiMe_3$ (**1**), and this can be used in-situ without further purification). The reaction was re-dissolved in toluene and 12-crown-4 (86 µL) was added at -35 °C and stirred for 30 minutes. The solvent was removed under vacuum leaving behind a white solid. THF (6 mL) was added to the solid and filtered through Celite. Concentration of the filtrate to ~ 3 mL and the addition of drops of pentane followed by storage in a -35 °C freezer gave needle-shaped pale crystals of **2** (238 mg, 414 µmol, 78%).

$Li(THF)TeCH_2SiMe_3$ (1): ^{1}H NMR (400 MHz, Benzene-*d*$_6$, 300 K) δ 3.92 (d, *J* = 6.3 Hz, 4H. THF), 1.57 (s, 2H, $LiTeCH_2Si(CH_3)_3$), 1.54 – 1.43 (m, 4H, THF), 0.38 (s, 9H, $LiTeCH_2Si(CH_3)_3$). **^{13}C NMR (126 MHz, Benzene-*d*$_6$, 300 K)** δ 69.22 (THF), 25.44 (THF), -0.04 ($LiTeCH_2Si(CH_3)_3$), -13.36 ($LiTeCH_2Si(CH_3)_3$). **^{29}Si NMR (79 MHz, Benzene-*d*$_6$, 300 K)** δ 2.28. **^{7}Li NMR (155 MHz, Benzene-*d*$_6$)** δ 2.43. **^{125}Te{^{1}H} NMR (126 MHz, Benzene-*d*$_6$, 300 K)** δ -1789.59.

$Li(12\text{-}c\text{-}4)_2TeCH_2SiMe_3$ (2): ^{1}H NMR (400 MHz, THF-d$_8$, 300 K) δ 3.75 (32 H, 12-crown-4), 0.95 (2H, $LiTeCH_2Si(CH_3)_3$), 0.02 (9H, $LiTeCH_2Si(CH_3)_3$). **^{13}C NMR (126 MHz, THF-d$_8$, 300 K)** δ 70.68 (12-crown-4), 0.52 ($LiTeCH_2Si(CH_3)_3$), -45.67 ($LiTeCH_2Si(CH_3)_3$). **^{7}Li NMR (155 MHz, THF-d$_8$, 300 K)** δ 0.47. **^{29}Si NMR (79 MHz, THF-d$_8$, 300 K)** δ -0.37. **^{125}Te{^{1}H} NMR (126 MHz, THF-d$_8$, 300 K)** δ -569.42.

Synthesis of $(PNP)Nb(Te)_2$ (3)

Method 1:

To a suspension of $NbCl_4(THF)_2$ (35 mg, 92.4 µmol) in toluene (4 mL), Li(PNP) (40.2 mg, 92.4 µmol) in toluene (3 mL) was added at room temperature and the reaction was allowed to stir for 16 hours resulting in a purple suspension containing $(PNP)NbCl_3$.[6] Complex **1** (Te (35.2 mg, 277 µmol); $LiCH_2SiMe_3$ (26.1 mg, 277 µmol)) was added dropwise to $(PNP)NbCl_3$ at -35 °C. The reaction color became darker and eventually dark brown within 40 minutes. To this reaction, Te (17.6 mg,139 µmol) and drops of PMe_3 were added. The reaction was stirred for 16 hours at room temperature. The solvent was removed under vacuum to give a dark solid which was washed with pentane. The remaining solid was dissolved in THF (8 mL), filtered through Celite, and concentrated to ~4 mL. Few drops of pentane were added to the concentrated solution and then stored in a -35 °C freezer. After two days, small green needle-shaped crystals deposited in the bottom of the vial. Decantation followed by pentane washes (3×1 mL) of the crystals yielded 29 mg of **3** (1st crop; 40%). Further concentration and storage of the mother liquor gave more crystals of **3** (2nd

crop; 12 mg; 17%) which were washed with pentane (3×1 mL). This gives a total of 42 mg and 57% total yield.

Method 2:

To a suspension of $NbCl_4(THF)_2$ (35 mg, 92.4 μmol) in toluene (4 mL), Li(PNP) (40.2 mg, 92.4 μmol) in toluene (3 mL) was added at room temperature and the reaction was allowed to stir for 16 hours giving way to a purple suspension containing (PNP)$NbCl_3$.[6] Complex **1** (Te (23.5 mg, 185 μmol); $LiCH_2SiMe_3$ (17.4 mg, 185 μmol)) was added dropwise to (PNP)$NbCl_3$ at -35 °C resulting in a gradual color change to dark purple and finally brown. Then, Na/NaCl (49.2 mg, 102 μmol), Te (17.6 mg, 139 μmol), and drops of PMe_3 were added at room temperature and the reaction was allowed to stir for two days. The solvent was removed under vacuum and the resulting solid was redissolved in THF (8 mL) and filtered through Celite. Concentration of the solvent to ~3 mL followed by the addition of drops of pentane and storage at -35 °C freezer yielded **3** as green needles in the bottom of the vial (18 mg, 25%). The mother liquor was decanted and the crystals were washed with pentane (3×1 mL). Further concentration and storage at -35 °C gave a second crop of crystals (8 mg, 11%). A total of 26 mg of product **3** was obtained (36% yield).

^{1}H NMR (500 MHz, Benzene-*d*$_6$, 300 K) δ 7.12 (d, $^2J_{HH}$ = 8.6 Hz, 2H, PNPAr), 6.83 (d, $^2J_{HH}$ = 8.4 Hz, 2H, PNPAr), 6.70 (s, 2H, PNPAr), 2.05 (s, 6H, PNP tolyl CH_3), 1.98 – 1.89 (m, 4H, PCH(CH_3)$_2$), 1.40 (dd, $^3J_{HP}$ = 14.6, $^3J_{HH}$ = 5.3 Hz, 6H, PCH(CH_3)$_2$), 1.08 (dd, $^3J_{HP}$ = 16.7, $^3J_{HH}$ = 7.2 Hz, 6H, PCH(CH_3)$_2$), 0.94 (dd, $^3J_{HP}$ = 15.7, $^3J_{HH}$ = 6.2 Hz, 6H, PCH(CH_3)$_2$), 0.82 (dd, $^3J_{HP}$ = 11.2, $^3J_{HH}$ = 1.5 Hz, 6H, PCH(CH_3)$_2$). **^{13}C NMR (126 MHz, Benzene-*d*$_6$, 300 K)** δ 159.54 (PNPAr), 133.22 (PNPAr), 132.37 (PNPAr), 128.88 (PNPAr), 118.56 (PNPAr), 117.33 (PNPAr), 30.24 (t, $^2J_{CP}$ = 9.4 Hz, P*C*H(CH_3)$_2$), 20.68 (PNP tolyl *C*H_3), 20.4 (PCH(*C*H_3)$_2$), 20.3 (PCH(*C*H_3)$_2$), 17.99 (PCH(*C*H_3)$_2$), 16.53 (PCH(*C*H_3)$_2$), 14.49 (t, $^2J_{CP}$ = 10.8 Hz, P*C*H(CH_3)$_2$). **^{31}P NMR (162 MHz, Benzene-*d*$_6$, 300 K)** δ 91.44. **^{125}Te{^{1}H} NMR (127 MHz, THF-d$_8$, 300 K)** δ 3896.14 (br, $\Delta\nu_{1/2}$ = 404 Hz). **UV-Vis (THF, λ_{max}/ε nm (ε/M^{-1}cm^{-1}))**: 240 (25143), 261 (25500), 325 (13625), 387 (6893), 469 (2969), 574 (1860). Multiple attempts to obtain satisfactory elemental analysis failed.

Synthesis of (PNP)Ta(Te)$_2$ (4)

TaF_5 (25 mg, 91 μmol) and Li(PNP) (39.6 mg, 91 μmol) were dissolved in Et_2O (in two separate vials) and placed in a -35C freezer. After 30 minutes, Li(PNP) solution was added dropwise to the TaF_5 solution. The solution turned orange with precipitates over time.[7] Complex **1** (Te (46 mg, 364 μmol); $LiCH_2SiMe_3$ (34 mg, 364 μmol)) was then added slowly to the orange suspension containing (PNP)TaF_4, and the reaction was stirred for 16 hours. Solventwas removed under vacuum and the reddish brown solid was washed with pentane until all the pentane washings were colorless (4×2 mL). The remaining solid was dissolved in THF

(10 mL), filtered through Celite and concentrated to ~ 4 mL. Addition of drops of pentane and storage at -35 ^{o}C freezer for two days gave **4** as needle-shaped red crystals (46 mg, 68% yield). The mother liquor was decanted and the crystals were washed with pentane (4×2 mL).

^{1}H NMR (500 MHz, Benzene-*d_6*, 300 K) δ 7.06 (d, $^{2}J_{HH}$ = 8.3 Hz, 2H, PNPAr), 6.84 (d, $^{2}J_{HH}$ = 6.3 Hz, 2H, PNPAr), 6.69 (s, 2H, PNPAr), 2.24 – 2.13 (m, 2H, PC*H*(CH_3)$_2$), 2.13 – 2.04 (m, 2H, PC*H*(CH_3)$_2$), 2.02 (s, 6H, PNP tolyl CH_3), 1.40 (dd, $^{3}J_{HP}$ = 17.1, $^{3}J_{HH}$ = 7.5 Hz, 6H, PCH(C*H*$_3$)$_2$), 1.20 (dd, $^{3}J_{HP}$ = 17.4, $^{3}J_{HH}$ = 6.8 Hz, 6H, PCH(C*H*$_3$)$_2$), 1.06 (dd, $^{3}J_{HP}$ = 16.2, $^{3}J_{HH}$ = 7.3 Hz, 6H, PCH(C*H*$_3$)$_2$), 0.83 (dd, $^{3}J_{HP}$ = 12.0, $^{3}J_{HH}$ = 6.9 Hz, 6H, PCH(C*H*$_3$)$_2$). **^{13}C NMR (126 MHz, Benzene-*d_6*, 300 K)** δ 158.72 (t, $^{1}J_{CP}$ = 13.4 Hz, PNPAr), 133.32 (PNPAr), 132.85 (PNPAr), 129.39 (PNPAr), 118.72 (t, $^{2}J_{CP}$ = 4.5 Hz, PNPAr), 117.25 (t, $^{2}J_{CP}$ = 19.2 Hz, PNPAr), 31.30 (t, $^{2}J_{CP}$ = 10.2 Hz, P*C*H(CH_3)$_2$), 20.70 (PCH(*C*H$_3$)$_2$), 20.29 (PNP tolyl *C*H$_3$), 19.90 (PCH(*C*H$_3$)$_2$), 18.08 (PCH(*C*H$_3$)$_2$), 16.95 (PCH(*C*H$_3$)$_2$), 15.13 (t, *J* = 12.0 Hz, P*C*H(CH_3)$_2$). **^{31}P NMR (203 MHz, Benzene-*d_6*, 300 K)** δ 110.92. **$^{125}Te\{^{1}H\}$ NMR (127 MHz, Benzene-*d_6*, 300 K)** δ 3040.89 (br, $\Delta\nu_{1/2}$ = 560 Hz). **UV-Vis (THF, λ_{max}/ε nm (ε/$M^{-1}cm^{-1}$))**: 247 (21054), 282 (11500), 309 (13232), 328 (10179), 354 (8929), 442 (2571) 505 (2110). Multiple attempts to obtain satisfactory elemental analysis failed.

Modified synthesis of (PNP)V(Te)$_2$ (V)

To a suspension of $VCl_3(THF)_3$ (22 mg, 60 µmol) in toluene (4 mL), Li(PNP) (26.1 mg, 92.4 µmol) in toluene (3 mL) was added at room temperature and the reaction was allowed to stir for 16 hours giving way to a maroon suspension containing (PNP)VCl_2.[8] Complex **1** (Te (15.2 mg, 120 µmol) in THF (2 mL) to $LiCH_2SiMe_3$ (11.3 mg, 120 µmol) in pentane (3 mL)) was then added slowly to (PNP)VCl_2 followed by Te metal (11.4, 89 µmol) and drops of PMe_3. The reaction was stirred for two days after which the solvent was removed under vacuum leaving behind a dark solid. The solid was washed with pentane (2×4 mL) where the pentane washes were green. The remaining undissolved material was dissolved in THF and filtered through Celite. The solvent was removed under vacuum and the solid was further washed with pentane (2×4 mL). The solid was dried under vacuum to give pure **V** (32 mg, 43 µmol, 73% yield). ^{1}H and ^{31}P NMR spectra of the solid in C_6D_6 correspond to the published data.[8] **UV-Vis (THF, λ_{max}/ε nm (ε/$M^{-1}cm^{-1}$))**: 234 (18696), 273 (17181), 320 (14929), 423 (3946), 621 (2290).

Melting Points of all three bis(telluride) complexes (**V**, **3**, and **4**) were collected using a sealed capillary tube with <1 mg of samples inside the capillary tube. All three complexes were stable up to 180 °C with little or no deformation in the color or texture of the solid. Our thermometer precluded temperature reading beyond 200 °C.

NMR Spectroscopy

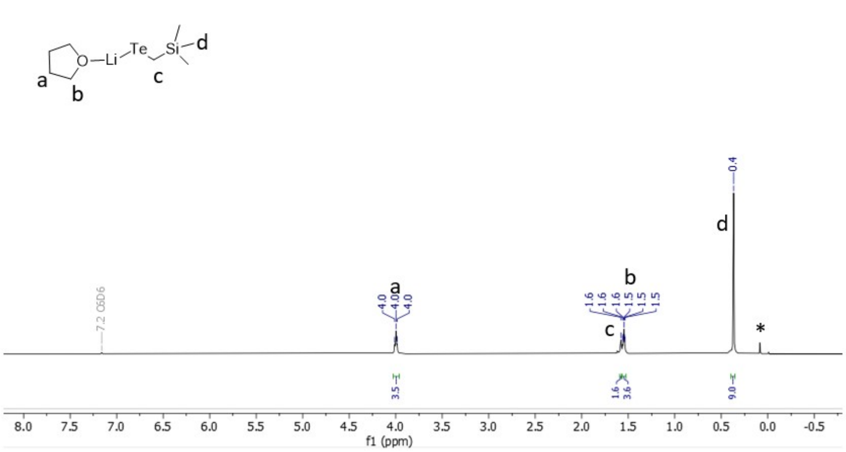

Figure S1. ^{1}H NMR (500 MHz, C_6D_6, 300 K) spectrum of **1**. *Corresponds to silicon grease.

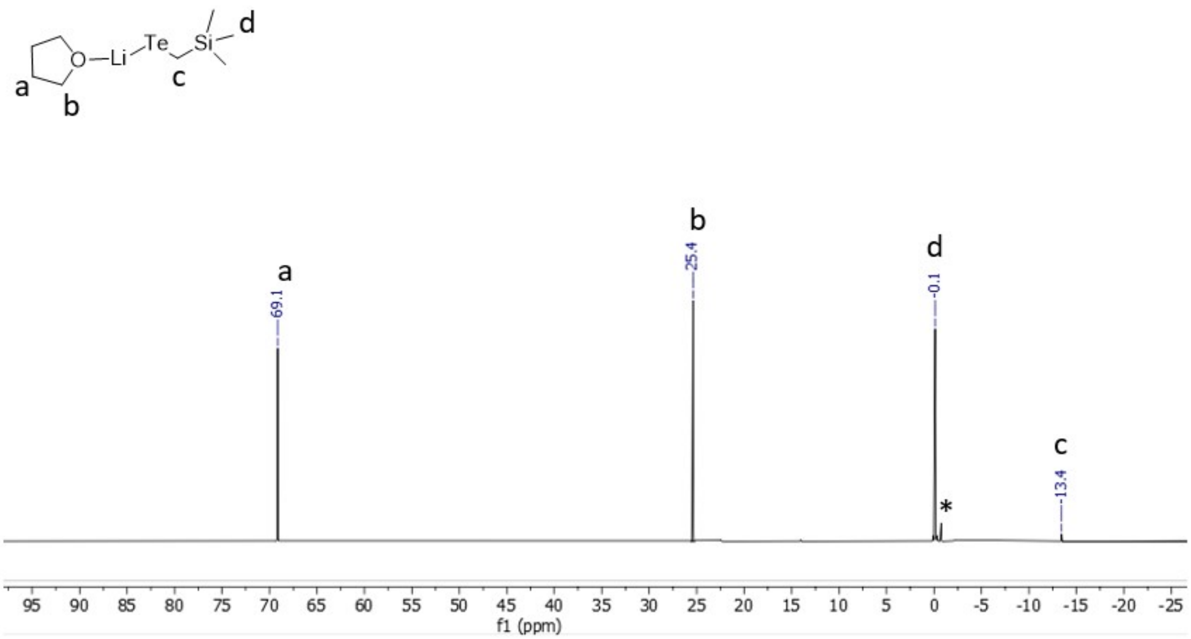

Figure S2. ^{13}C NMR (500 MHz, C_6D_6, 300 K) spectrum of **1**. *Corresponds to unidentified impurity

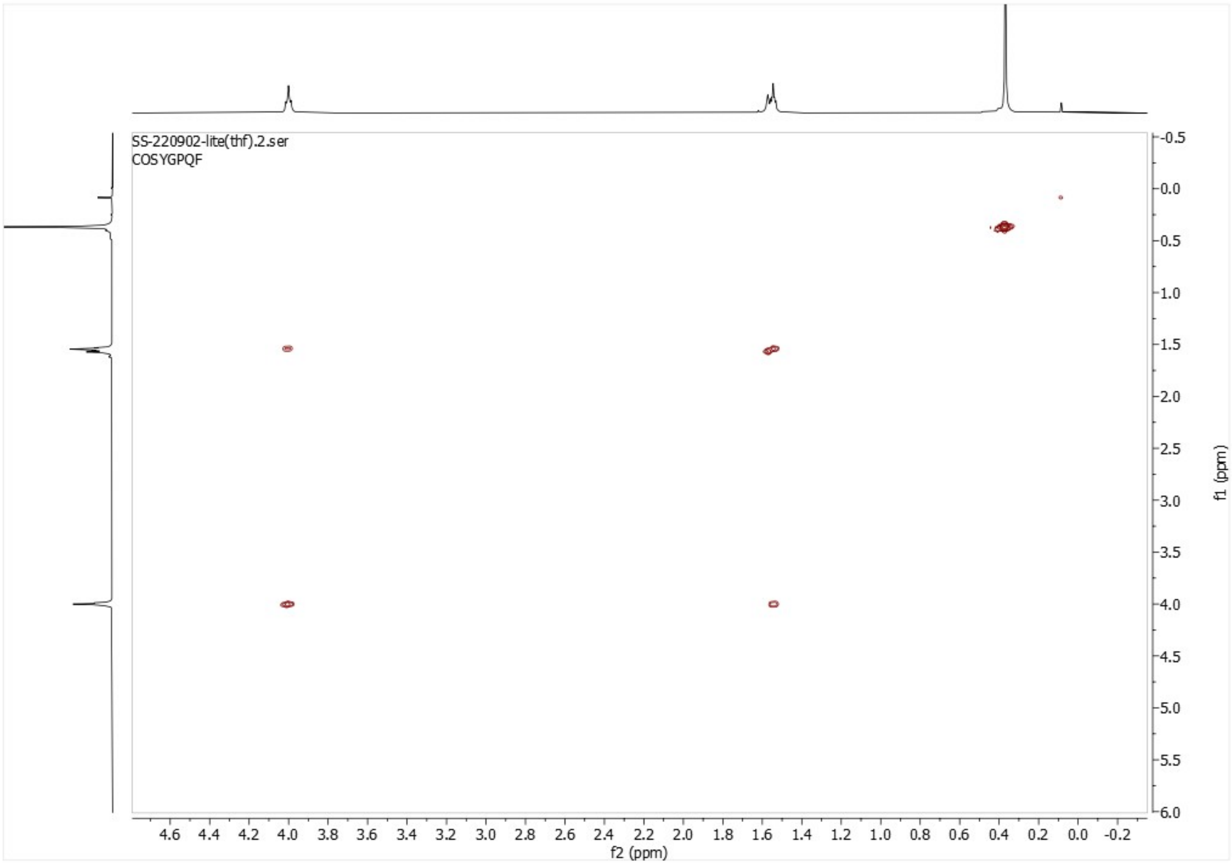

Figure S3. ^1H-^1H COSY NMR (500 MHz, C_6D_6, 300 K) spectrum of **1**.

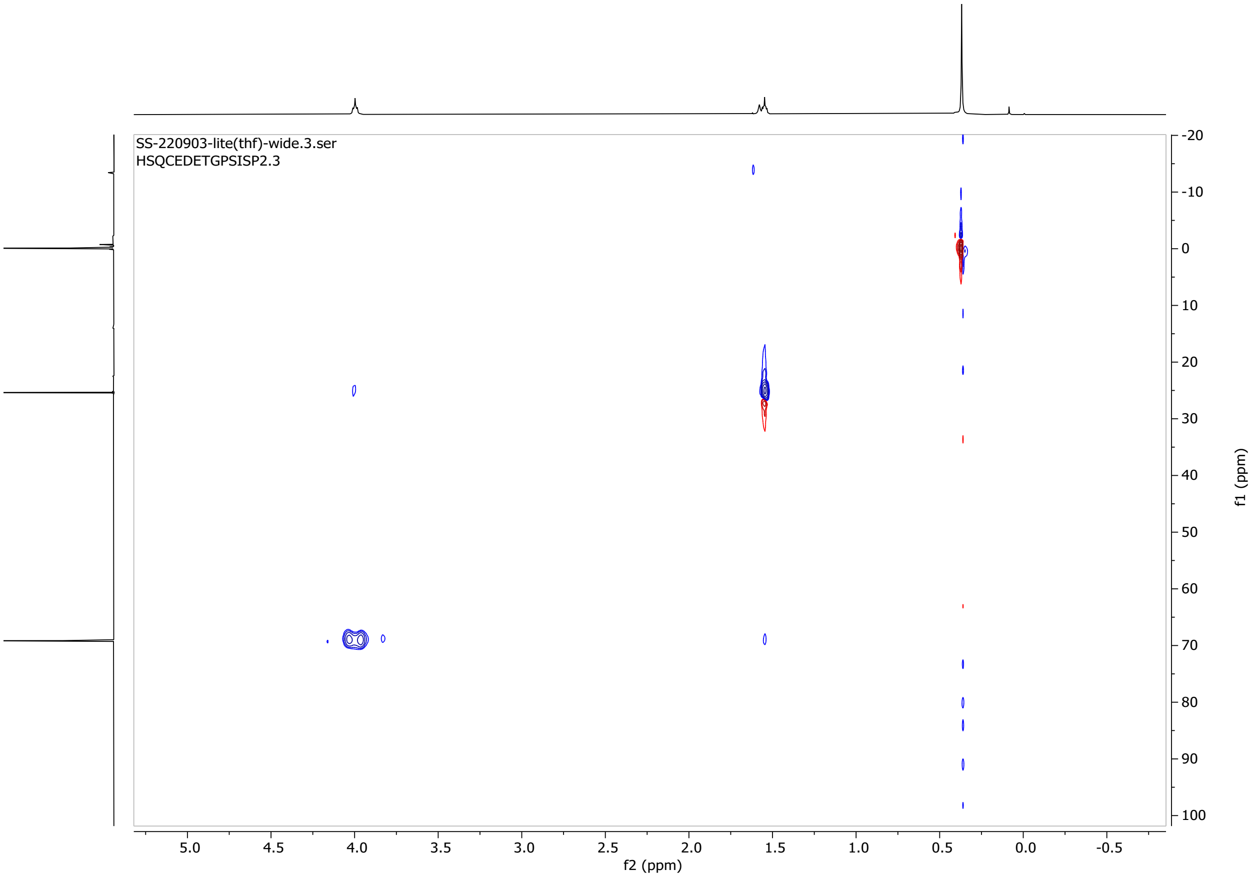

Figure S4. ^1H-^{13}C HSQC NMR (500 MHz, C_6D_6, 300 K) spectrum of **1**.

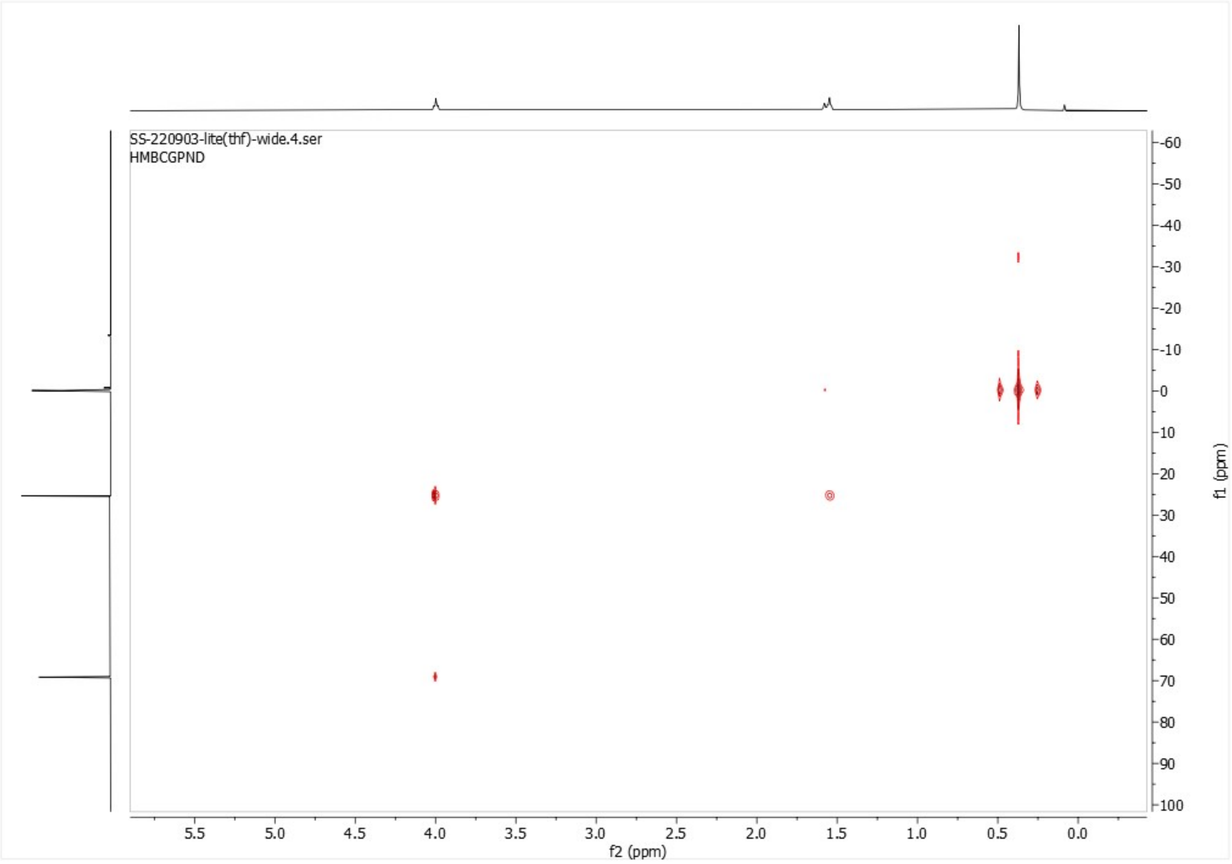

Figure S5. ^{1}H-^{13}C HSQC NMR (500 MHz, C_6D_6, 300 K) spectrum of **1**.

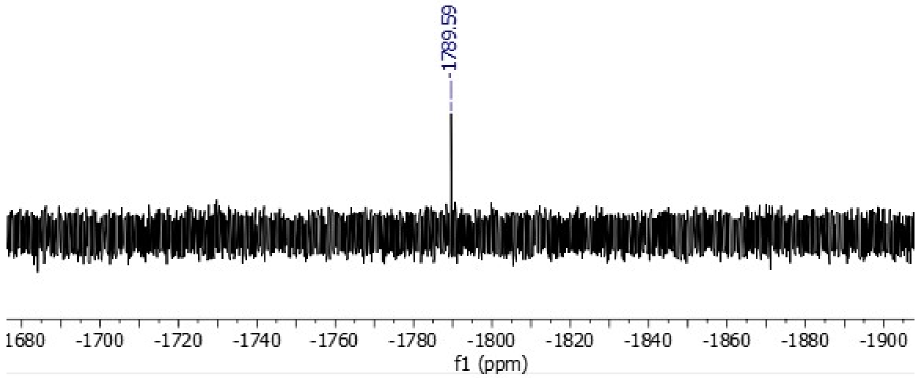

Figure S6. $^{125}Te\{^1H\}$ NMR (126 MHz, C_6D_6, 300 K) spectrum of **1**.

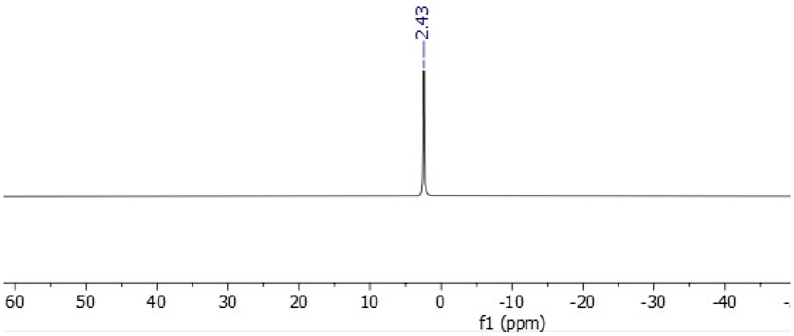

Figure S7. ^7Li NMR (155 MHz, C_6D_6, 300 K) spectrum of **1**.

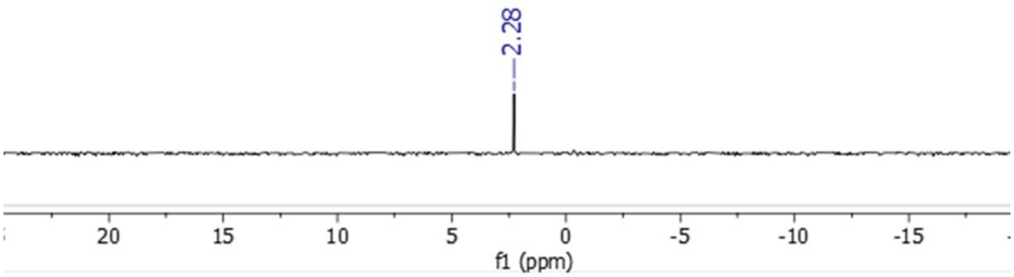

Figure S8. ^{29}Si NMR (79 MHz, C_6D_6, 300 K) spectrum of **1**.

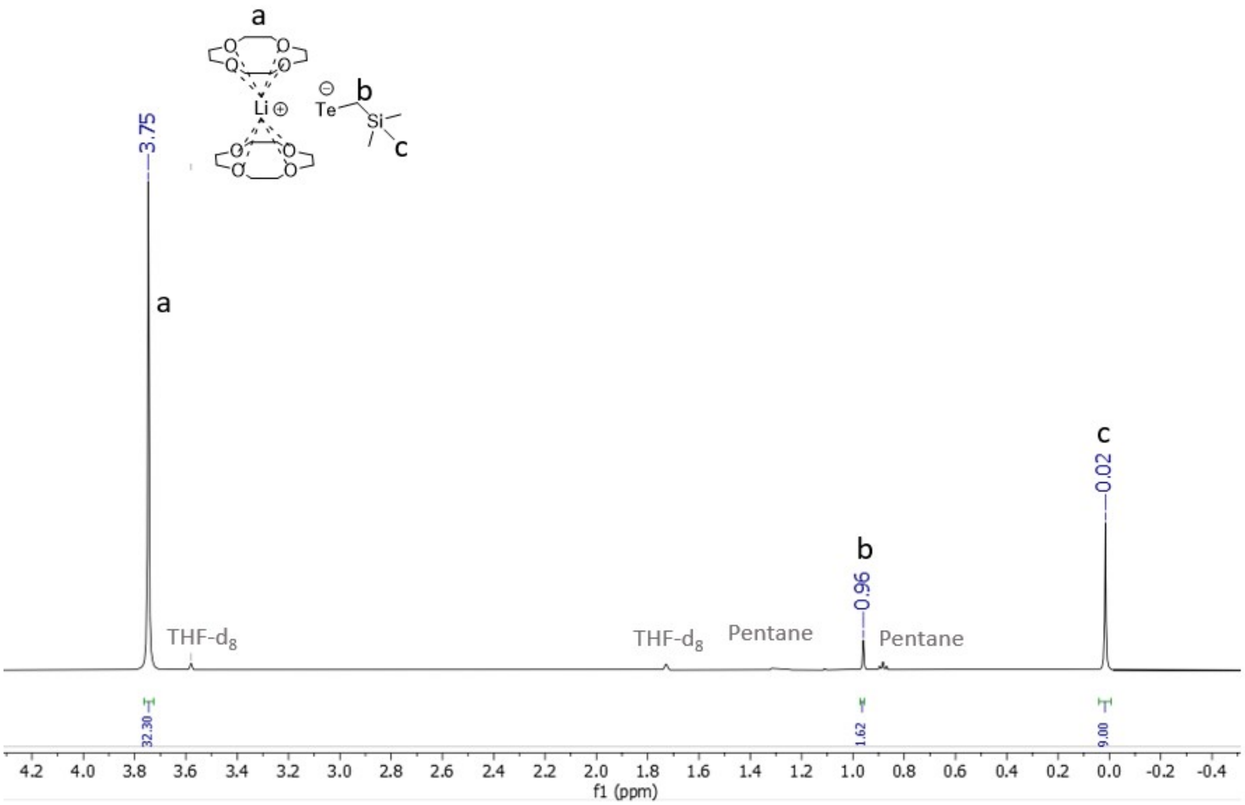

Figure S9. ^{1}H NMR (500 MHz, THF-d_8, 300 K) spectrum of **2**.

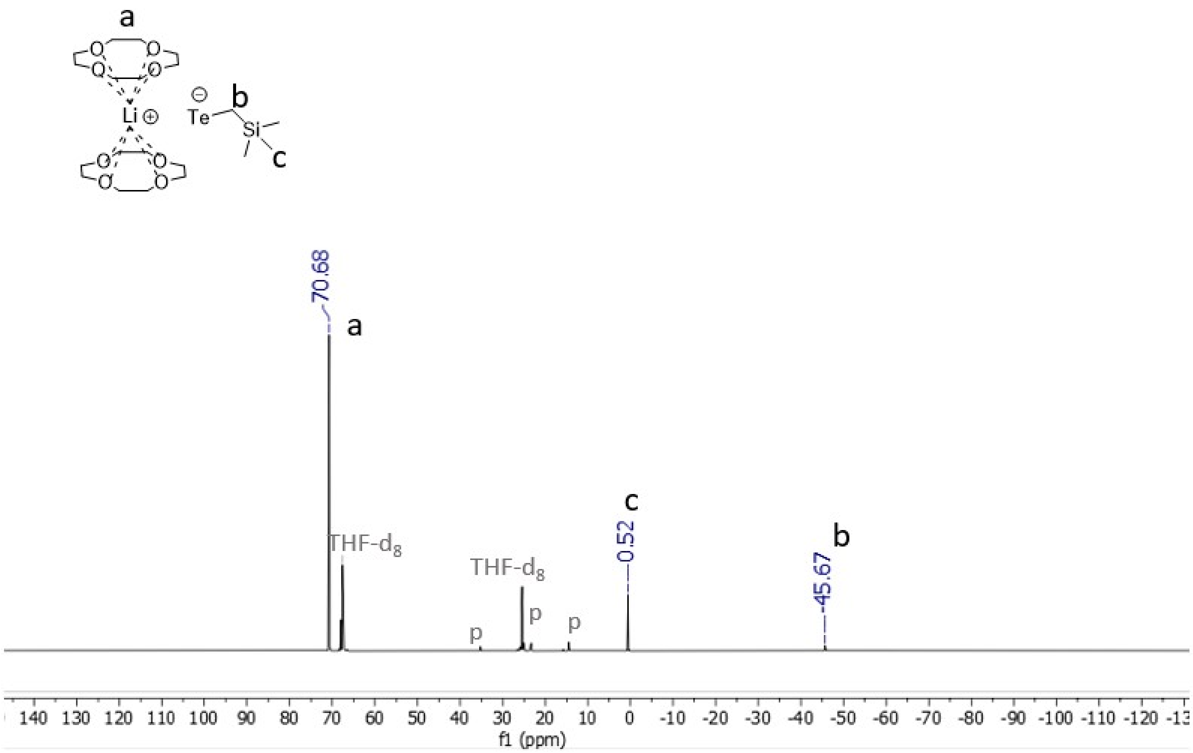

Figure S10. ^{13}C NMR (500 MHz, THF-d_8, 300 K) spectrum of **2**. Residual solvent (pentane) is marked as ‘p’.

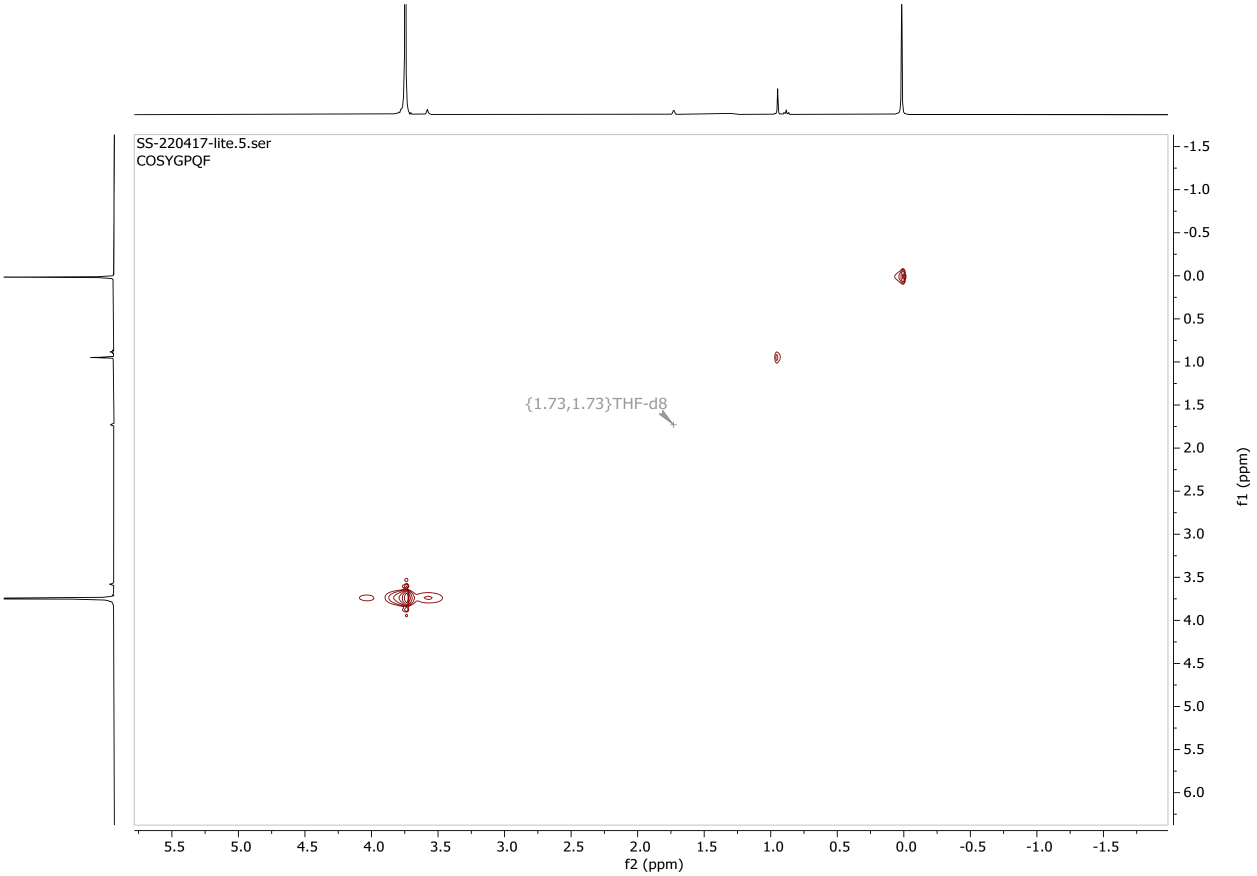

Figure S11. ^{1}H-^{1}H COSY NMR (500 MHz, THF-d_8, 300 K) spectrum of **2**.

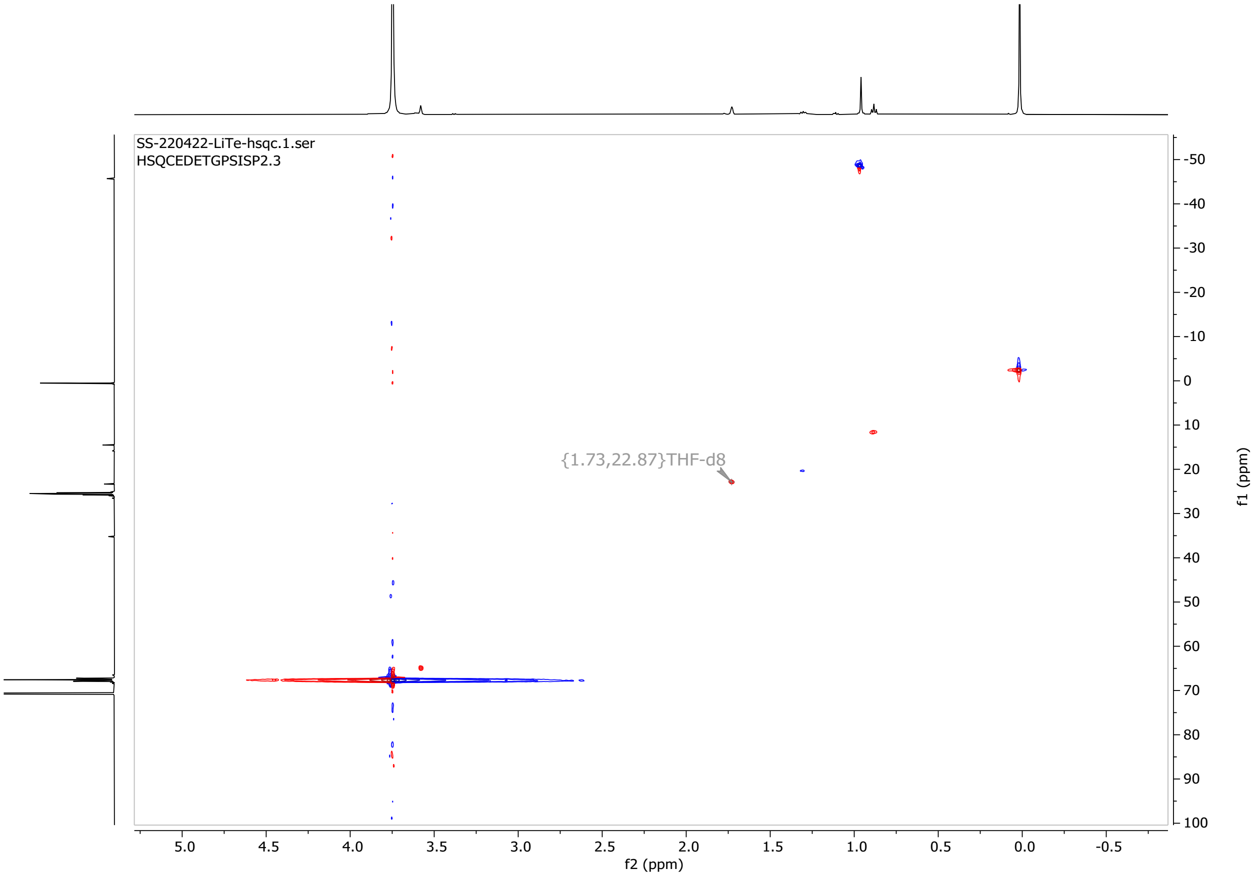

Figure S12. ^{1}H-^{13}C HSQC NMR (500 MHz, THF-d_8, 300 K) spectrum of **2**.

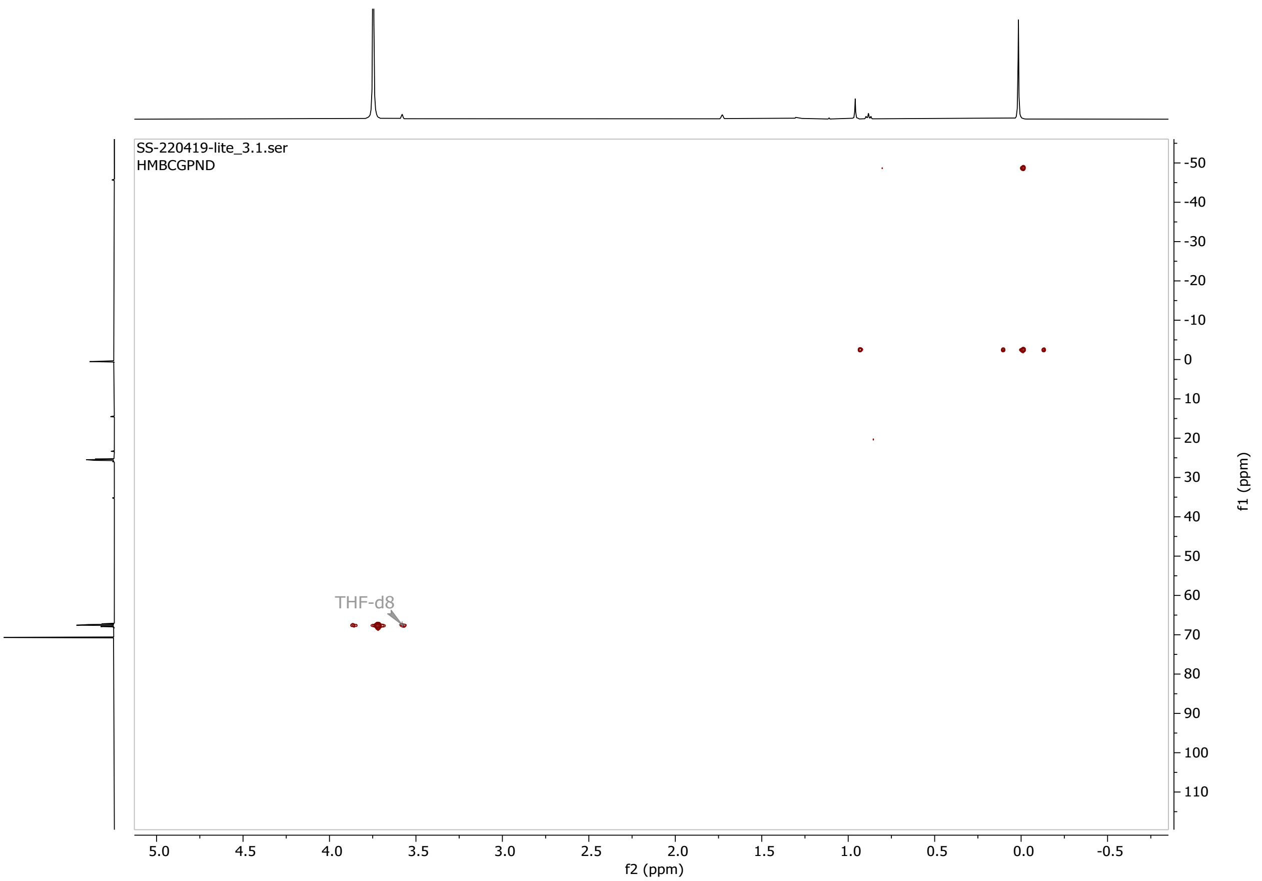

Figure S13. ^{1}H-^{13}C HMBC NMR (500 MHz, THF-d_8, 300 K) spectrum of **2**.

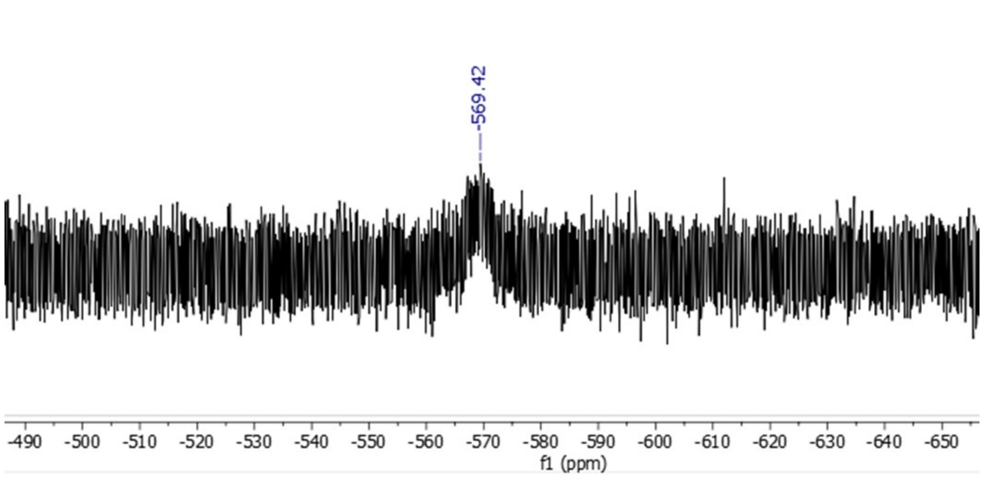

Figure S14. ^{125}Te{^{1}H} NMR (126 MHz, THF-d_8, 300 K) spectrum of **2**.

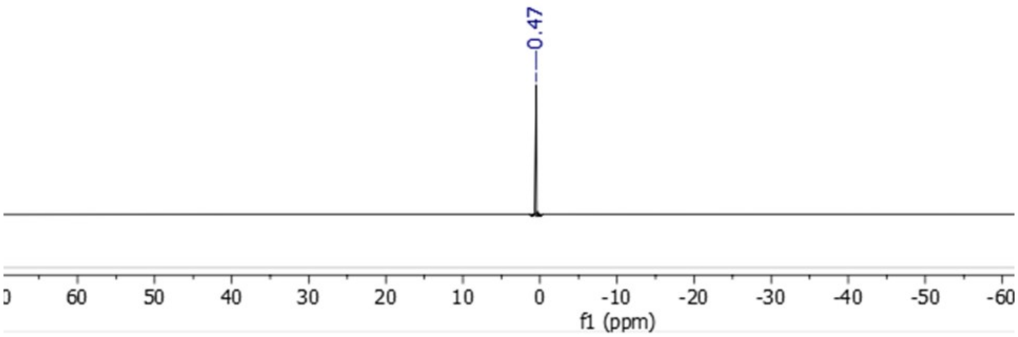

Figure S15. ^{7}Li NMR (155 MHz, THF-d_8, 300 K) spectrum of **2**.

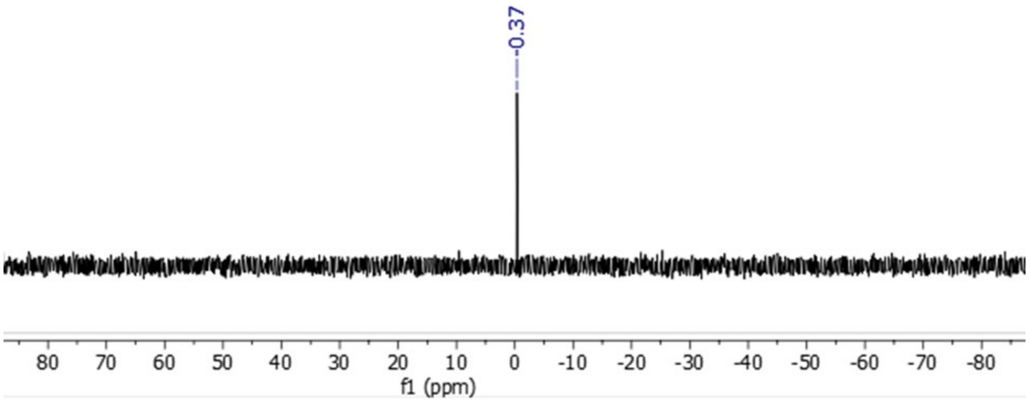

Figure S16. ^{29}Si NMR (79 MHz, THF-d_8, 300 K) spectrum of **2**.

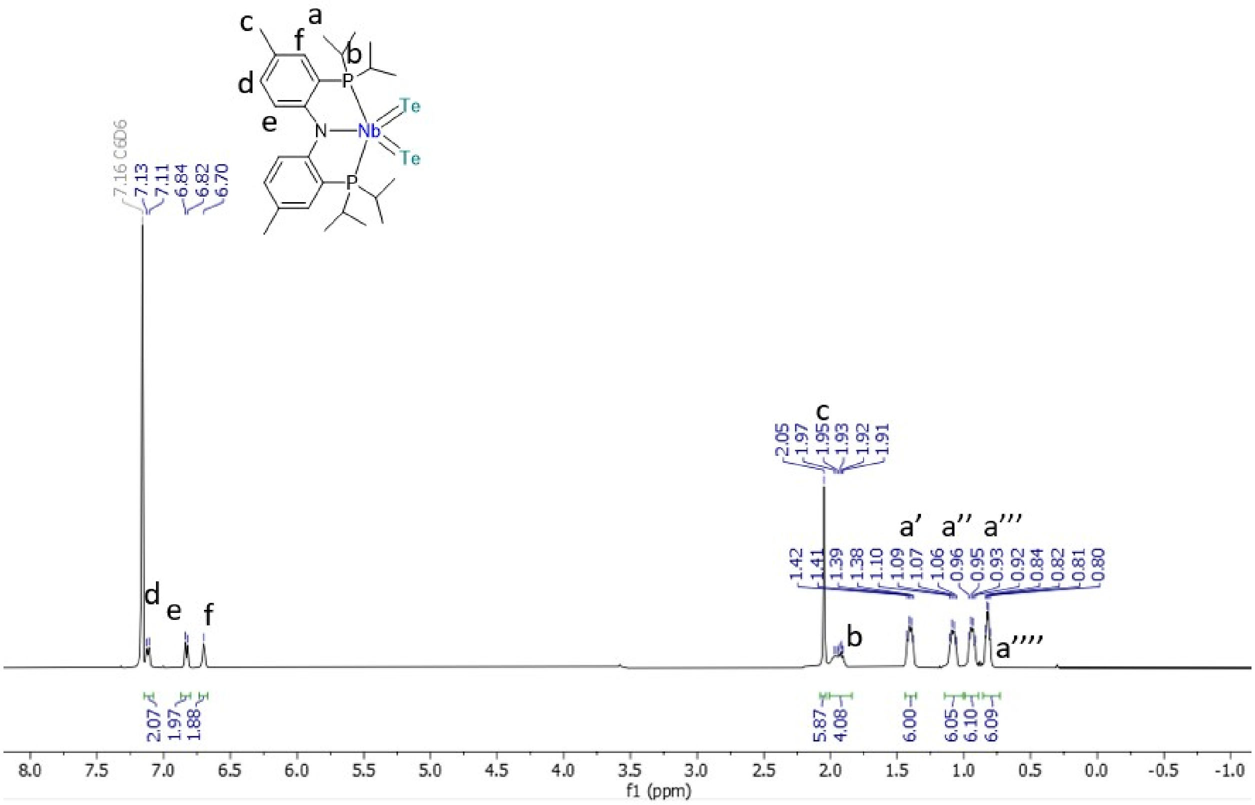

Figure S17. ^{1}H NMR (500 MHz, C_6D_6, 300 K) spectrum of **3**.

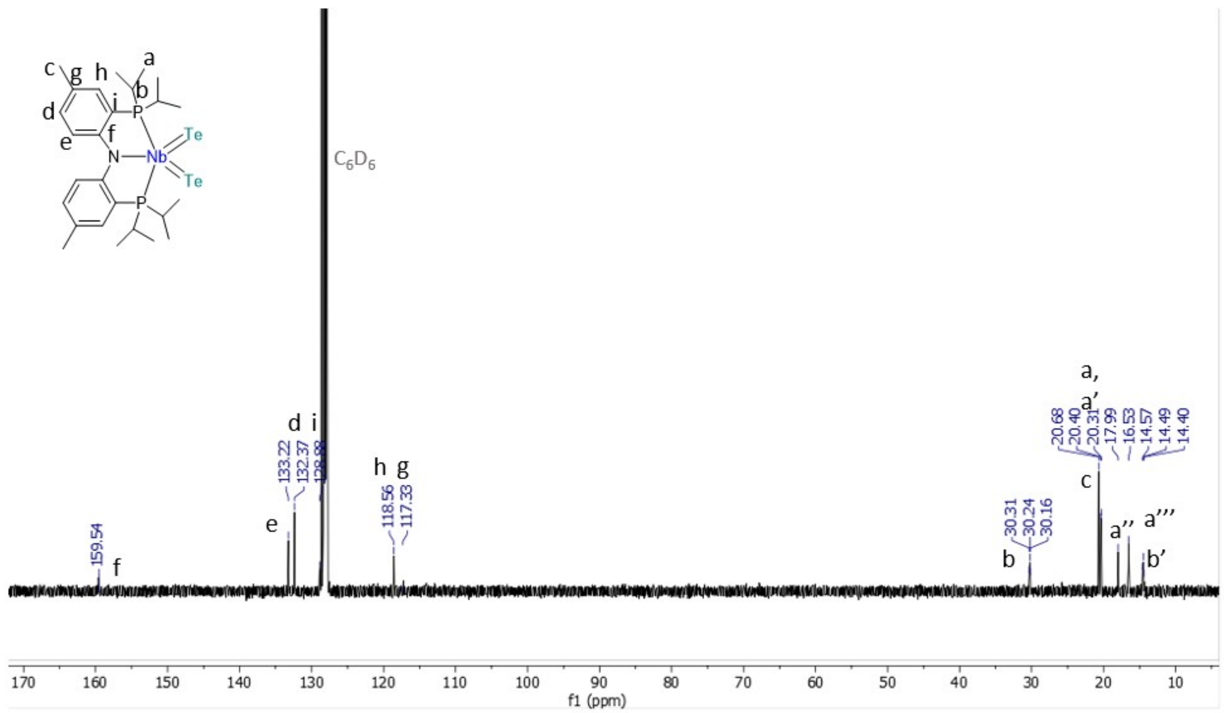

Figure S18. ^{13}C NMR (500 MHz, C_6D_6, 300 K) spectrum of **3**.

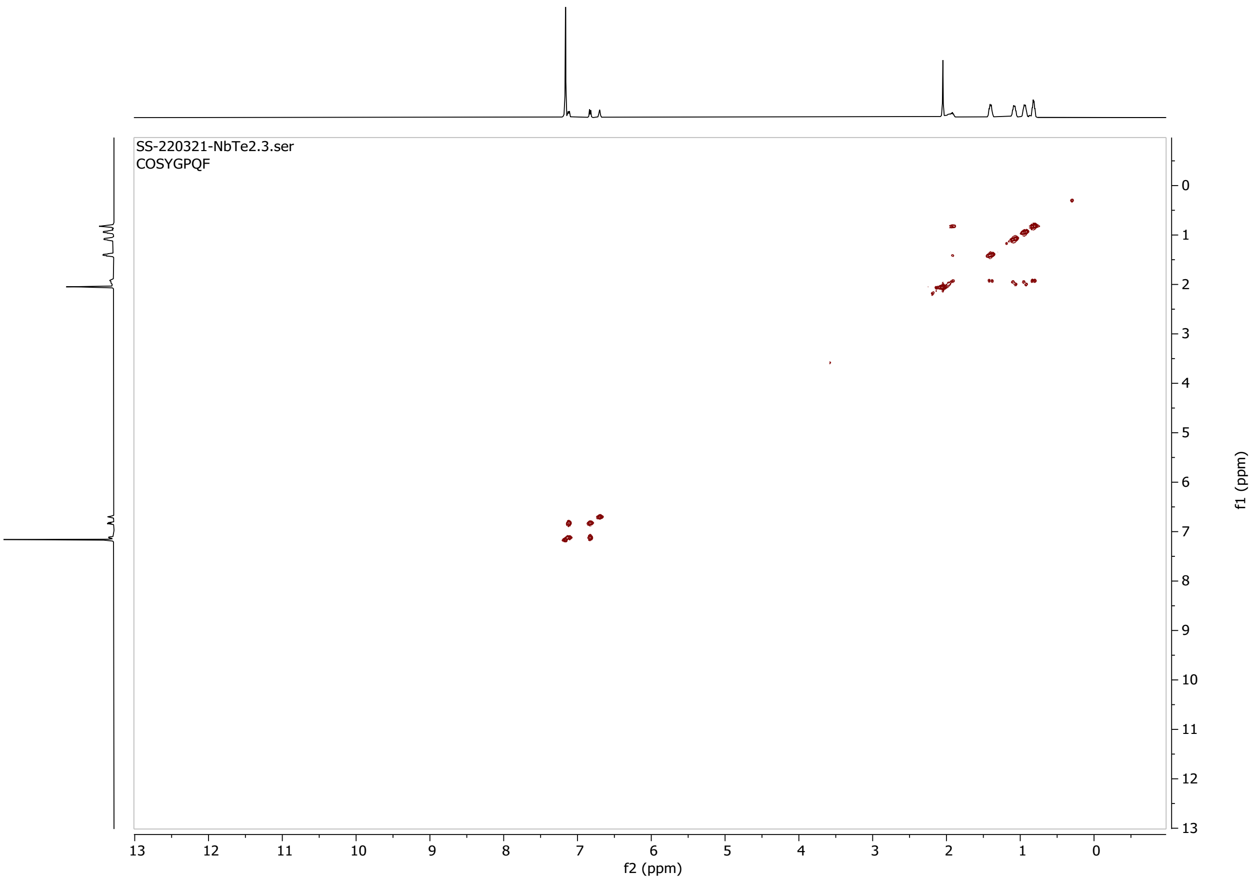

Figure S19. ^{1}H-^{1}H COSY NMR (500 MHz, C_6D_6, 300 K) spectrum of **3**.

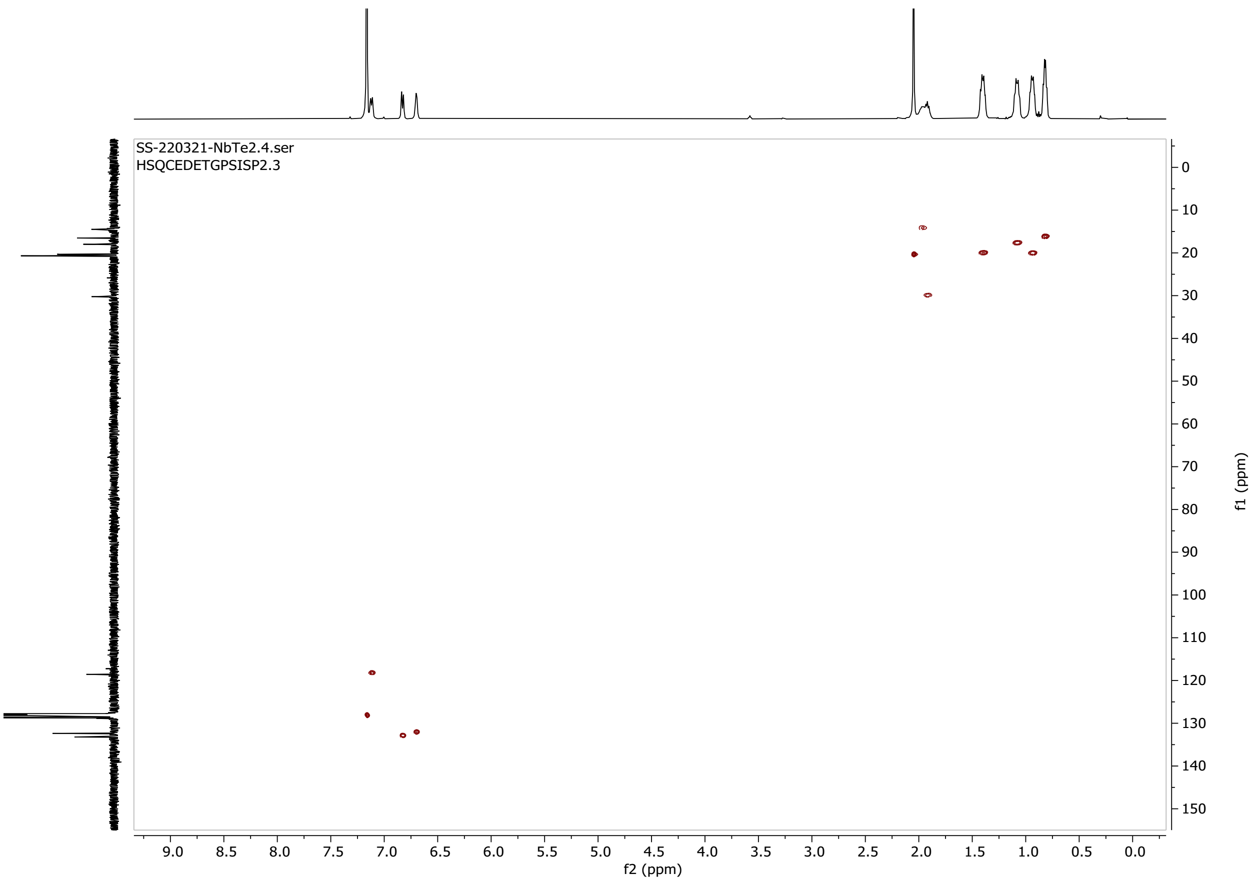

Figure S20. ^{1}H-^{13}C HSQC NMR (500 MHz, C_6D_6, 300 K) spectrum of **3**.

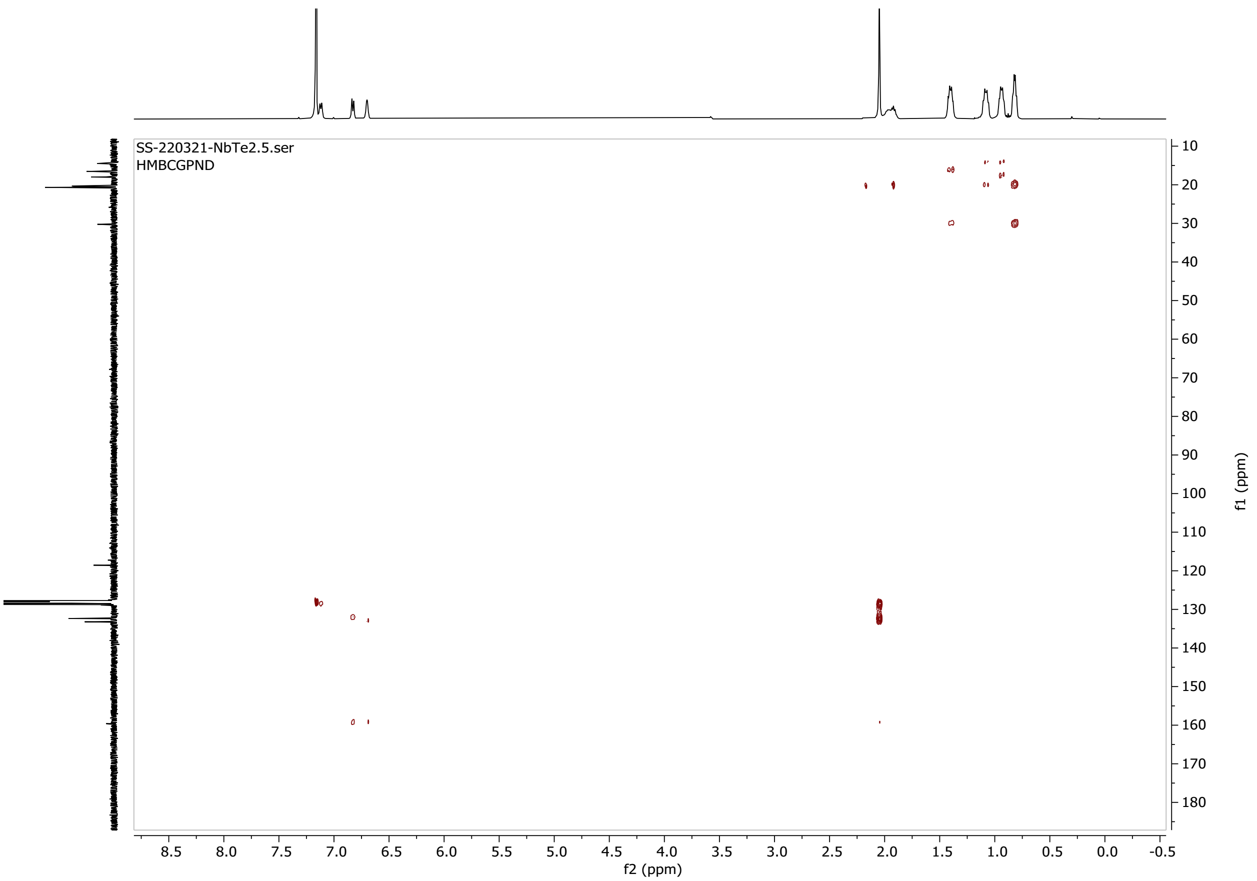

Figure S21. ^{1}H-^{13}C HMBC NMR (500 MHz, C_6D_6, 300 K) spectrum of **3**.

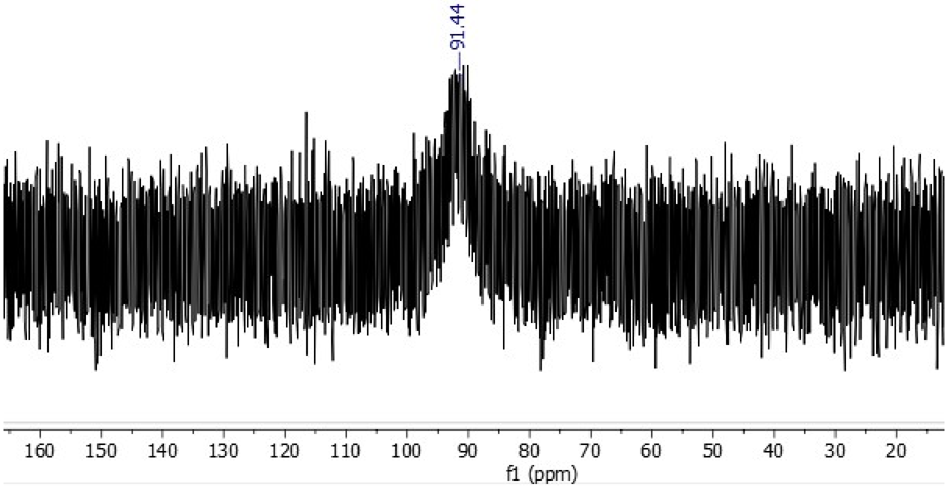

Figure S22. ^{31}P NMR (203 MHz, C_6D_6, 300 K) spectrum of **3**.

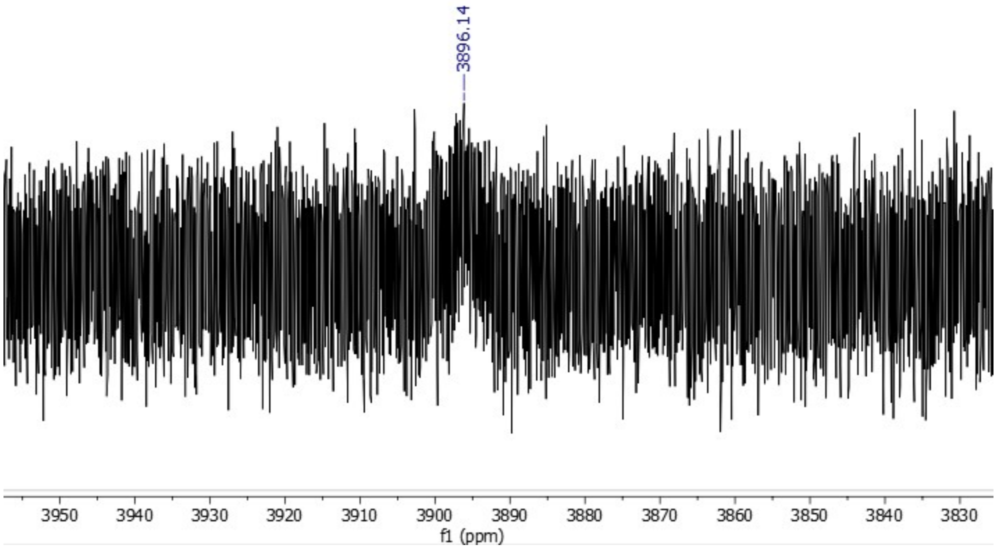

Figure S23. $^{125}Te\{^1H\}$ NMR (126 MHz, C_6D_6, 300 K) spectrum of **3**.

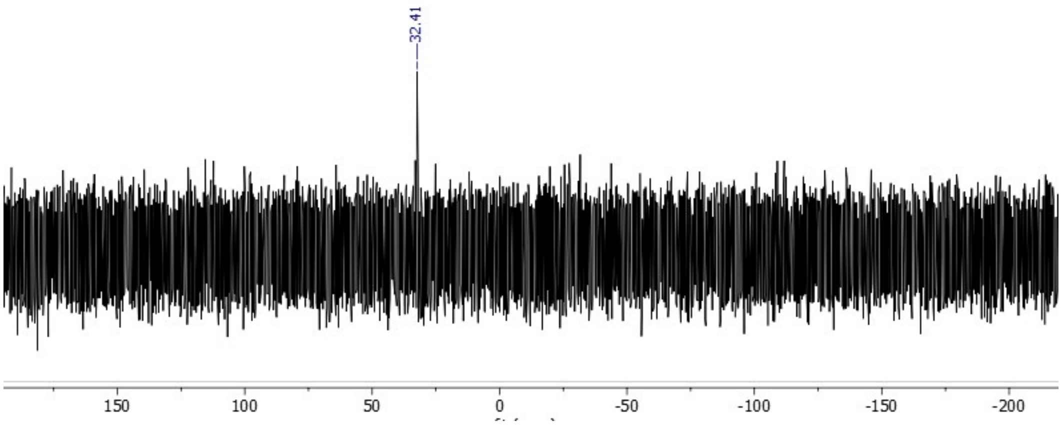

Figure S24. $^{125}Te\{^{1}H\}$ NMR (126 MHz, C_6D_6, 300 K) spectrum of $Te(CH_2(SiMe_3)_2$ from the pentane washings in the synthesis of **3**. NMR shifts for $Te(CH_2SiMe_3)_2$ is reported in the literature.9

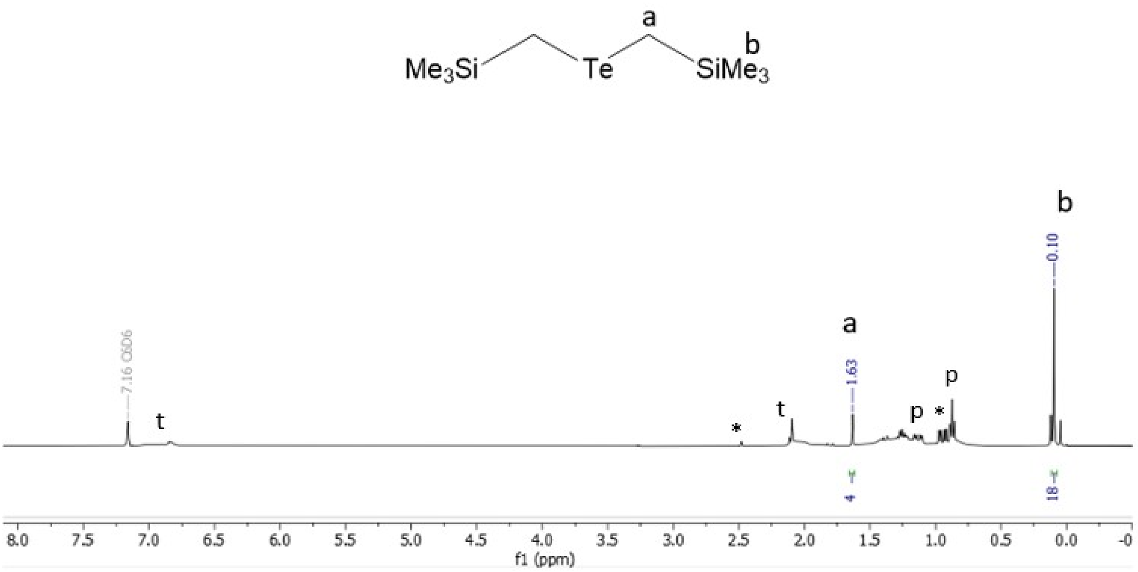

Figure S25. ^{1}H NMR (500 MHz, C_6D_6, 300 K) of pentane washings in the synthesis of **3**. The unlabeled resonances correspond to an impurity that cannot be identified.

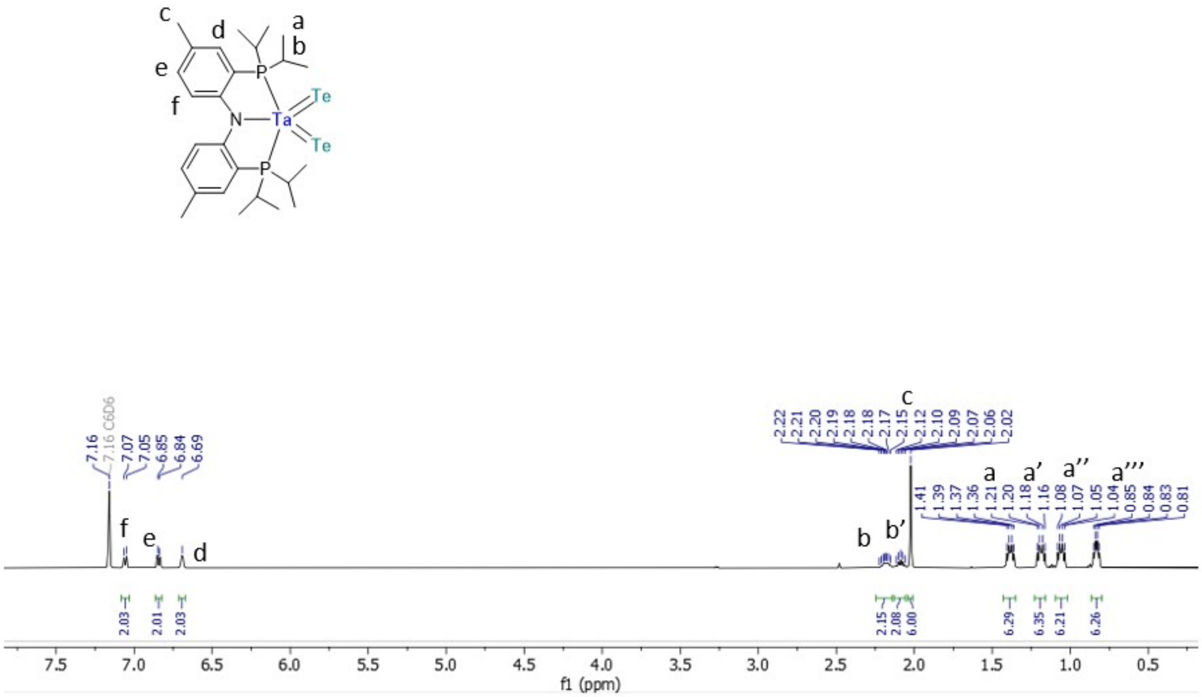

Figure S26. ^{1}H NMR (500 MHz, C_6D_6, 300 K) spectrum of **4**.

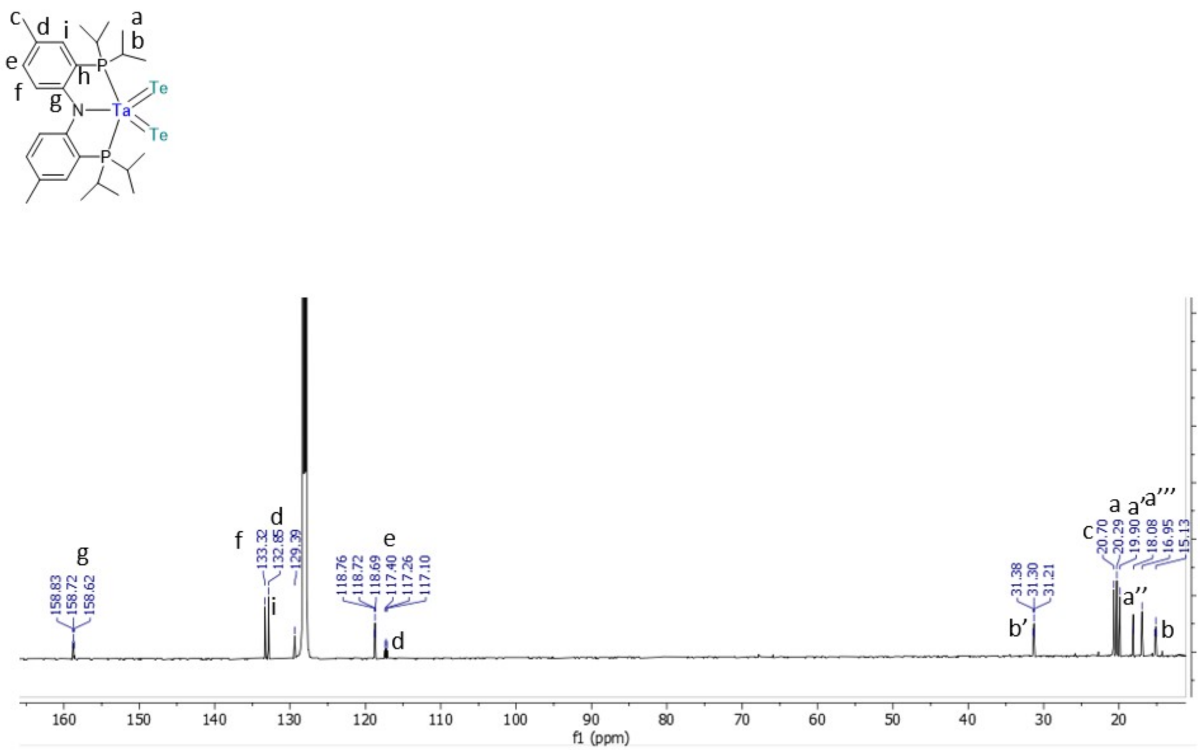

Figure S27. ^{13}C NMR (500 MHz, C_6D_6, 300 K) spectrum of **4**.

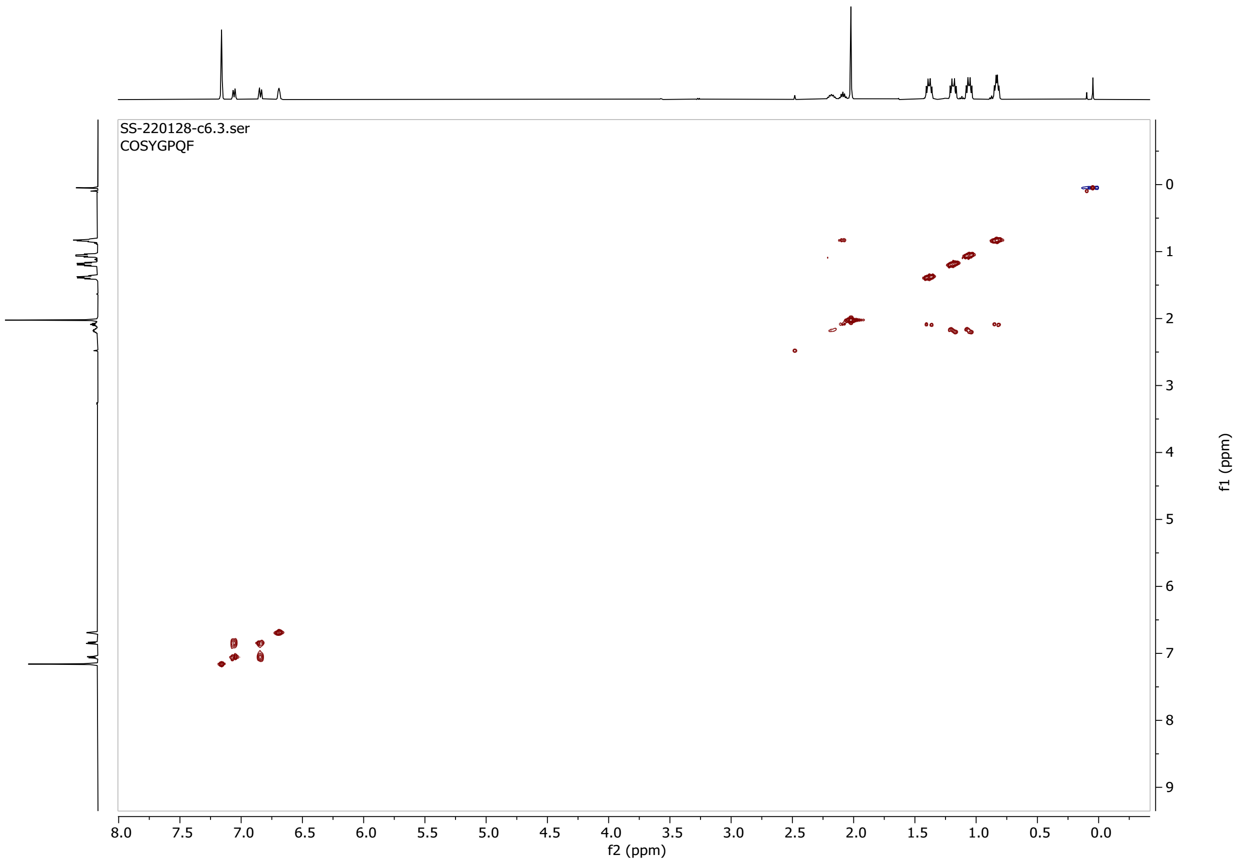

Figure S28. ^{1}H-^{1}H COSY NMR (500 MHz, C_6D_6, 300 K) spectrum of **4**.

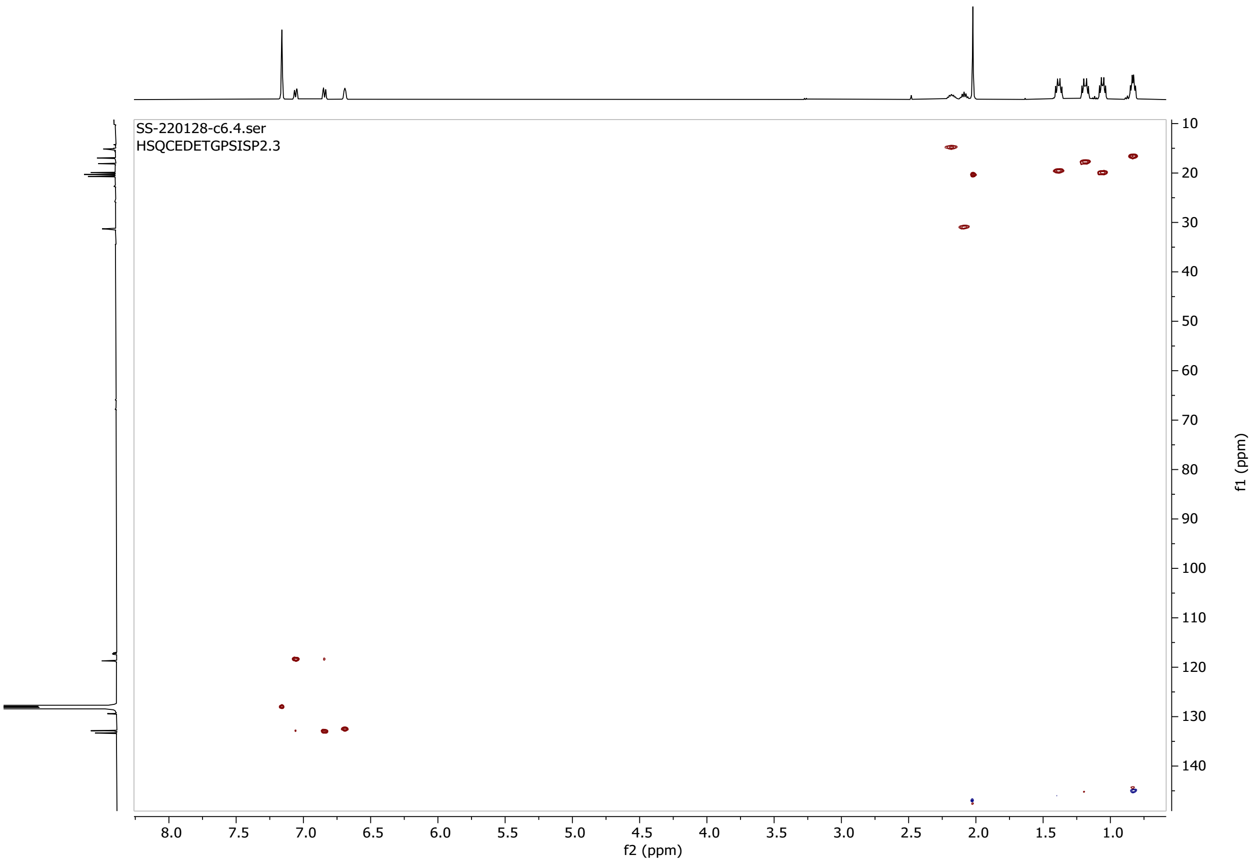

Figure S29. ^{1}H-^{13}C HSQC NMR (500 MHz, C_6D_6, 300 K) spectrum of **4**.

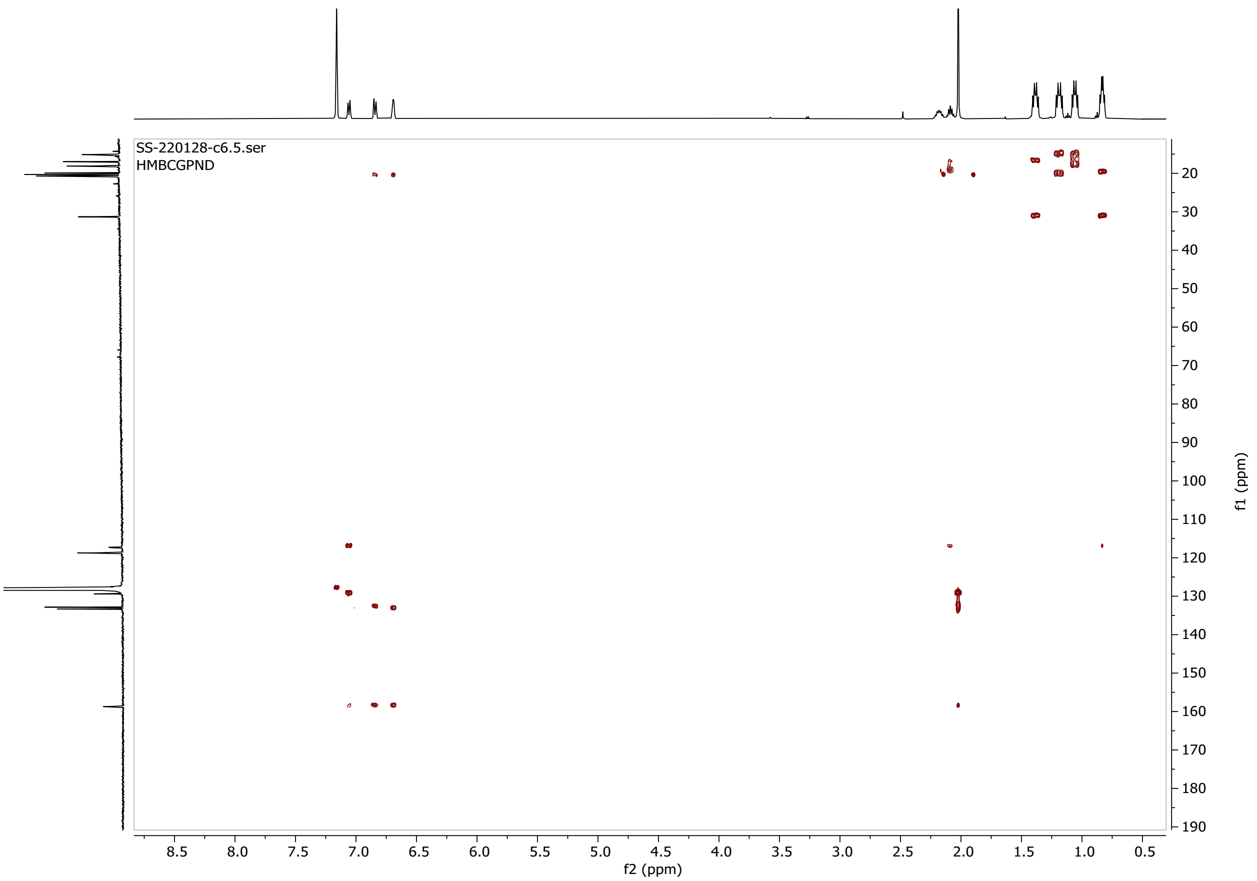

Figure S30. ^1H-^{13}C HMBC NMR (500 MHz, C_6D_6, 300 K) spectrum of **4**.

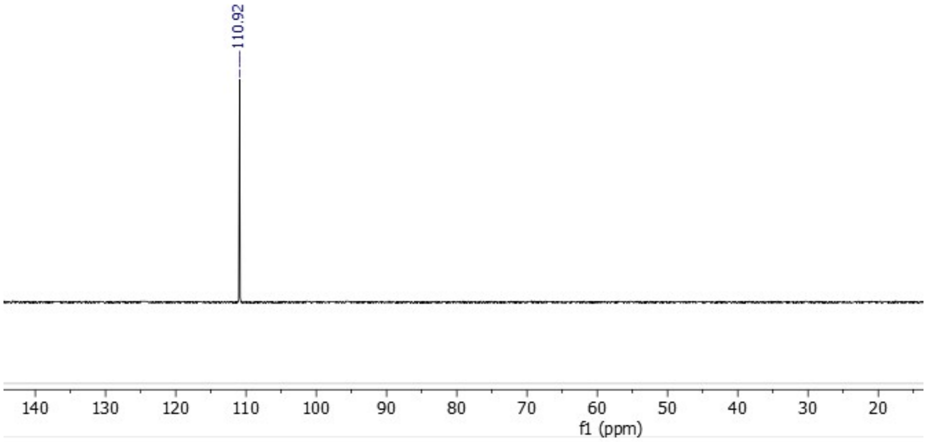

Figure S31. ^{31}P NMR (203 MHz, C_6D_6, 300 K) spectrum of **3**.

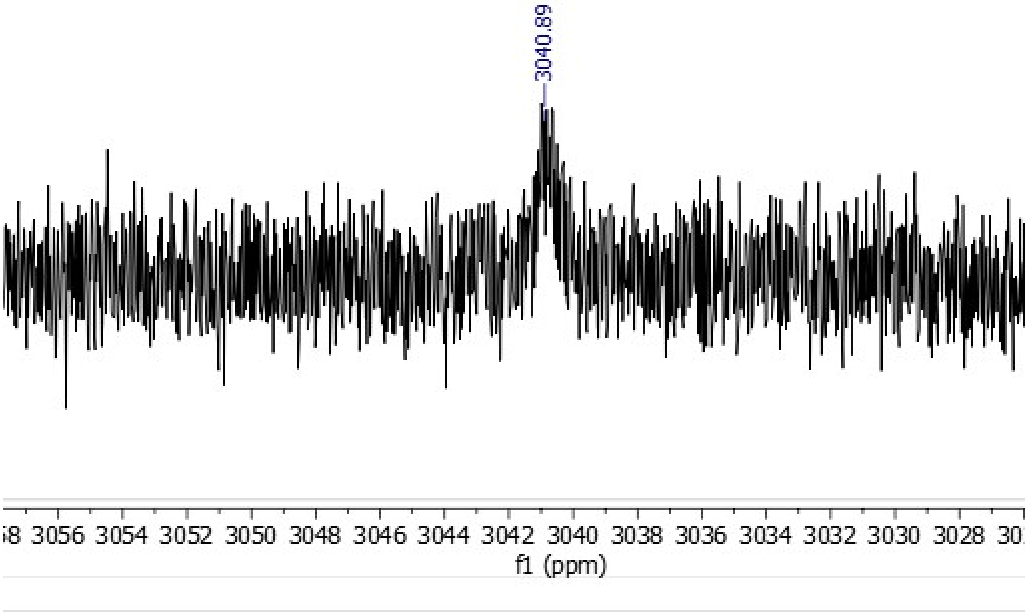

Figure S32. $^{125}Te\{^1H\}$ NMR (126 MHz, C_6D_6, 300 K) spectrum of **3**.

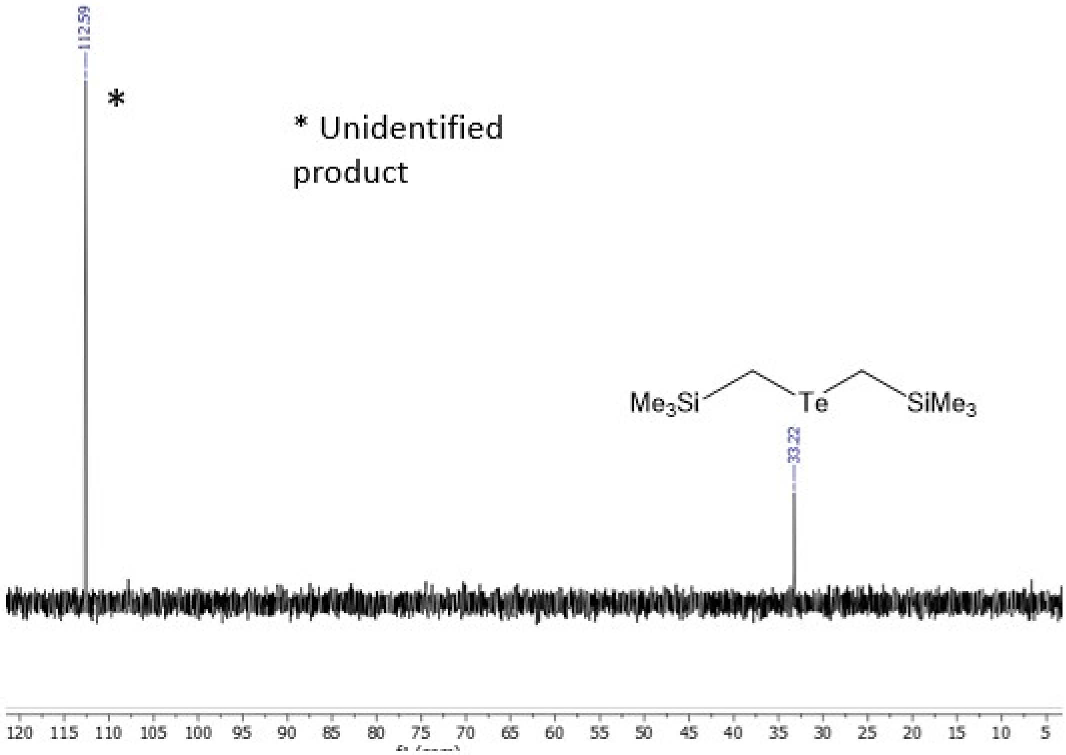

Figure S33. ^{125}Te{^{1}H} NMR (126 MHz, C_6D_6, 300 K) spectrum of pentane washings in the synthesis of **4**.

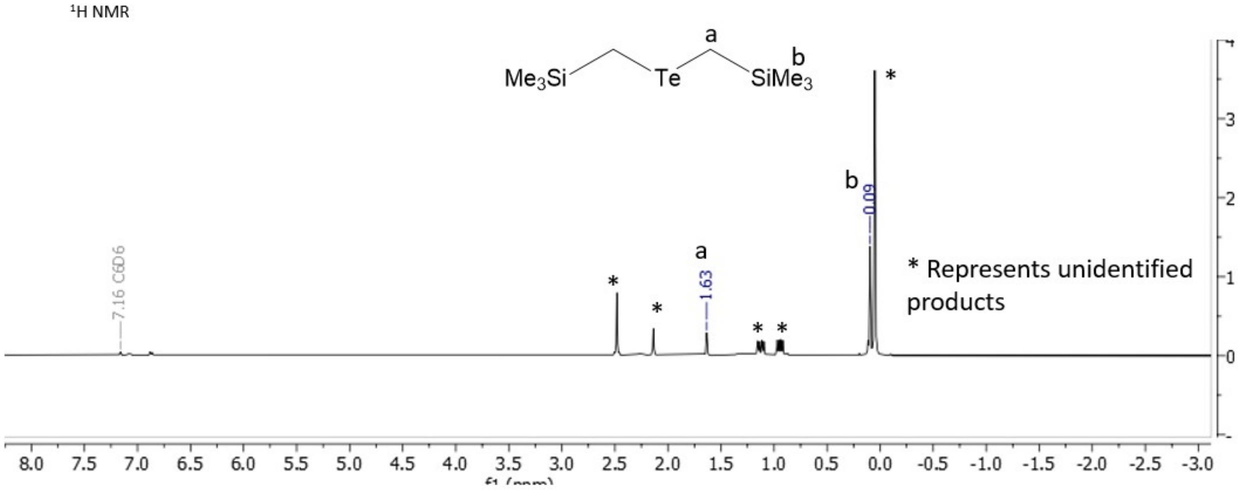

Figure S34. ^{1}H NMR (400 MHz, C_6D_6, 300 K) spectrum of pentane washings in the synthesis of **4**.

Note on the unidentified impurity from pentane washings of 4

To determine if the unidentified product is an oxidized organotellurium product such as $Te_2(CH_2SiMe_3)_2$, we checked the literature for several organotellurium compounds. First, the ^{1}H NMR shifts for a similar analogue $Te_2(C(SiMe_3)_3)_2$ is vastly different from the measured NMR shifts.[10] Second, the ^{125}Te NMR shifts for Te_2R_2 (p-MeC_6H_4) compounds are typically downfield at around 400 ppm.[11] Given these observations, we can confirm that the unidentified resonance is not an oxidized $Te_n(CH_2SiMe_3)_2$ (n = 2 or 3) product.[10] It is noteworthy that the pentane washes from the bis(telluride) titanate complex also had an impurity in the ^{125}Te NMR at 317 ppm that was not identified.[12]

NMR Yield Determination for the formation of 1

To determine the yield for the formation of **1**, an internal standard, hexamethylbenzene ($C_6(CH_3)_6$) was chosen as the internal reference resonance did not overlap with any of the product resonances and the internal standard remained unreactive during the course of the reaction. This was confirmed by the presence of internal standard both before and after the reaction, and no new product formation in the NMR spectrum besides the expected impurities and unidentified products from the reaction that was conducted without the internal standard. The -Me groups integrating to 9H's in the product were compared relative to the $C_6(CH_3)_6$ (18 H's).

$$c_{product} = \frac{int_{product}}{int_{standard}} \times \frac{18}{6} \times c_{standard}$$

To a -35 °C solution of $LiCH_2SiMe_3$ (25 mg, 266 µmol) in pentane (2 mL) was slowly added -35 °C suspension of Te (34 mg, 266 µmol) in THF (1 mL). Internal standard, hexamethylbenzene (3.6 mg) was added to this reaction mixture. The reaction was allowed to stir for an hour after which solvent was removed under vacuum. The pale yellow oil was dissolved in pentane filtered through Celite and dried under vacuum to give **1** (62 mg, 81% yield, 233 µmol).

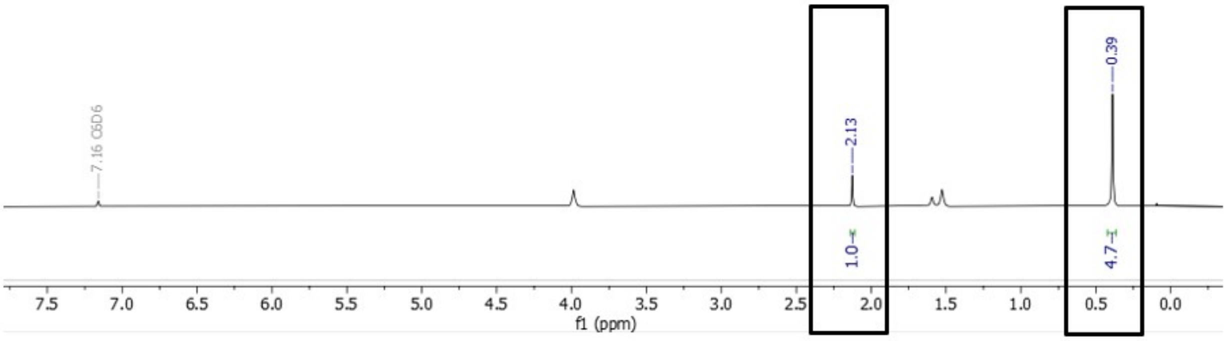

Figure S35. ^{1}H NMR (500 MHz, C_6D_6, 300 K) of **1**. The internal standard resonance and the -$SiMe_3$ resonances are highlighted in the box.

UV-vis spectroscopy

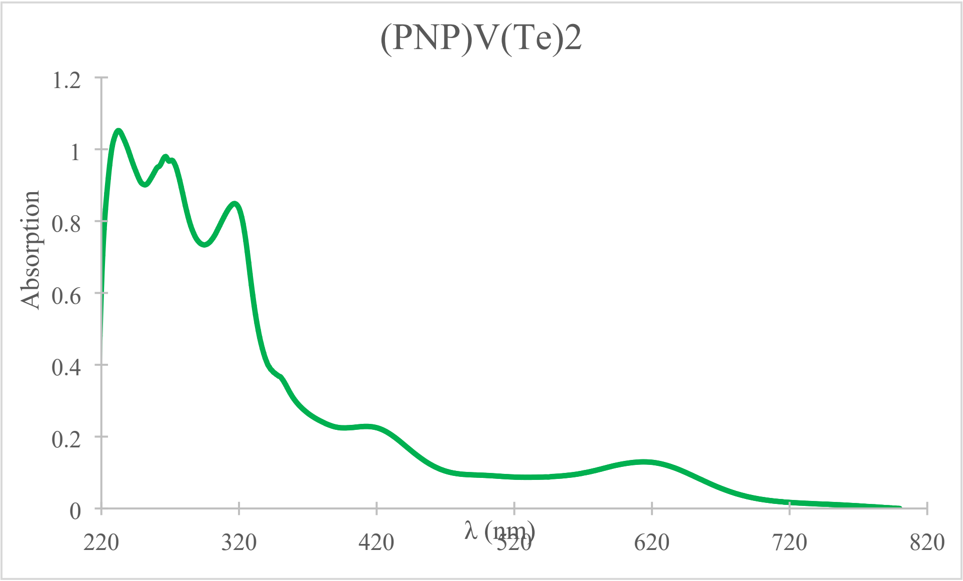

Figure S36. UV-vis spectrum (0.056 mM in THF at 300K) of $(PNP)V(Te)_2$.

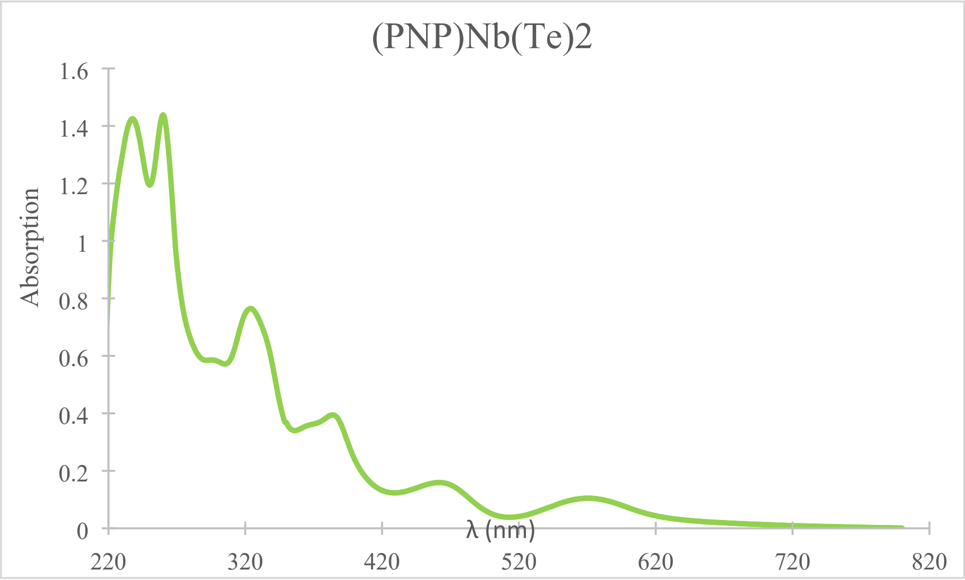

Figure S37. UV-vis spectrum (0.056 mM in THF at 300K) of **3**.

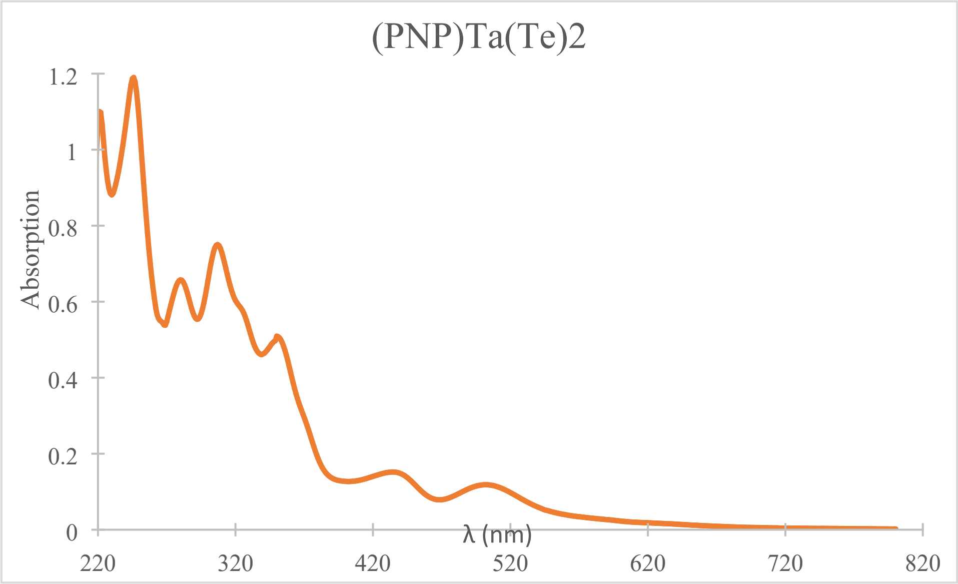

Figure S38. UV-vis spectrum (0.056 mM in THF at 300K) of **4**.

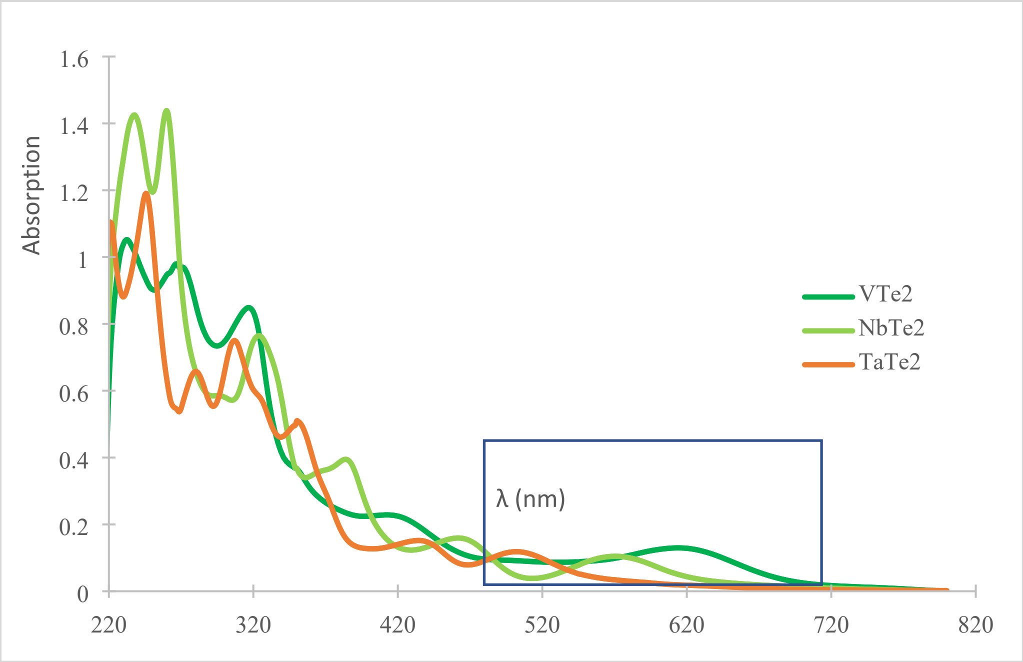

Figure S39. Overlapped UV-vis spectra (0.056 mM in THF at 300K) of bis(telluride) complexes with the LMCT transitions highlighted with a box.

X-Ray Crystallography

Crystallographic data for are summarized in **Tables S1-S3**. Suitable crystals for X-ray analysis of **2-4** were placed on the end of a Cryoloop coated in NVH oil. X-ray intensity data were collected on Rigaku XtaLAB Synergy-i diffractometer[13] equipped with an HPC area detector (HyPix 3000HE) employing confocal multilayer optic-monochromated Mo-Kα radiation (λ=0.71073Å) at a temperature of 100K. Preliminary indexing was performed from a series of thirty 0.5° rotation frames with exposures of 5 seconds. Rotation frames were integrated using CrysAlisPro,[13] producing a listing of unaveraged F^2 and $\sigma(F^2)$ values. The intensity data were corrected for Lorentz and polarization effects and for absorption using SCALE3 ABSPACK[14] (minimum and maximum transmission 0.84251, 1.00000). The structure was solved by direct or dual space methods - SHELXT 2014/5.[15] Refinement was by full-matrix least squares based on F^2 using SHELXL-2018.[16] All reflections were used during refinement. The weighting scheme used was $w=1/[\sigma^2(F_o^2) + (0.0771P)^2 + 0.0000P]$ where $P = (F_o^2 + 2F_c^2)/3$. Non-hydrogen atoms were refined anisotropically and hydrogen atoms were refined using a riding model.

The $[Li(12\text{-crown-}4)]^+$ complex (**2**) lies on a crystallographic center-of-symmetry (at 1, ½, ½), which causes this complex to be disordered because $[Li(12\text{-crown-}4)]^+$ does not have a molecular inversion center. The $[Me_3Si\text{-}CH_2\text{-}Te]^-$ complex lies on a crystallographic mirror plane (at x, ¼, z), which also disorders $[Me_3Si\text{-}CH_2\text{-}Te]^-$ because it does not have a molecular mirror.

These results were checked using the IUCR's CheckCIF routine.

Table S1. Summary of Structure Determination of Compound 2

Empirical formula	$C_{20}H_{43}LiO_8SiTe$
Formula weight	574.17
Diffractometer	Rigaku XtaLAB Synergy-i (HyPix 3000HE)
Temperature/K	100
Crystal system	orthorhombic
Space group	Pnma
a	10.9891(2)Å
b	19.9870(3)Å
c	12.3245(2)Å
Volume	2706.94(8)Å^3
Z	4
d_{calc}	1.409 g/cm^3
μ	1.181 mm^{-1}
F(000)	1184.0
Crystal size, mm	0.36 × 0.2 × 0.2
2θ range for data collection	3.882 - 54.952°
Index ranges	$-14 \leq h \leq 14$, $-25 \leq k \leq 25$, $-16 \leq l \leq 15$
Reflections collected	79546
Independent reflections	3200[R(int) = 0.0431]
Data/restraints/parameters	3200/168/277
Goodness-of-fit on F^2	1.046
Final R indexes [I>=2σ (I)]	$R_1 = 0.0242$, $wR_2 = 0.0521$
Final R indexes [all data]	$R_1 = 0.0303$, $wR_2 = 0.0536$
Largest diff. peak/hole	0.50/-0.72 eÅ^{-3}

Table S2. Summary of Structure Determination of Compound 3

Empirical formula	$C_{36}H_{59}NNbO_{2.5}P_2Te_2$
Formula weight	955.89
Diffractometer	Rigaku XtaLAB Synergy-i
Temperature/K	100(2)
Crystal system	triclinic
Space group	P**Error!**
a	11.5657(2)Å
b	13.6873(2)Å
c	14.3677(2)Å
α	64.365(2)°
β	77.8010(10)°
γ	88.8530(10)°
Volume	1997.68(6)Å^3
Z	2
d_{calc}	1.589 g/cm^3
μ	1.844 mm^{-1}
F(000)	954.0
Crystal size, mm	0.52 × 0.17 × 0.15
2θ range for data collection	4.27 - 54.968°
Index ranges	$-14 \leq h \leq 15$, $-17 \leq k \leq 17$, $-18 \leq l \leq 18$
Reflections collected	63004
Independent reflections	9091[R(int) = 0.0471]
Data/restraints/parameters	9091/50/434
Goodness-of-fit on F^2	1.104
Final R indexes [I>=2σ (I)]	$R_1 = 0.0562$, $wR_2 = 0.1467$
Final R indexes [all data]	$R_1 = 0.0684$, $wR_2 = 0.1526$
Largest diff. peak/hole	2.65/-1.19 eÅ^{-3}

Table S3. Summary of Structure Determination of Compound 4

Empirical formula	$C_{36}H_{60}NO_{2.5}P_2TaTe_2$
Formula weight	1044.94
Diffractometer	Rigaku XtaLAB Synergy-i
Temperature/K	100
Crystal system	triclinic
Space group	P**Error!**
a	11.5337(2)Å
b	13.6583(3)Å
c	14.3715(3)Å
α	64.384(2)°
β	77.963(2)°
γ	88.962(2)°
Volume	1989.88(8)Å^3
Z	2
d_{calc}	1.744 g/cm^3
μ	4.311 mm^{-1}
F(000)	1020.0
Crystal size, mm	0.37 × 0.13 × 0.13
2θ range for data collection	3.622 - 54.968°
Index ranges	$-14 \leq h \leq 14$, $-17 \leq k \leq 17$, $-18 \leq l \leq 18$
Reflections collected	60965
Independent reflections	9059[R(int) = 0.0489]
Data/restraints/parameters	9059/50/434
Goodness-of-fit on F^2	1.053
Final R indexes [I>=2σ (I)]	$R_1 = 0.0485$, $wR_2 = 0.1348$
Final R indexes [all data]	$R_1 = 0.0575$, $wR_2 = 0.1396$
Largest diff. peak/hole	3.42/-2.58 eÅ^{-3}

Computational Details

All calculations were carried out using density functional theory[17] (DFT) implemented in the ADF 2019 package program,[18-22] 10 functionals[23-31] were employed for the benchmark study. All calculations were done with no frozen core to have an all-electron basis set, and the accuracy of integration was set to have good numerical qualities. Scalar zeroth-order regular approximation was employed for all calculations. Geometry optimizations were performed with the DZP basis set, and UV-Vis spectra were obtained with the TZ2P basis set with the optimized geometry. Excitation energies were evaluated on the Davidson method, and Tamm-Dancoff approximation[32] was applied to extract natural transition orbitals (NTOs). 10 lowest excited states were evaluated for each functional to get accurate excitation energies. Charge-transfer diagnostic overlap matrix[33] and hole-electron distance[34] of NTOs were evaluated for detailed charge-transfer analysis.

DFT Benchmark Study

We performed a DFT benchmark study to find an optimal functional of the electronic structure of bis(telluride) complexes accurately at the same time. GGA (PBE[23]), meta-GGA (M06L[24]), hybrid GGA (B3LYP[25], PBE0[26], BHandHLYP[27]), hybrid meta-GGA (TPSSh20[28], M06[29], MN15[30], M06-2X[31]) and range-separated hybrid (CAM-B3LYP[31]) type functionals are included in the benchmark study (**Table S4**).

Table S4. Density Functionals Used for the Benchmark Study

Density Functional	Type	% HF Exch.	Ref.
PBE	GGA	-	16
M06L	meta-GGA	-	17
B3LYP	hybrid GGA	20	18
PBE0	hybrid GGA	25	19
BHandHLYP	hybrid GGA	50	20
TPSSh	hybrid meta-GGA	10	21
M06	hybrid meta-GGA	27	22
MN15	hybrid meta-GGA	44	23
M06-2X	hybrid meta-GGA	54	24
CAM-B3LYP	range-separated hybrid GGA	19–65	25

Figure S40 illustrates the calculated UV-Vis spectrum and their comparison with experimentally obtained absorption wavelengths of (PNP)Nb$(Te)_2$ (**3**). PBE and M06L functionals underestimate the excitation energies showing absorption wavelengths longer than 650 nm, while B3LYP, TPSSh, M06, PBE0 and MN15 functionals provide the first maximum oscillator strength near 574 nm. Functionals with %HF more than 50% (BHandHLYP, M06-2X, and CAM-B3LYP) overestimate excitation energies, resulting in an absorption wavelength of less than 500 nm. **Figure S41** provides similar trends with (PNP)Ta$(Te)_2$ (**4**) implying that the electronic structures of the two complexes are similar.

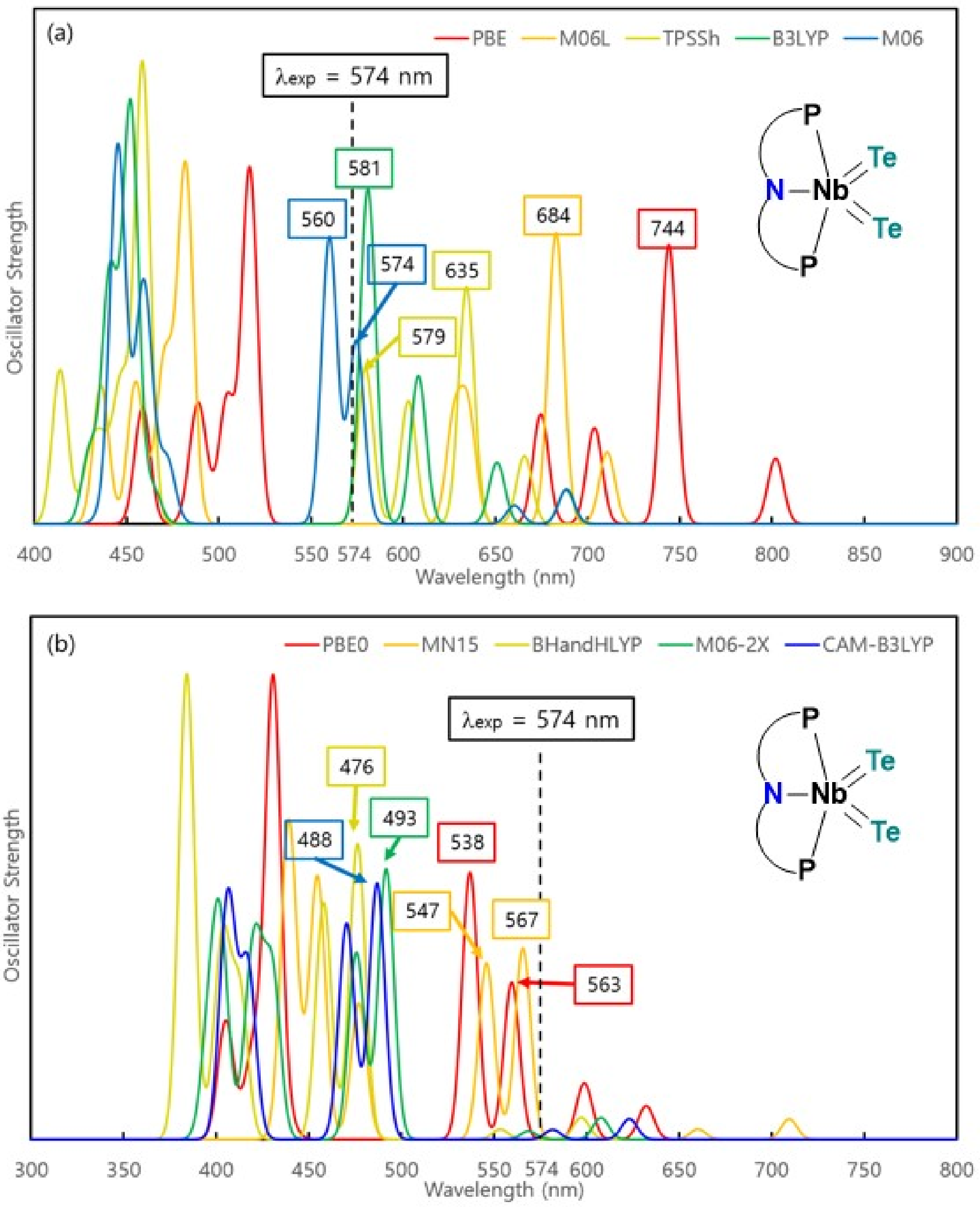

Figure S40. Estimated UV-Vis spectrum of **3** with (a) GGA and hybrid GGA functionals (b) hybrid meta-GGA and range-separated hybrid functionals. Absorption wavelengths near 574 nm are highlighted with square boxes.

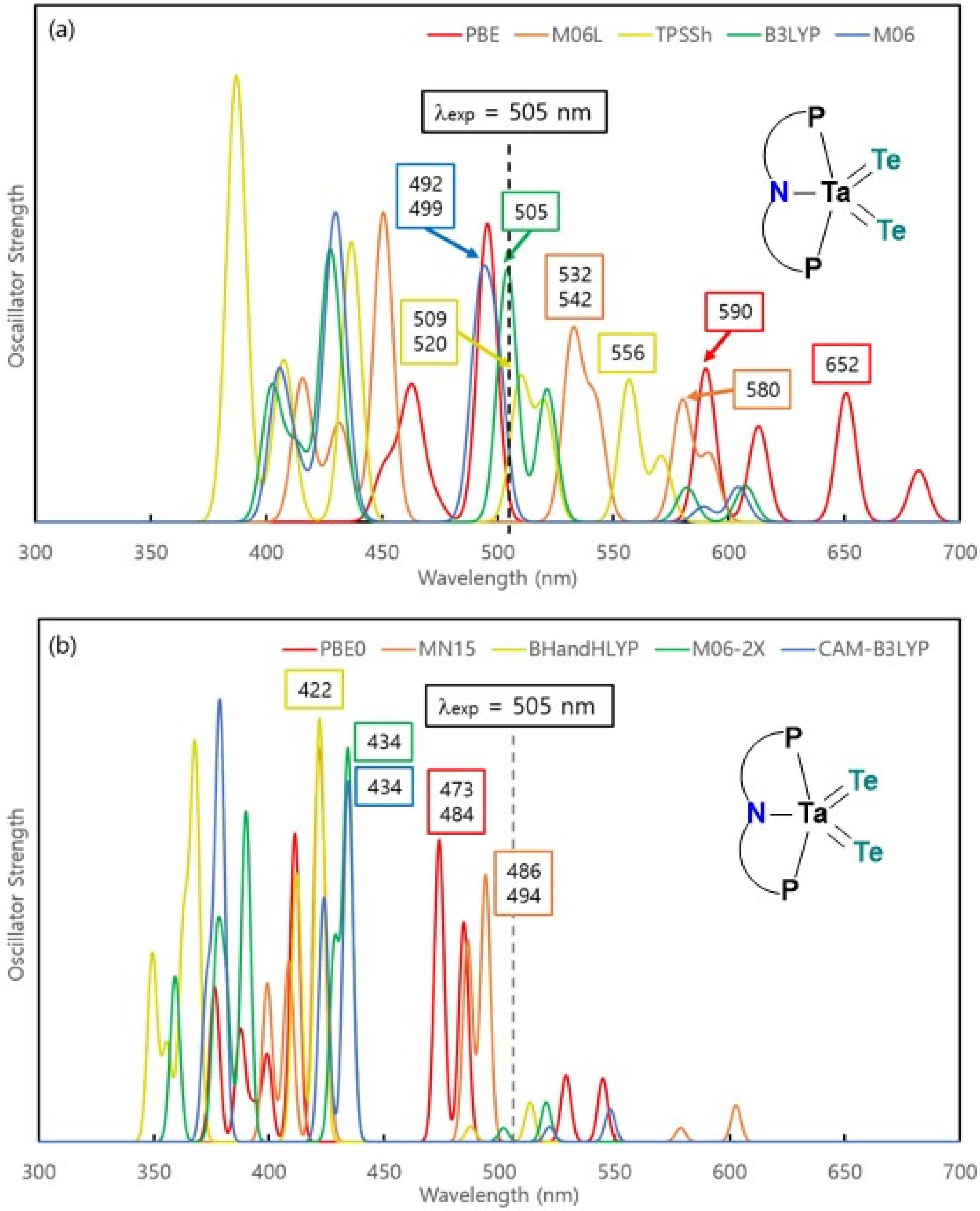

Figure S41. Estimated UV-Vis spectrum of **4** with (a) GGA and hybrid GGA functionals (b) hybrid meta-GGA and range-separated hybrid functionals. Absorption wavelengths near 505 nm are highlighted with square boxes.

From the analysis of complexes **3** and **4**, we chose B3LYP, M06, PBE0, and MN15 as promising functionals for further study. In **Figure S42**, the theoretical prediction of the UV-Vis spectrum of $(PNP)V(Te)_2$ complex is shown. Among the chosen functionals, only MN15 could properly mimic the non-zero oscillator strength at 621 nm, which is the experimentally observed absorption wavelength.

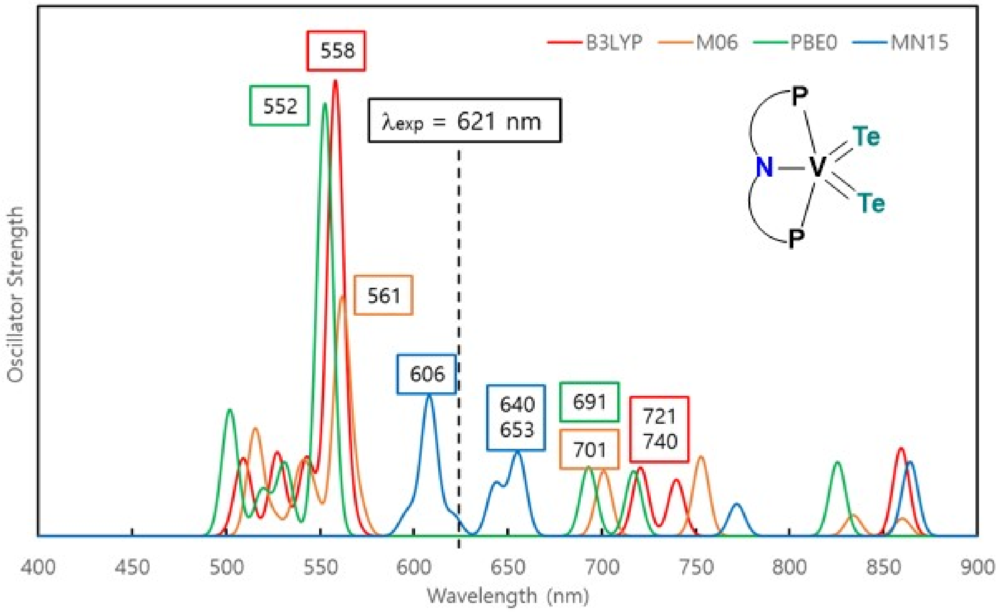

Figure S42. Estimated UV-Vis spectrum of $(PNP)V(Te)_2$ with chosen functionals. Absorption wavelengths near 621 nm are highlighted with square boxes.

Excitation Energy Profiles

Based on the functional benchmark study, we used MN15 functional for the charge-transfer analysis. DFT-calculated excitation profiles for 10 lowest excited states of three complexes are summarized in **Table S5–S7**. The excitations corresponding to experimentally observed absorption are highlighted in bold.

Table S5. Excitation Profiles of (PNP)V(Te)$_2$

State	Wavelength (nm)	Excitation Energy (eV)	Oscillator Strength	Major Contributions
**Exp**	**621**	**1.997**	**Non-zero**	**Two most contributing orbital pairs**
S1	853.2	1.453	0.0025	HOMO → LUMO (62.5%)<br>HOMO–1 → LUMO (17.6%)
S2	826.1	1.501	0.0006	HOMO → LUMO+1 (55.8%)<br>HOMO–1 → LUMO+1 (25.6%)
S3	784.6	1.580	0.0053	HOMO–1 → LUMO (54.8%)<br>HOMO–2 → LUMO (15.5%)
S4	707.3	1.753	0.0018	HOMO–1 → LUMO+1 (52.6%)<br>HOMO → LUMO+1 (23.3%)
**S5**	**606.0**	**2.046**	**0.0075**	**HOMO → LUMO+2 (40.9%)**<br>**HOMO–2 → LUMO+2 (17.7%)**
S6	595.0	2.084	0.0054	HOMO–5 → LUMO (51.7%)<br>HOMO–1 → LUMO (14.9%)
S7	571.9	2.168	0.0058	HOMO–5 → LUMO+1 (32.3%)<br>HOMO → LUMO+2 (19.3%)
S8	567.1	2.186	0.0045	HOMO–3 → LUMO+1 (65.0%)<br>HOMO–1 → LUMO+3 (11.7%)
S9	553.0	2.242	0.0034	HOMO → LUMO+3 (44.7%)<br>HOMO–2 → LUMO+3 (28.3%)
S10	550.6	2.252	0.0001	HOMO–3 → LUMO (45.9%)<br>HOMO–1 → LUMO+2 (31.3%)

Table S6. Excitation Profiles of Complex 3

State	Wavelength (nm)	Excitation Energy (eV)	Oscillator Strength	Major Contributions
**Exp**	**574**	**2.160**	**Non-zero**	**Two most contributing orbital pairs**
S1	682.9	1.816	0.0016	HOMO–1 → LUMO (59.6%)<br>HOMO → LUMO (32.7%)
S2	638.4	1.942	0.0006	HOMO–1 → LUMO+1 (50.7%)<br>HOMO → LUMO+1 (37.9%)
**S3**	**564.9**	**2.195**	**0.0131**	**HOMO → LUMO (55.1%)**<br>**HOMO–1 → LUMO (32.4%)**
S4	545.7	2.272	0.0110	HOMO → LUMO (48.8%)<br>HOMO–1 → LUMO (39.9%)
S5	470.5	2.635	0.0073	HOMO–2 à LUMO (30.1%)<br>HOMO–5 à LUMO (22.6%)
S6	468.8	2.645	0.0027	HOMO–3 → LUMO (45.3%)<br>HOMO–5 → LUMO+1 (13.3%)
S7	448.2	2.766	0.0134	HOMO → LUMO+2 (65.4%)<br>HOMO–4 → LUMO (23.9%)
S8	443.5	2.796	0.0090	HOMO–3 → LUMO+1 (27.6%)<br>HOMO–4 → LUMO+1 (23.2%)
S9	437.7	2.833	0.0055	HOMO–1 → LUMO+2 (36.7%)<br>HOMO–4 → LUMO (18.1%)
S10	435.3	2.848	0.0043	HOMO–4 → LUMO+1 (41.8%)<br>HOMO–2 → LUMO (14.5%)

Table S7. Excitation Profiles of Complex 4

State	Wavelength (nm)	Excitation Energy (eV)	Oscillator Strength	Major Contributions
**Exp**	**505**	**2.455**	**Non-zero**	**Two most contributing orbital pairs**
S1	603.1	2.056	0.0024	HOMO → LUMO (60.5%)<br>HOMO–1 → LUMO (30.9%)
S2	576.2	2.151	0.0010	HOMO → LUMO+1 (65.1%)<br>HOMO–1 → LUMO+1 (24.7%)
**S3**	**495.2**	**2.504**	**0.0188**	**HOMO–1 → LUMO (58.9%)**<br>**HOMO → LUMO (28.9%)**
S4	485.0	2.557	0.0150	HOMO–1 → LUMO+1 (65.4%)<br>HOMO → LUMO+1 (23.0%)
S5	422.1	2.937	0.0013	HOMO–3 → LUMO (47.8%)<br>HOMO–5 → LUMO+1 (13.5%)
S6	421.5	2.941	0.0107	HOMO–2 → LUMO (32.5%)<br>HOMO–3 → LUMO+1 (31.3%)
S7	417.6	2.969	0.0189	HOMO → LUMO+2 (63.3%)<br>HOMO–4 → LUMO (21.9%)
S8	404.5	3.065	0.0126	HOMO–3 → LUMO+1 (41.8%)<br>HOMO–2 → LUMO (22.2%)
S9	403.2	3.075	0.0011	HOMO–1 → LUMO+2 (60.5%)<br>HOMO–4 → LUMO (12.7%)
S10	394.4	3.144	0.0105	HOMO–4 → LUMO+1 (72.8%)<br>HOMO–5 → LUMO (5.6%)

Charge-Transfer Analysis

Figure S43 illustrates the most contributing NTO pair and the two most contributing orbital pairs from excitation energy profiles in Table **S5–S7**. Both **3** and **4** show strong excitations from HOMO and HOMO–1 to LUMO with an NTO contribution of more than 92%. The low HOMO-LUMO gap of the vanadium complex results in the excitation from HOMO and HOMO–2 to LUMO+2. One can distinguish the charge-transfer character with the electron density distribution of NTO pairs, where the character is minimized for the vanadium complex due to the metal-centered hole and electron. In contrast, ligand-centered NTO holes highlight the charge-transfer character of **3** and **4**.

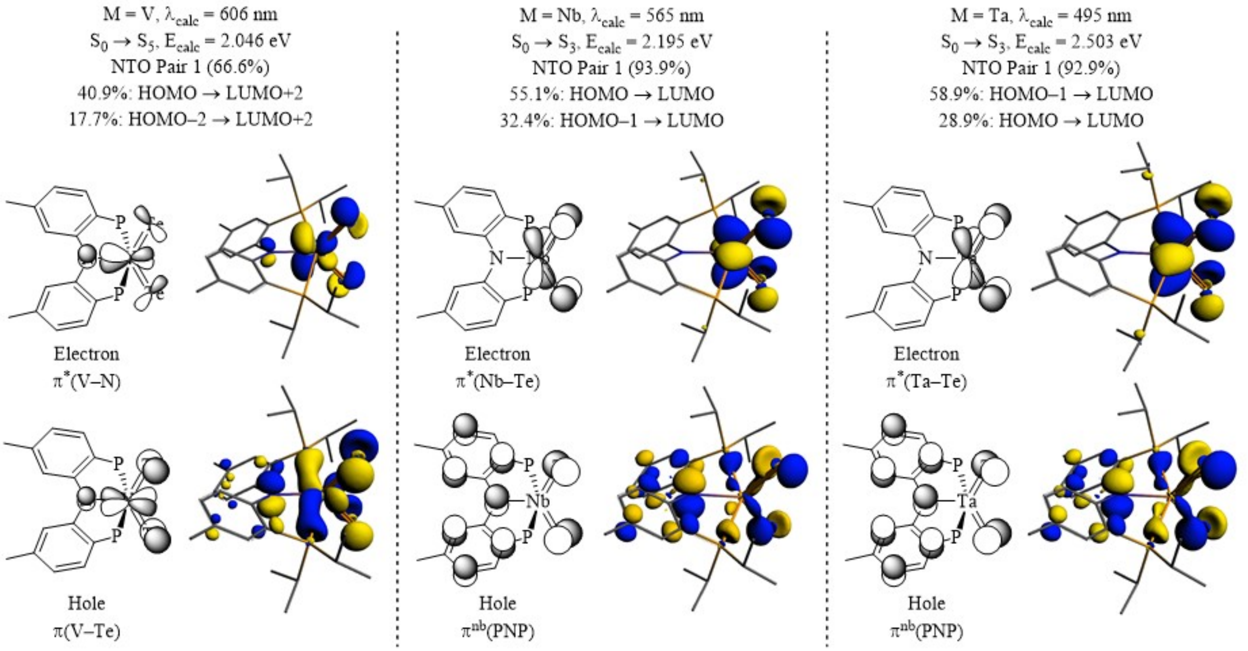

Figure S43. Excitation profiles of three complexes with the most contributing NTO pair.

We conducted further analysis in terms of the charge-transfer diagnostic overlap matrix (λ),[33] average hole-electron distance (Δr),[34,] and transition dipole moment (μ) for the quantitative comparison of three complexes (**Table S8**). λ is computed as a spatial overlap between orbitals involved in the excitation, being a value between 0 and 1. Even though this descriptor is very intuitive, there are some reports that it cannot properly diagnose charge-transfers induced by large dipole moment changes.[35] Three metal complexes show negligible difference with the λ value near 0.5, contradicting the NTO analysis. Δr is an alternative descriptor providing information on an average distance between NTO hole and electron centroids and is known to resolve problematic cases with λ. The computed distance differs from 0.494 Å (M = Ta) to 1.127 Å (M = Nb) following the trend observed with the orbital transition compositions (**Figure 3 in the manuscript**). Finally, the transition dipole moment was calculated with a z-axis on the M–N bond and a y-

axis on the direction between two phosphine ligands. The vanadium complex exclusively shows a strong transition through the z-axis, reflecting the different excitation profiles with **3** and **4**.

Table S8. **Charge-transfer descriptors**

M	λ	Δr (Å)	μ(x, y, z)
V	0.594	0.669	(0.001, 0.003, 0.386)
Nb	0.491	1.127	(0.489, -0.026, -0.054)
Ta	0.553	0.494	(0.553, 0.002, -0.021)

Cartesian Coordinates of the Optimized Geometries

========================== (PNP)V(Te)$_2$

=========================
V 0.000251999 -0.003776000 0.011665999
Te 0.261265993 1.961027979 -1.275341033
Te -0.254851996 -1.973508000 -1.266232967
P -2.301412105 0.392628014 0.631922006
P 2.301347017 -0.405187010 0.630550980
N 0.002240000 0.001573999 2.060095071
C -1.072725057 -0.525326013 2.782927989
C -0.952893972 -1.168400049 4.019752025
C -2.068855047 -1.704769015 4.642291069
C -3.341720104 -1.631732940 4.078361034
C -3.460398912 -0.997286975 2.845628976
C -2.351779937 -0.459271013 2.207830905
C -4.538566112 -2.203669071 4.781485080
C -2.692645072 2.149243116 1.075235962
C -4.125210762 2.339922904 1.559645056
C -1.718850970 2.670028924 2.128113031
C -3.779448032 -0.146910995 -0.306584000
C -3.835531949 -1.643846035 -0.579245984
C -3.885320901 0.648553013 -1.603816986
C 1.078377962 0.534493982 2.774785041
C 0.961157023 1.187096953 4.007690906
C 2.077297925 1.728698015 4.621139049
C 3.348444938 1.651023030 4.052071094
C 3.464570045 1.006695032 2.826927900
C 2.354171991 0.463506996 2.196976900
C 4.537390232 2.257675886 4.739468097
C 3.779545068 0.122498996 -0.315178990
C 3.831904888 1.614354968 -0.613644003
C 3.883825063 -0.691497981 -1.601027011
C 2.690607070 -2.158067941 1.091074943
C 4.121343135 -2.341731071 1.583120942
C 1.715085029 -2.667979955 2.147250890
H -4.966300964 -1.483392000 5.482838153
H -4.271417140 -3.097326993 5.346323013
H -5.318282127 -2.477668046 4.070233821
H -2.534065008 2.708879947 0.144942000
H -4.867242813 2.162914037 0.780021011
H -4.251968860 3.367912054 1.907884955
H -4.338950157 1.680235981 2.406692028
H -1.848126053 2.120053052 3.065241098
H -1.934303045 3.723022937 2.325064897
H -0.678484022 2.583947896 1.812531948
H -4.624744892 0.109645999 0.343876987
H -3.177397012 -1.910379052 -1.407127022
H -4.853774070 -1.915967941 -0.868687987
H -3.550528049 -2.243437051 0.287651985
H -3.948189020 1.724692940 -1.430577039
H -4.776731967 0.342058002 -2.155951976
H -3.011997938 0.456010997 -2.233973979
H -0.009050000 1.277618050 4.481433868
H 1.958281040 2.242949962 5.569610118
H 4.441428184 0.940081000 2.355259895
H 4.718979835 1.783697962 5.706047058
H 4.387214183 3.324310064 4.916544914
H 5.437939167 2.142990112 4.136345863
H 4.626239776 -0.122215002 0.337817996
H 3.155616998 1.864488005 -1.432240009
H 4.843453884 1.880082011 -0.931502997
H 3.564816951 2.229408979 0.247886002
H 3.960086107 -1.764505982 -1.413205027
H 4.766811847 -0.382373988 -2.165241956
H 3.003048896 -0.516794979 -2.226558923
H 2.535465002 -2.727879047 0.166547998
H 4.867622852 -2.166748046 0.807003021
H 4.249527931 -3.367140054 1.938652038
H 4.326880931 -1.676030993 2.427740097
H 1.834756016 -2.097780942 3.073138952
H 1.940929055 -3.713993072 2.366544008
H 0.675719022 -2.599127054 1.824466943
H 0.019282000 -1.257869005 4.489129066
H -1.943613052 -2.212194919 5.593455791
H -4.437549114 -0.938232004 2.373203039

=========================
(PNP)Nb(Te)$_2$ (**3**)
=========================
Nb -0.024558773 -0.004871684 0.010866823
Te -2.029988288 -0.738169074 -1.300819754
Te 1.923521399 0.741900503 -1.382927775
P 0.721066892 -2.336841344 0.783875346
P -0.714855432 2.339338302 0.788984000
N -0.012759368 -0.004994934 2.188350439
C -0.974680364 0.718643426 2.904124021
C -1.515998244 0.289508014 4.123285770
C -2.496848821 1.029375910 4.759785175
C -2.989810228 2.220594882 4.225478172
C -2.455576181 2.647131919 3.017206430
C -1.472904443 1.914229035 2.360797405
C -4.072506427 2.993637084 4.919880390
C -1.892830371 3.426831245 -0.096295140
C -3.226047515 2.756561994 -0.395854115
C -1.241320490 3.941234588 -1.375703930
C 0.685689985 3.457372188 1.242640972
C 0.214958861 4.790550708 1.809686422
C 1.619041442 2.768564224 2.233343124
C 0.945435404 -0.725496053 2.910347700
C 1.461734414 -0.307231426 4.144310951
C 2.439370870 -1.045217752 4.787146568
C 2.954468965 -2.224445819 4.246753692
C 2.446812391 -2.639047384 3.022606849
C 1.466518521 -1.908166289 2.360077857
C 4.022654056 -3.000290155 4.960531711
C 1.920042514 -3.393273591 -0.109303779
C 3.244884490 -2.698287725 -0.388322710
C 1.279908061 -3.889463186 -1.402465820
C -0.657534539 -3.484943628 1.232469320
C -1.589903593 -2.828105688 2.246050119
C -0.164173334 -4.822185516 1.770723700
H -1.166537642 -0.634267866 4.568834304
H -2.904634237 0.663129687 5.696699619

H -2.826905727 3.565958499 2.572433471
H -5.010455608 2.434821844 4.929213523
H -4.254924774 3.943178176 4.416822433
H -3.802767753 3.208384275 5.955132961
H -2.073554277 4.270385265 0.581476569
H -3.112555265 2.022830724 -1.194552302
H -3.939492225 3.511537790 -0.735720217
H -3.647323369 2.249250411 0.474339842
H -0.330276548 4.511302947 -1.182138800
H -1.938792943 4.589659214 -1.910834550
H -0.985349178 3.103420257 -2.031443357
H 1.229783654 3.614607810 0.302718579
H -0.327236384 5.395674705 1.081705570
H 1.081727743 5.369095325 2.138720035
H -0.424839109 4.633470058 2.683989048
H 1.094073176 2.574292898 3.173487186
H 2.462581157 3.428218126 2.449955463
H 2.009892702 1.825339198 1.848637700
H 1.095255494 0.606659173 4.596452713
H 2.827587366 -0.686633825 5.735328674
H 2.837851047 -3.546974658 2.571139097
H 3.675297975 -3.344165563 5.936592578
H 4.912715911 -2.389268398 5.120897293
H 4.319265365 -3.875998497 4.383246898
H 2.108479499 -4.246965408 0.553306519
H 3.122528314 -1.940554499 -1.162887334
H 3.966101884 -3.434403181 -0.751938104
H 3.660290718 -2.212964296 0.496805250
H 0.375894367 -4.475464820 -1.223738431
H 1.987041354 -4.515512943 -1.951220989
H 1.013436079 -3.040130853 -2.039125680
H -1.209205389 -3.633526563 0.295569986
H -2.425029039 -3.501142024 2.453738689
H -1.993711590 -1.880121350 1.887023329
H -1.058523893 -2.650818586 3.186152458
H 0.485531091 -4.672820091 2.639018535
H 0.373711794 -5.409627437 1.025304675
H -1.020747900 -5.414299964 2.102312803

==========================

(PNP)Ta(Te)$_2$ (**4**)

==========================

Ta 0.000000000 0.000000000 0.000000000
Te -1.996619992 -0.636005271 -1.367902026
Te 2.024955722 0.672317769 -1.307573291
P 0.632592843 -2.360406690 0.738805744
P -0.620119709 2.354079449 0.754372407
N -0.023126916 -0.016695057 2.141606741
C -0.951690843 0.749145140 2.864413407
C -1.506465228 0.338295204 4.082359297
C -2.438010659 1.127996756 4.732536112
C -2.865298341 2.351410229 4.212947537
C -2.321383744 2.754601391 3.001304986
C -1.388829628 1.970733931 2.330453350
C -3.883617882 3.183366580 4.936231240
C -1.760320111 3.468111127 -0.143936612
C -3.137262810 2.867870807 -0.394111327
C -1.108462460 3.902641581 -1.452441216
C 0.818312039 3.429032704 1.196939463
C 0.400289715 4.802255388 1.705507430
C 1.698586283 2.735249465 2.231619055
C 0.907500700 -0.773649133 2.870699048
C 1.444791829 -0.364581480 4.096506759
C 2.388194497 -1.142152010 4.744851302
C 2.843753460 -2.350937271 4.217082401
C 2.315107501 -2.754472339 2.998332710
C 1.372214964 -1.982467211 2.329436405
C 3.879880150 -3.163475320 4.935444818
C 1.806705446 -3.445089357 -0.155826683
C 3.161220799 -2.801488527 -0.418412780
C 1.163490168 -3.925705964 -1.453092553
C -0.803705877 -3.450240778 1.142494098
C -1.742740299 -2.761223068 2.128814427
C -0.398571727 -4.811460660 1.691115545
H -1.204843647 -0.607270965 4.517187002
H -2.859233452 0.779295983 5.670127600
H -2.644622389 3.696172087 2.565719084
H -4.841901574 2.664330764 4.999766946
H -4.050571910 4.130218495 4.422855955
H -3.557337709 3.406119211 5.953276274
H -1.880264263 4.340805936 0.509774704
H -3.084497136 2.104569783 -1.170706340
H -3.811040356 3.654821812 -0.742489372
H -3.567998150 2.413130121 0.499985935
H -0.153492601 4.409667334 -1.295801989
H -1.773278585 4.583896367 -1.988173769
H -0.927210820 3.032046575 -2.089790971
H 1.387828650 3.527081562 0.264329548
H -0.084853135 5.409904831 0.940465917
H 1.286108915 5.343790187 2.046510320
H -0.274039040 4.709268203 2.562970669
H 1.148588539 2.606542029 3.169031225
H 2.571548575 3.359517535 2.436252764
H 2.046943069 1.758652776 1.890779384
H 1.123374428 0.569829428 4.540224331
H 2.795215596 -0.792600951 5.688284657
H 2.659260305 -3.685434674 2.556301062
H 3.556468841 -3.407568417 5.948601864
H 4.823757185 -2.619535654 5.008638365
H 4.074996204 -4.099267999 4.411487655
H 1.967270765 -4.303929961 0.506216890
H 3.090391919 -2.074505288 -1.227357609
H 3.868618872 -3.576334944 -0.724423170
H 3.563602885 -2.292612039 0.459895716
H 0.245713695 -4.491739645 -1.279140721
H 1.860022745 -4.569404965 -1.994682105
H 0.922165467 -3.071070070 -2.092310430
H -1.329117652 -3.567028267 0.185283212
H -2.589732378 -3.419981419 2.335941764
H -2.127895897 -1.814354256 1.747540856
H -1.223188469 -2.575974713 3.074011550

H   0.000453568 -4.715813809 2.705117323
H   0.343256556 -5.319467226 1.073354158
H -1.261837505 -5.462823139 1.725138752

X-Ray Absorption Spectroscopy

Te L_1 Edge

Solid samples for X-ray spectroscopic analysis were prepared in an inert-atmosphere glovebox. Solid samples were finely ground using an agate mortar and pestle with boron nitride (BN) into a homogeneous mixture comprising 5% (w/w) Te. These mixtures were pressed into 1 mm Al spacers and sealed with 38 μm Kapton tape. XAS data were obtained at the Stanford Synchrotron Radiation Lightsource (SSRL) at beamline 4-3 under ring conditions of 3 GeV and 500 mA. A Si(111) double-crystal monochromator was used. Internal energy calibrations were performed by assigning the first inflection points of a Ti foil spectrum to 4966 eV. Te L_1-edge data were obtained on samples held at ambient temperature within a He atmosphere, using fluorescence detection measured with a PIPS detector.

Data were collected from 4660 to 5340 eV. Three scans were measured and averaged with the Athena software package.[36] No spectral changes due to photodamage were observed after multiple scans for these complexes. Data were normalized in Athena using a linear function for the pre-edge (< 4920 eV) and a cubic function for the post edge (> 5000 eV) to produce the final spectra. E0 was set at 4960 eV. Peak fitting was performed with an in-house developed Monte-Carlo based peak fitting program and deemed acceptable when the residual absorbance was calculated to be <0.05 in the region between 4920 eV and 4960 eV.[37]

DFT Calculations

All calculations were performed in Orca 4.2.0.[38]

TDDFT calculations were performed at the def2-tzvpp(C, H, N, P, Te, Nb, Ta)/CP(PPP)(V)//B3LYP[39,40] level of theory. Geometries from the crystallographically derived coordinates for **V**, **3** and **4** were used. The RIJCOSX approximation and associated auxiliary basis sets were used.[41,42] Relativistic corrections were performed with the Zeroth Order Relativistic Approximation (ZORA).[43] The Lebedev 302 point integration grid (Orca Grid4) was selected for C, H, and N atoms, while the Lebedev 770 point integration grid (Orca Grid7) was used for V, Nb, Ta, Te, and P atoms. Solvent corrections were applied with the continuous polarizable continuum model (CPCM).[44] Calculated spectra were shifted by a scalar correction, derived from the average of the difference between the calculated and measured L_1 peak.

Peak fits

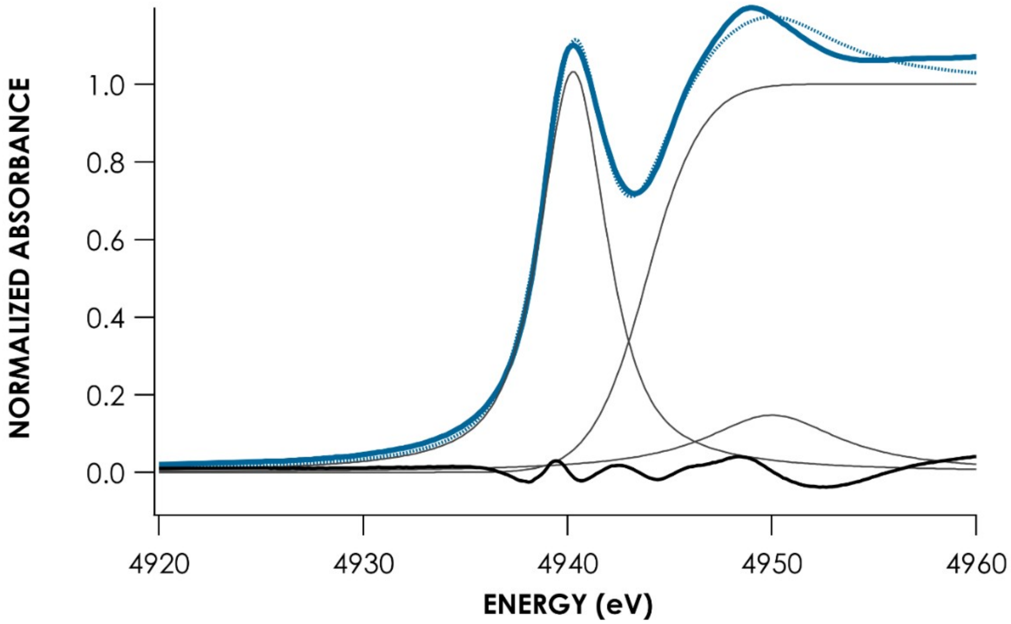

Figure S44. Overlay of the measured (solid) and fit (dashed) spectrum of **V**. Fit components including the edge jump plotted in grey. Residual absorption plotted in black.

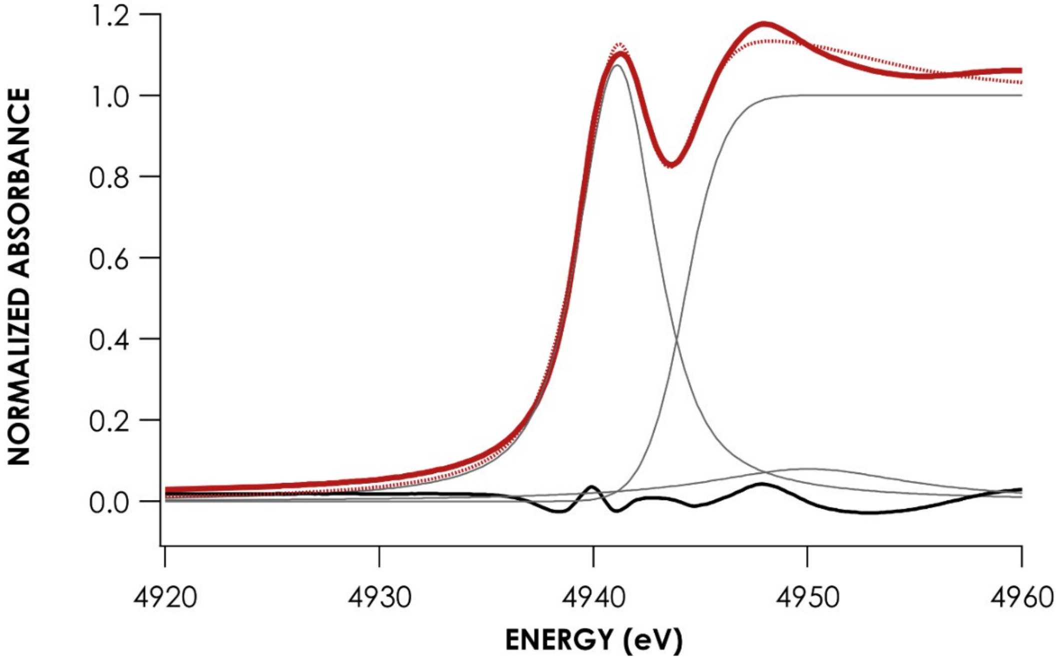

Figure S45. Overlay of the measured (solid) and fit (dashed) spectrum of **3**. Fit components including the edge jump plotted in grey. Residual absorption plotted in black.

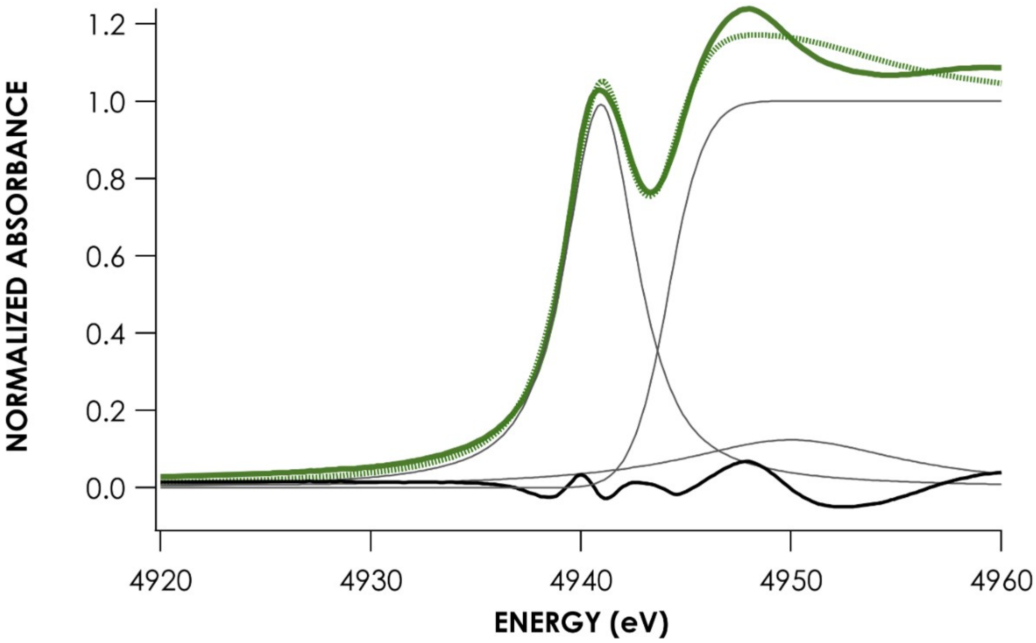

Figure S46. Overlay of the measured (solid) and fit (dashed) spectrum of **4**. Fit components including the edge jump plotted in grey. Residual absorption plotted in black.

Calculated spectra

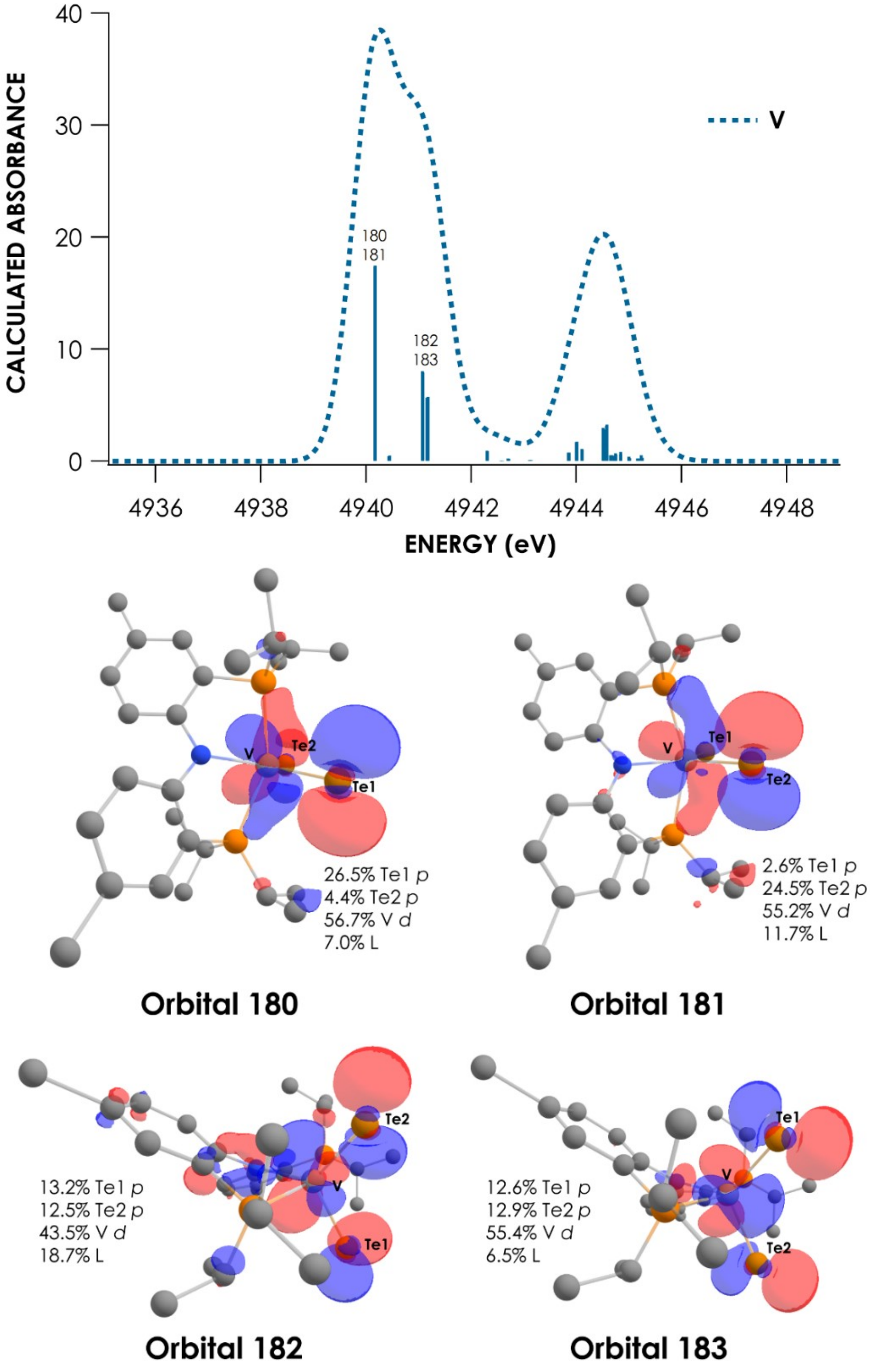

Figure S47. Calculated spectrum of **V** and key transitions (top). Associated orbitals with key transitions (bottom).

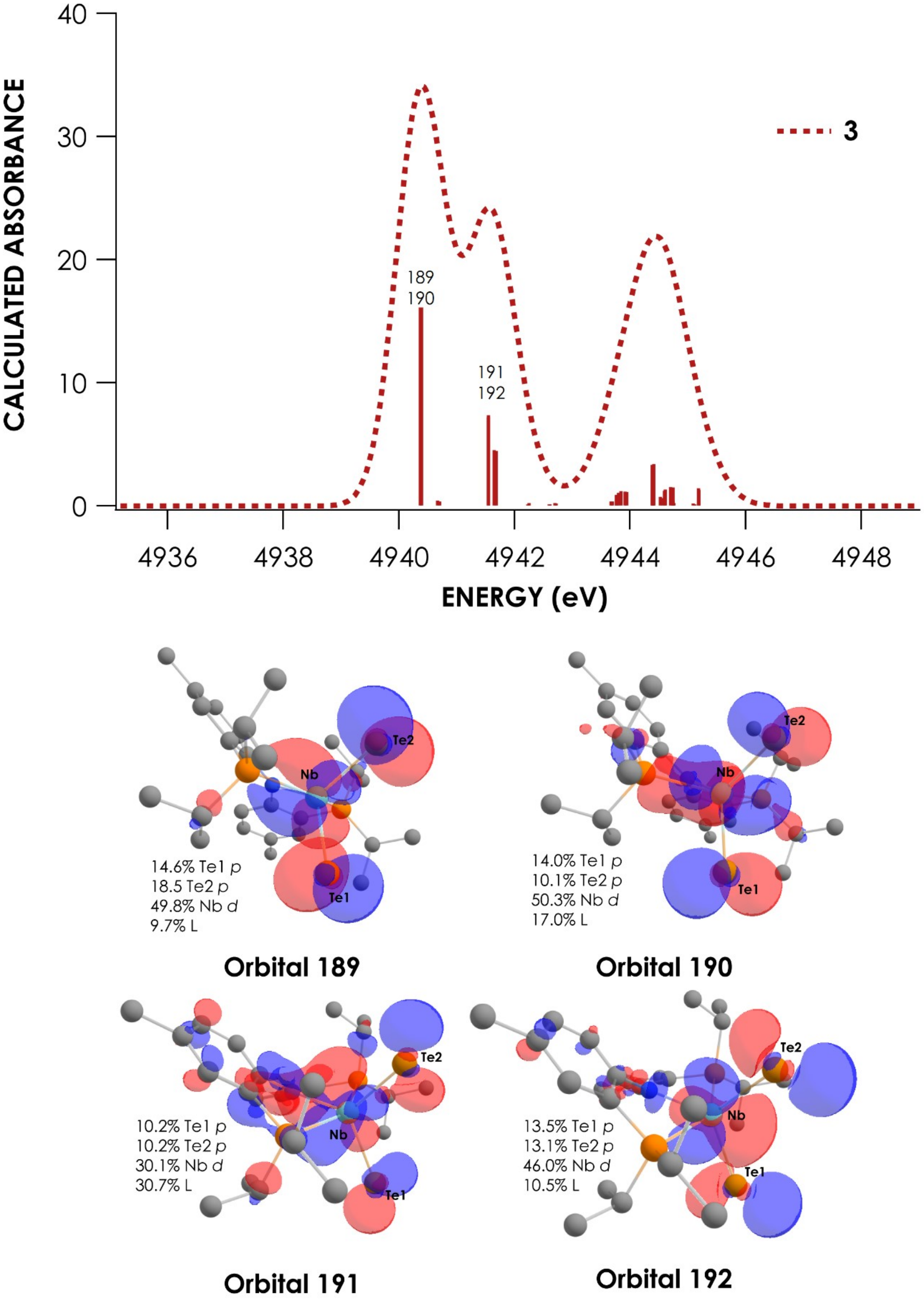

Figure S48. Calculated spectrum of **3** and key transitions (top). Associated orbitals with key transitions (bottom).

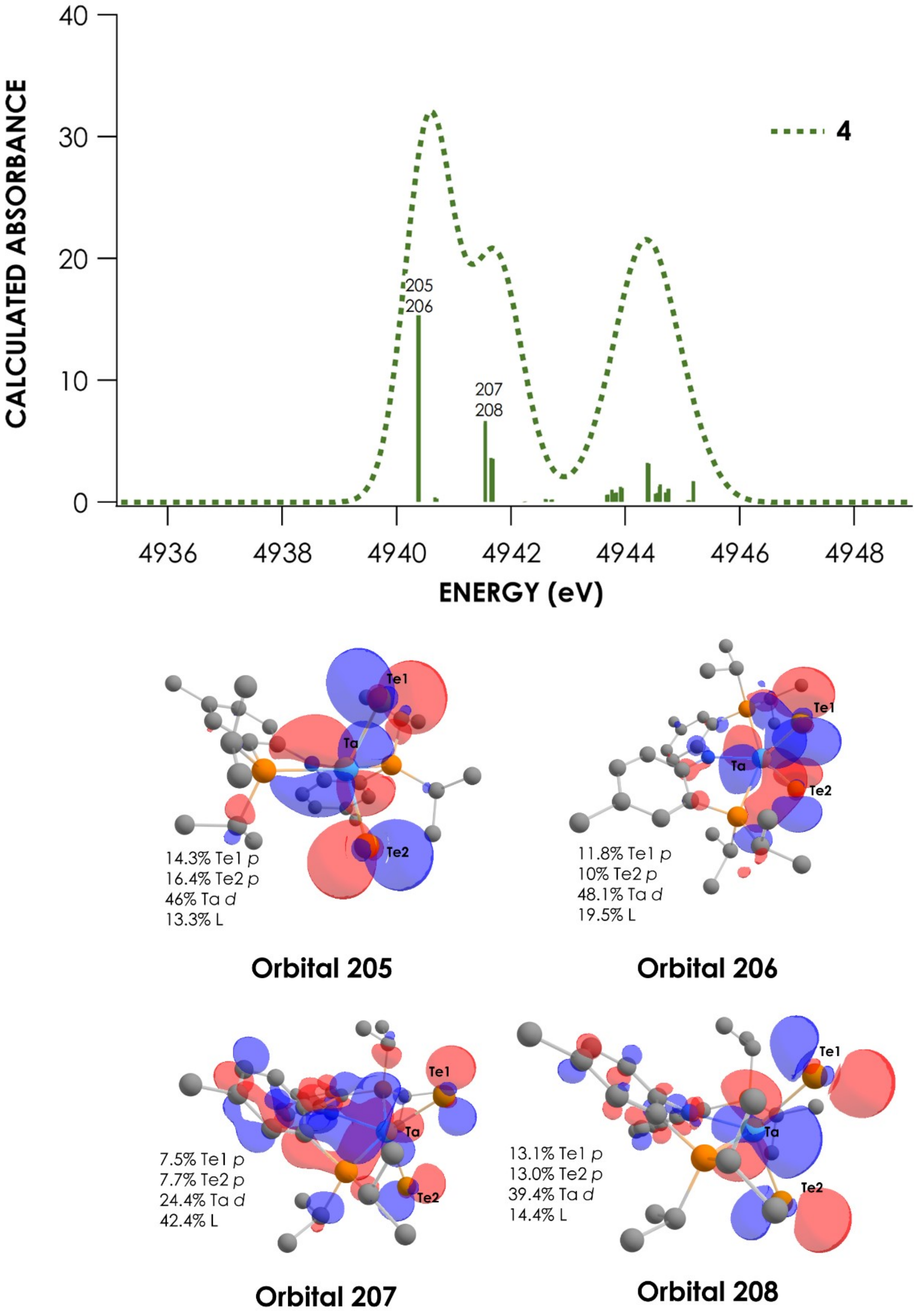

Figure S49. Calculated spectrum of **4** and key transitions (top). Associated orbitals with key transitions (bottom).

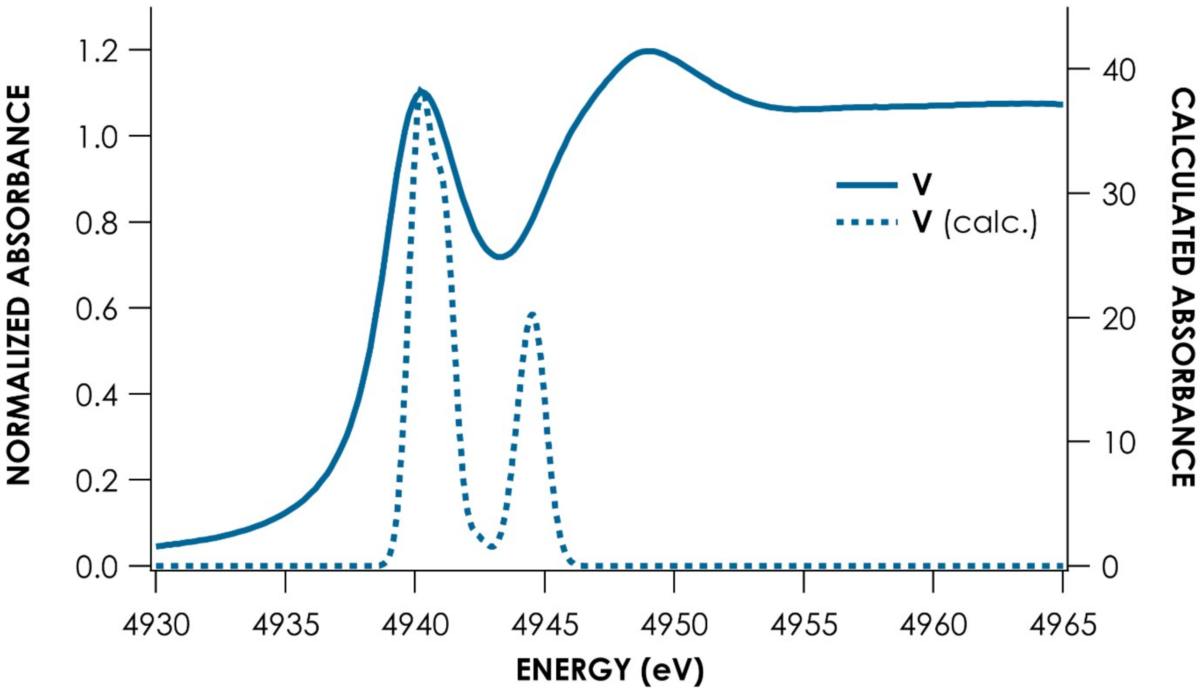

Figure S50. Overlay of the measured (solid) and calculated (dashed) spectra of **V**.

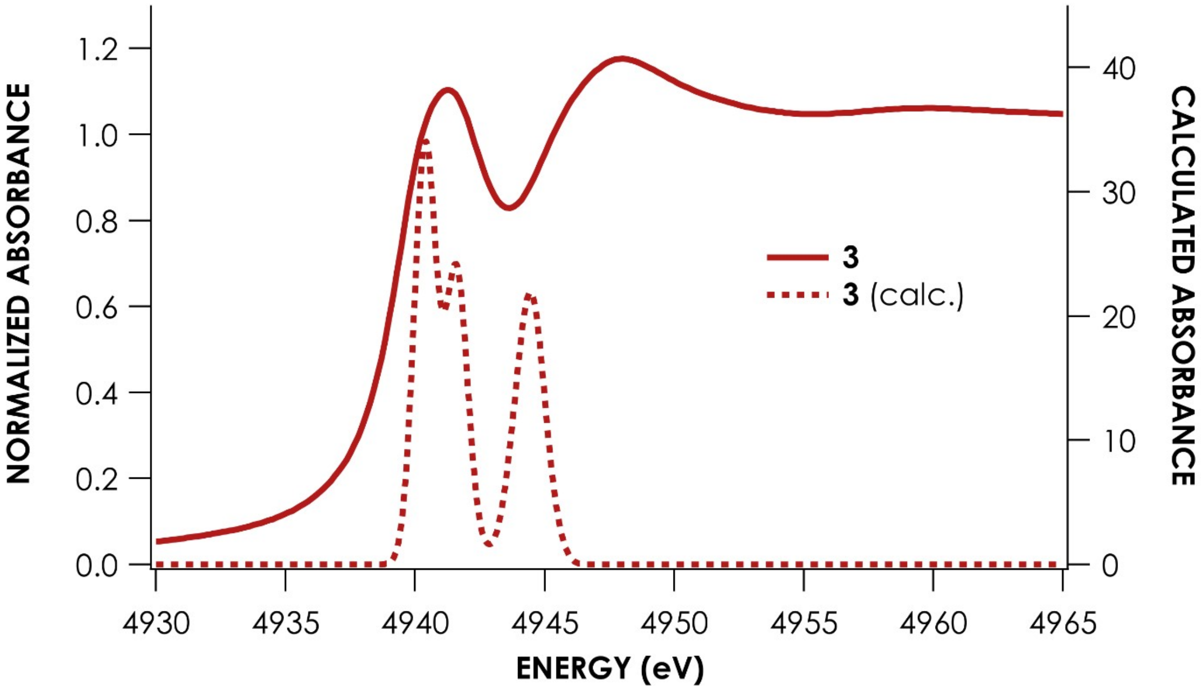

Figure S51. Overlay of the measured (solid) and calculated (dashed) spectra of **3**.

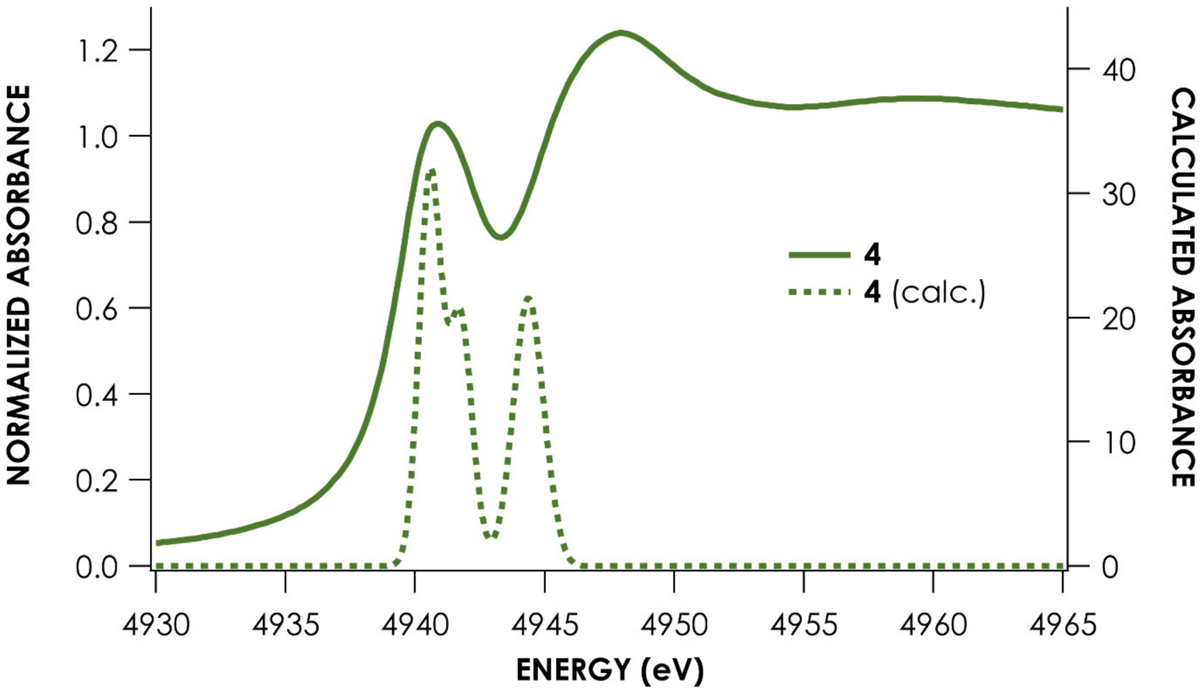

Figure S52. Overlay of the measured (solid) and calculated (dashed) spectra of **4**.

Table S9. Calculated average composition of the four acceptor orbitals.

	**Te *5p***	**M *d***	**L (total)**
**V**	27.3%	52.7%	11.0%
**3**	26.1%	44.1%	17.0%
**4**	23.5%	39.4%	22.4%

References

1. L. E. Manxzer, J. Deaton, P. Sharp, and R. R. Schrock in *Inorganic Syntheses*, Vol. 21 (Ed.:J.P. Fackler), **1982**, pp 135-140.
2. S. F. Pedersen, J. B. Hartung Jr., E. J. Roskamp, P. S. Dragovich, C. J. Ruffing, B. A. Klein in *Inorganic Syntheses*, Vol. 29 (Ed.: R. N. Grimes), **1992**, pp. 119-123.
3. J. Hicks, M. Juckel, A. Paparo, D. Dange, C. Jones, *Organometallics* **2018**, *37*, 4810-4813.
4. (a) L. Fan, B. M. Foxman, O. V. Ozerov, *Organometallics* **2004**, *23*, 326-328. (b) W. Weng, L. Yang, B. M. Foxman, O. V. Ozerov, *Organometallics* **2004**, *23*, 4700-4705.
5. F. P. Gabbaï, P. J. Chirik, D. E. Fogg, K. Meyer, D. J. Mindiola, L. L. Schafer, S.-L. You, *Organometallics* **2016**, *35*, 3255-3256.
6. U. J. Kilgore, X. Yang, J. Tomaszewski, J. C. Huffman, D. J. Mindiola, *Inorg. Chem*. **2006**, *45*, 10712.
7. L. C. H. Gerber, L. A. Watson, S. Parkin, W. Weng, B. M. Foxman, O. V. Ozerov, *Organometallics* **2007**, *26*, 4866-4868.
8. U. J. Kilgore, J. A. Karty, M. Pink, X. Gao, D. J. Mindiola, *Angew. Chem. Int. Ed. Engl.* **2009**, *48*, 2394-2397.
9. R. W. Gedridge, K. T. Higa, R. A. Nissan, *Magn. Reson. Chem*. **1995**, *33*, 441-448.
10. F. Sladky, B. Bildstein, C. Rieker, A. Gieren, H. Betz, T. Hübner, *J. Chem. Soc., Chem. Commun.* **1985**, 1800-1801.
11. C. Köllemann, D. Obendorf, F. Sladky, *Phosphorus Sulfur Relat. Elem*. **1988**, *38*, 69-77.
12. P. Zatsepin, J. H. Kim, M. R. Gau, P. J. Carroll, B. Pudasaini, M. H. Baik, D. J. Mindiola, *J. Am. Chem. Soc*. **2022**, *144*, 13066-13070.
13. CrysAlisPro 1.171.41.107a: Rigaku Oxford Diffraction, Rigaku Corporation, Oxford, UK, **2020**.
14. SCALE3 ABSPACK v1.0.7: an Oxford Diffraction program; Oxford Diffraction Ltd: Abingdon, UK, **2005**.
15. SHELXT v2018/2: G. M. Sheldrick*, Acta Crystallogr. A*, **2015**, *71*, 3-8.
16. SHELXL-2018/3: G. M. Sheldrick, *Acta Crystallogr. A*, **2015**, *71*, 3-8.
17. R. G. Parr, W. Yang, *Density Functional Theory of Atoms and Molecules*; Oxford University Press: New York, **1989**.
18. E. J. Baerends, D. E. Ellis, P. Ros, *Chem. Phys.* **1973**, *2*, 41–51.
19. E. J. Baerends, P. Ros, *Chem. Phys.* **1973**, *2*, 52–59.
20. H. J. J. te Riele, T. J. Dekker, H. A. van der Vorst, *Algorithms and Applications on Vector and Parallel Computers*; Elsevier: Amsterdam, **1987**.
21. G. te Velde, E. J. Baerends, *J. Comput. Phys.* **1992**, *99*, 84–98.
22. L. Versluis, T. Ziegler, *J. Chem. Phys.* **1988**, *88*, 322–328.
23. J. P. Perdew, K. Burke, M. Ernzerhof, *Phys. Rev. Lett.* **1996**, *77*, 3865–3868.
24. Y. Zhao, D. G. Truhler, *J. Chem. Phys.* **2006**, *125*, 194101.
25. P. J. Stephens, F. J. Devlin, C. F. Chabalowski, M. J. Frisch, *J. Phys. Chem.* **1994**, *98*, 11623–11627.
26. C. Adamo, V. Barone, *J. Chem. Phys.* **1999**, *110*, 6158–6170.
27. A. D. Becke, *J. Chem. Phys.* **1993**, *98*, 1372–1377.
28. V. N. Staroverov, G. E. Scuseria, J. Tao, J. P. Perdew, *J. Chem. Phys.* **2003**, *119*, 12129–12137.
29. Y. Zhao, D. G. Truhlar, *Theor. Chem. Acc.* **2008**, *120*, 215–241.
30. H. S. Yu, X. He, S. L. Li, D. G. Truhlar, *Chem. Sci.* **2016**, *7*, 5032–5051.
31. T. Yanai, D. P. Tew, N. C. Handy, *Chem. Phys. Lett.* **2004**, *393*, 51–57.
32. S. Hirata, M. Head-Gordon, *Chem. Phys. Lett.* **1999**, *314*, 291–299.
33. M. J. G. Peach, P. Benfield, T. Helgaker, D. J. Tozer, *J. Chem. Phys.* **2008**, *128*, 044118.
34. C. A. Guido, P. Cortona, B. Mennucci, C. Adamo *J. Chem. Theory Comput.* **2013**, *9*, 3118–3126.
35. M. J. G. Peach, C. R. Le Sueur, K. Ruud, M. Guillaume, D. J. Tozer, *Phys. Chem. Chem. Phys.* **2009**, *11*, 4465–4470.

36. B. Ravel, M. Newville, *J Synchrotron Radiat.* **2005**, *12*, 537-541
37. I. M. DiMucci, S. N. MacMillan, R. C. Walroth, K. M. Lancaster, *Inorg. Chem.* **2020**, *59*, 13416-13426.
38. F. Neese, *WIREs Computational Molecular Science* **2017**, *8*.
39. J. D. Rolfes, F. Neese, D. A. Pantazis, *J. Comput. Chem.* **2020**, *41*, 1842-1849.
40. A. D. Becke, *Phys. Rev. A Gen. Phys.* **1988**, *38*, 3098-3100.
41. F. Neese, *J. Comput. Chem.* **2003**, *24*, 1740-1747.
42. F. Neese, F. Wennmohs, A. Hansen, U. Becker, *Chem. Phys*. **2009**, *356*, 98-109.
43. E. van Lenthe, J. G. Snijders, E. J. Baerends, *J. Chem. Phys.* **1996**, *105*, 6505-6516.
44. V. Barone, M. Cossi, *J. Phys. Chem.* **1998**, *102*, 1995-2001.
